# New insights into the molecular basis of gametogenesis in the hybridogenetic water frog *Pelophylax esculentus*

**DOI:** 10.1038/s41598-026-37515-w

**Published:** 2026-02-04

**Authors:** Marcela Plötner, Martin Meixner, Albert J. Poustka, José H. Grau, Sabrina Theusner, Karsten Liere, Tino Schüllermann, Marie Doležálková‑Kaštánková, Lukas Choleva, Jörg Plötner

**Affiliations:** 1 Museum für Naturkunde, Leibniz-Institut für Evolutions- und Biodiversitätsforschung, Berlin, Germany; 2 Services in Molecular Biology GmbH, Rüdersdorf, Germany; 3 Dahlem Centre for Genome Research and Medical Systems Biology, Berlin, Germany; 4 Max-Planck-Institut für molekulare Genetik, Berlin, Germany; 5 Center for Species Survival, Smithsonian Conservation Biology Institute, Washington, USA; 6 Simple Constellation, Berlin, Germany; 7 Institute of Animal Physiology and Genetics CAS, Liběchov, Czech Republic; 8 Faculty of Science, Department of Biology and Ecology, University of Ostrava, Ostrava, Czech Republic

**Keywords:** Water frogs, *Pelophylax*, Gametogenic genes, Hybridogenesis, Genome exclusion, Clonal inheritance, Evolution, Genetics, Zoology

## Abstract

The molecular mechanisms underlying genome exclusion and clonal gamete formation in the germline of hybridogenetic water frog hybrids are poorly understood. Here, we characterize for the first time the coding sequences of 160 gametogenic genes from the European water frog species *Pelophylax lessonae* (LL) and *Pelophylax ridibundus* (RR). In addition, single nucleotide polymorphisms (SNPs) in 52 of these genes were analyzed in both parental species (60 LL, 252 RR) and in 340 diploid hybrids, *Pelophylax esculentus* (LR), sampled from population systems differing in genotypic composition, sex ratio, and inheritance modes. Ten of these genes showed associations with the population system and may therefore be related to the mode of inheritance, namely the exclusion of the *ridibundus* (R) genome in the *lessonae–esculentus* system and/or the *lessonae* (L) genome in the *ridibundus–esculentus* system. These genes are involved in diverse cellular processes, including spindle formation and chromosome movement during mitosis and meiosis, epigenetic silencing, cell cycle regulation, double-strand break repair and homologous recombination, including transposable element silencing.

Our results are consistent with the clonal inheritance patterns described for *P. esculentus* and suggest that genome exclusion is governed by complex genomic networks involving multiple genes and molecular factors. Furthermore, our data indicate that bidirectional gene flow between the L and R gene pools has played an important role in the evolution of the different clonal inheritance modes in *P. esculentus*, thereby contributing to the emergence of distinct population systems.

**Supplementary Information:**

The online version contains supplementary material available at 10.1038/s41598-026-37515-w.

## Introduction

Gametogenesis is a cell division and differentiation process that gives rise to reproductive cells (sperm and eggs). In sexually reproducing organisms, this process typically results in a halving of the chromosome number during a sub-process called meiosis. The correct execution of meiosis is essential for fertility, maintaining genome integrity, and ensuring the normal development (ontogenesis) of offspring^1^. Meiosis is associated with genetic recombination and random segregation of parental chromosomes, resulting in a large number of new allelic combinations in each generation. As a result, genetic variability increases, enhancing the adaptive potential of sexually reproducing populations. Thus, meiosis ensures both genetic variation and integrity^2,3^.

In contrast to the regular patterns of genome haploidization, a variety of plant and animal species exhibit non-random segregation of single chromosomes or even complete sets of maternal, or less frequently, paternal chromosomes (reviewed by^4–10^. In such species, meiotic divisions are modified or entirely absent, resulting in severely limited or no genetic recombination, causing a lack of genetic variability in gametes and offspring. These reproductive modes, referred to as non-Mendelian, asexual, or clonal, are often associated with unisexuality, i.e., the occurrence of only one sex, usually female^11,12^. As with organisms in which genes encoding cell cycle regulatory proteins are disrupted (null mutations), non-Mendelian species can help identify genes that play important roles in vertebrate gametogenesis, particularly those governing the poorly understood molecular mechanisms that initiate meiosis and regulate early meiotic events.

While asexual reproduction is relatively common in many unicellular and invertebrate organisms, often as part of their reproductive cycle with alternating sexual and asexual generations^13–17^, it is rare in vertebrates^18^. Only about 0.1% of extant vertebrate taxa reproduce asexually, i.e. by parthenogenesis, gynogenesis, kleptogenesis, or hybridogenesis (reviewed e.g., by^12,19^. Compared to pure clonal reproductive modes such as parthenogenesis or gynogenesis, hybridogenesis combines elements of both clonal and Mendelian inheritance. First detected in Mexican mollies of the genus *Poeciliopsis*^20^, hybridogenesis has also been reported in European water frogs (genus *Pelophylax*)^21–23^, in *Batura toads* (*Bufotes baturae*^24^, Mediterranean stick insects (genus *Bacillus*)^25^, Iberian minnows of the *Squalius alburnoides* complex^26–28^, Australian carp gudgeons (genus *Hypseleotris*)^29^, marine reef fishes of the genus *Hexagrammos*^30,31^, spined loaches (*Cobitis*^32^, and loaches (*Misgurnus*^33,34^.

In water frogs, three hybridogenetic hybrid forms have been identified. *Pelophylax esculentus* (genotype LR), which arose (and still arises) by hybridization between *Pelophylax ridibundus* (RR) and *Pelophylax lessonae* (LL), is the best known of these; the others are *Pelophylax grafi* (*P. ridibundus* x *Pelophylax perezi*) and *Pelophylax hispanicus* (*P. ridibundus* x *Pelophylax bergeri*). Diploid *P. esculentus* (LR) exclude one parental genome in their germline and clonally transmit the remaining genome to gametes, which are fertilized by the coexisting sexual host parental species – *P. lessonae* in the so-called *lessonae*-*esculentus* (L-E) system and *P. ridibundus* in the *ridibundus*-*esculentus* (R-E) system^35–37^. Consistent with the genotypic structure of the population system, almost all diploid LR hybrids in the L-E system inherit the *ridibundus* (R) genome, while gametes of hybrids in the R-E system contain either unrecombined *lessonae* (L) or R genomes. In both systems, hybridity is restored in each generation by combining clonal genomes inherited by hybrids with Mendelian genomes provided by the parental species. Additionally, *P. esculentus* can form all-hybrid (E) populations, where triploid genotypes with two L genomes and one R genome (LLR) typically occur at high frequencies^36–42^. In these populations, hybrid reproduction primarily relies on crosses between LR females, which produce haploid R and/or diploid LR ova, and LLR males, serving as donors of haploid L sperm^36–40,43^.

Despite five decades of intensive research, the molecular mechanisms underlying genome exclusion and clonal gamete formation in the germline of hybridogenetic water frog hybrids remain poorly understood. Unlike other organisms where chromosome elimination during mitotic divisions has been documented (e.g. *Poeciliopsis monacha-lucida* hybrids^44^, no irregularities in the formation of the spindle apparatus (e.g., unipolar spindle formation) have been observed in *P. esculentus*^45^. Several observations indicate that in *P. esculentus*, uniparental chromosome elimination is a gradual process initiated at early stages of ontogenesis, particularly in gonads of tadpoles at approximately Gosner stage 28^46^. This stage corresponds to the onset of sexual differentiation and is marked by high mitotic activity of the gonocytes^45,47,48^.

It has been hypothesized that genome exclusion in Western Palearctic water frogs is driven by the R genome, as all hybridogenetic taxa typically produce R gametes^49,50^. However, this hypothesis is contradicted by observations that many LR hybrids in the R-E system, as well as some hybrids in all-hybrid populations, inherit the L while excluding the R genome, or even produce both R and L gametes^51–56^. These findings suggest that putative inducing factors, or segregation distorters, may also reside on the L genome^36,51^. Accordingly, it is plausible to postulate the existence of two distinct types of L genomes: those carrying such inducing factors and those lacking them.

Interactions between inducing factors and gametogenic genes may have favored certain alleles or promoted the emergence of new, population system-specific variants. To test this hypothesis, we analyzed single nucleotide polymorphisms (SNPs) across 52 gametogenic genes of 416 diploid water frogs (LL, RR, LR) sampled from L-E, R-E, all-*ridibundus*, and all-hybrid populations. A primary objective of this study was to identify genes associated with population systems that could serve as candidates linked to genome exclusion or potentially act even as key regulators of this process. In addition, we provide the coding sequences for 160 gametogenic genes of Central European water frogs as a valuable resource for future molecular studies on hybridogenesis.

## Results

### Characteristics of gametogenic genes

A total of 620 sequences from 160 gametogenic genes were identified in our germline (testis) transcriptome library, comprising four transcriptomes: one from *P. lessonae* (LL) and three from *P. ridibundus* (RR1–RR3). Of these, 160 coding sequences (CDS) were obtained from LL and 460 from RR (Table S1, Supplementary Material 1; Supplementary Material 2). For each CDS, we calculated the coding sequence length (CDSL) and GC content. Intron-exon boundaries were determined using Splign^57^ and a draft genome assembly of *P. lessonae* (Table S2, Supplementary Material 1).

CDSL ranged from 225 bp (*anapc13*) to 8,049 bp (*itpr1*). Thirty-two genes exhibited interspecific length polymorphisms (3–63 bp), whereas intraspecific length polymorphisms were detected in seven genes. In LL, two *dnmt1* alleles were identified (4,470 and 4,476 bp). In RR, six genes showed intraspecific length variation: *ago2* (2,622 vs. 2,595 bp), *b*c*ar1* (2,871 bp vs. 2,856 bp), *bub1* (3,345 bp vs. 3,348 bp), *pms2* (2,517 bp vs. 2,520 bp), *nbn* (2,292 bp vs. 2,289 bp), and *tdrd1* (4,017 bp vs. 4,005 bp).

The GC content ranged from 34.9% (*sycp1*) to 58.4% (*zfp36*). There were no significant differences in mean GC content between *P. lessonae* (x̅ = 46.1% ± 4.6%, x͂ = 45.2%) and *P. ridibundus* (x̅ = 45.9% ± 4.5%, x͂ = 45.1%), or between *Pelophylax* spp. (x̅ = 46.0% ± 4.5%, x͂ = 45.1%) and *Xenopus tropicalis* (x̅ = 45.9% ± 5.3%, x͂ = 45.0%).

**Fig. 1:**
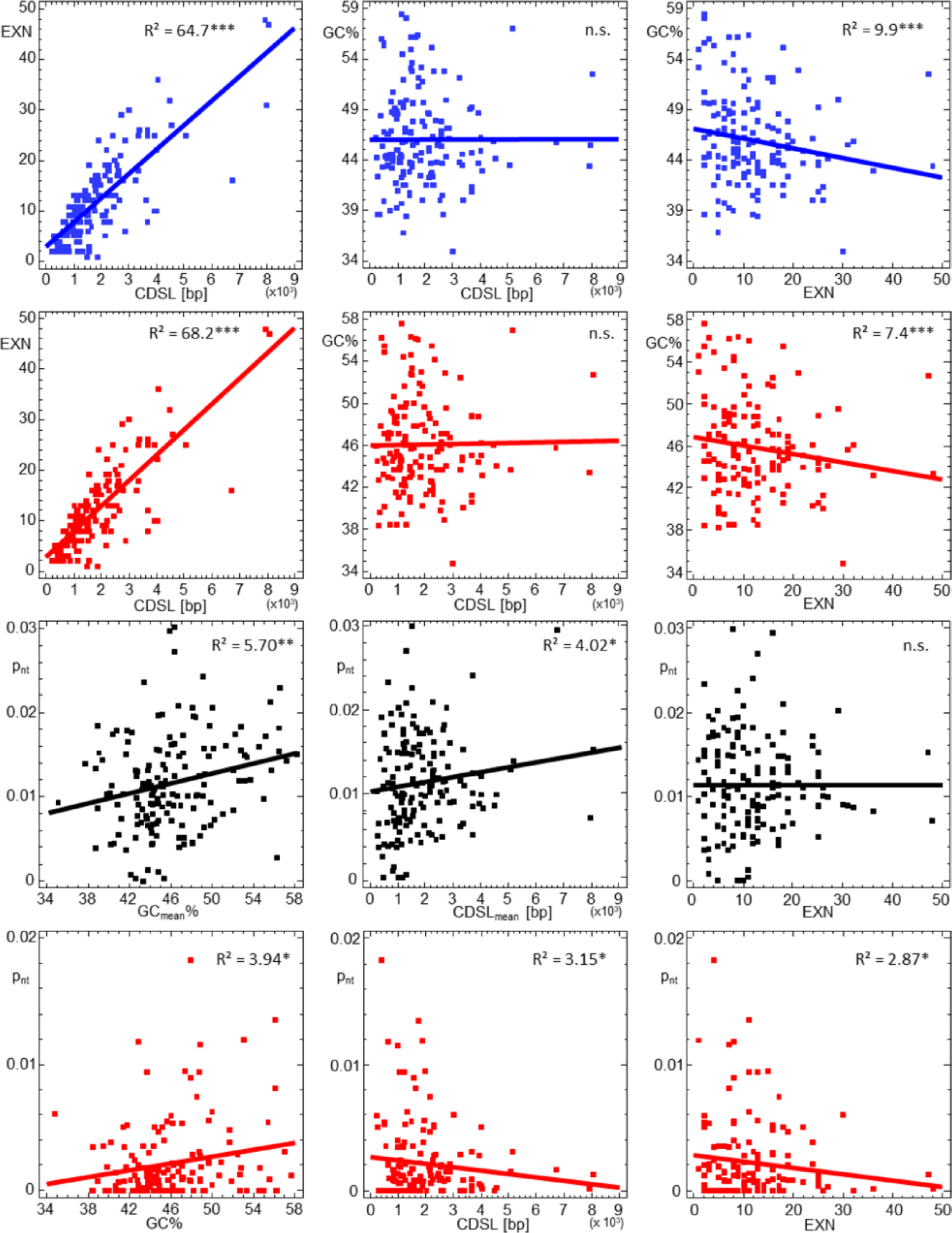
Relationships between coding sequence length (CDSL), exon number (EXN), GC content (GC), and uncorrected p-distances (p_nt_) of 160 gametogenic genes obtained from germline transcriptomes (testes) of *P. lessonae* (blue) and *P. ridibundus* (red). P-distances were calculated for both intraspecific comparisons (RR1–RR2–RR3; red) and interspecific comparisons (LL–RR; black). R² values were derived from regression models (Tables S3 and S4, Supplementary Material 1. Significance levels: *p ≤ 0.05, **p ≤ 0.01, ***p ≤ 0.001.

The exon number (EXN) could be determined for 156 genes, ranging from two to 48. EXN showed a positive correlation with CDSL and a weak negative correlation with GC content in both LL and RR transcriptomes. No significant correlation was observed between GC content and CDSL (Fig. 1, Table S3, Supplementary Material 1).

### Inter- and intraspecific genetic divergence

We assessed genetic divergence (uncorrected p-distance) between homologous transcripts (CDS) to estimate the degree of conservation of the genes under study, using comparisons among *P. lessonae*, *P. ridibundus*, and *X. tropicalis*. Between *Pelophylax* spp. and *X*. *tropicalis*, nucleotide divergence (p_nt_) ranged from 7.7 to 55.9%, with corresponding amino-acid divergence (p_aa_) of 0–69.3%. Three genes (*calm*, *h3.3*, and *skp1*) exhibited no amino acid differences. Interspecific comparisons between LL and RR orthologs revealed considerably lower divergence, with p_nt_-values of 0–3.2% and p_aa_-values of 0–5.4%. The orthologs of three genes (*calm*, *exd1*, *rad51*) exhibited no nucleotide differences, and those of 23 genes were identical in their amino acid composition (Supplementary Material 3).

**Fig. 2:**
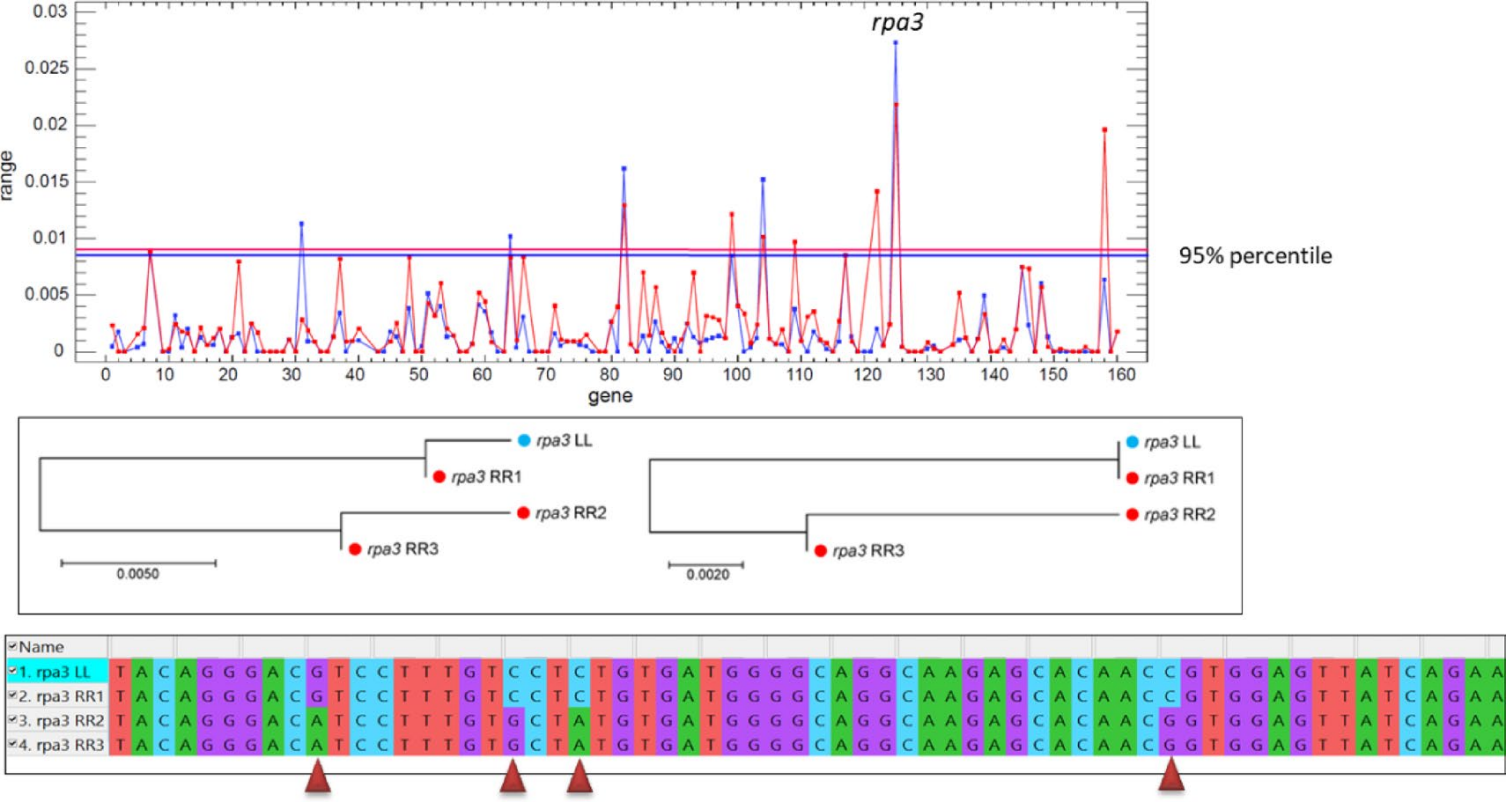
Evidence for introgression of an L-specific *rpa3* allele into the *ridibundus* gene pool. Upper: Ranges of variation of uncorrected p-distances calculated for intraspecific (red) and interspecific (blue) CDS comparisons. Values outside the 95th percentile indicate unusually high intraspecific or low interspecific divergence. Middle: Neighbor-joining trees for *rpa3* based on uncorrected p-distances from nucleotide sequences (left) and corresponding amino acid sequences (right). Lower: Partial alignment of the *rpa3* CDS, with substitutions indicated by arrows. Both the gene trees and the alignment demonstrate a high degree of similarity between the L allele and the R allele of individual RR1, consistent with introgression.

**Fig. 3:**
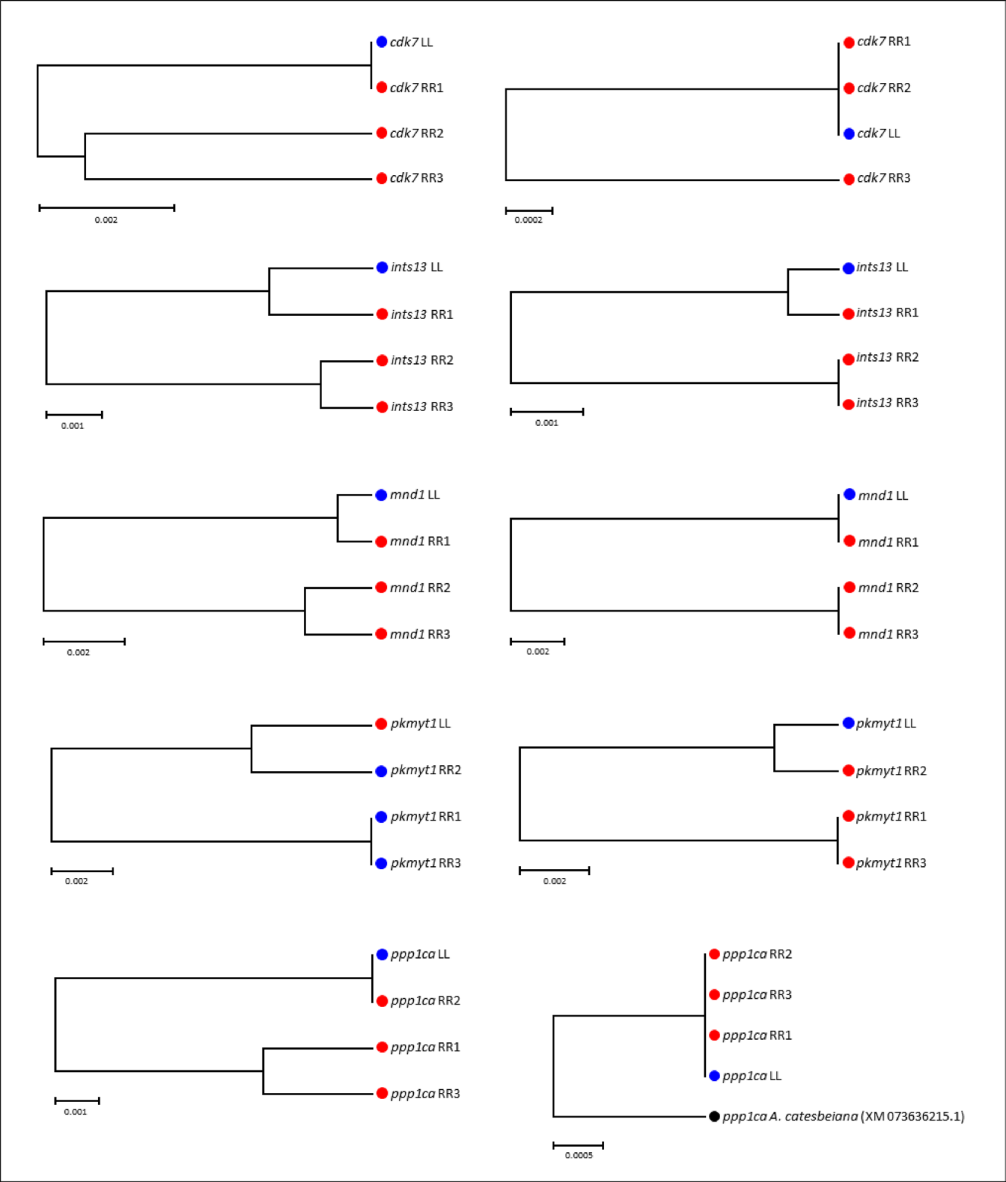
Putative introgression of *lessonae*-specific alleles into the *ridibundus* gene pool. UPGMA trees of coding sequences are shown for nucleotides (left) and amino acids (right). Some *ridibundus*-specific alleles (red circles) are highly similar to, or identical with, their *lessonae* orthologs (blue circles). Given the estimated divergence time between *P. lessonae* (LL) and *P. ridibundus* (RR) of 8–17 million years^58,59^, gene flow is the most parsimonious explanation for these genealogies. Scale bar: uncorrected p-distance. Gene names are listed in Table S1, Supplementary Material 1.

Pairwise intraspecific comparisons of R-specific alleles revealed p_nt_-values of 0–2.7% and p_aa_-values of 0–2.5% (Table S2, Supplementary Material 1; Supplementary Material 3). While most genes displayed low variability, several exhibited unusually high intraspecific divergence, with distance ranges exceeding the 95% percentile. In these cases, at least one R allele was highly similar or even identical to its L ortholog (Figs. 2 and 3; Fig. S1, Supplementary Material 4). Conversely, 41 genes showed no intraspecific nucleotide variation, and 87 were identical at the amino acid level.

In addition, p_nt_ between L and R orthologs was significantly positively correlated with both CDSL and GC content. Similarly, in intraspecific comparisons among RR transcripts p_nt_ correlated positively with CDSL, but negatively with both GC and EXN. These correlations were modest, however, explaining only 2.9–5.7% of the variability in p_nt_ (Fig. 1; Table S4, Supplementary Material 1).

To compare distinct genotypes from different population systems and geographic origins, single nucleotide polymorphisms (SNPs) of 52 gametogenic genes were analyzed (Table S5, Supplementary Material 1). The dataset included 652 water frogs from L-E, R-E, all-hybrid, and all-R populations across Europe (Table S6, Supplementary Material 1; Fig. S3, Supplementary Material 4). GTseq analysis identified a total of 131 SNPs, comprising 22 (16.8%) intraspecific SNPs within L sequences and 58 (44.3%) within R sequences; 66 SNPs (50.4%) were classified as species-specific (Tab. S5, Supplementary Material 1). The allele frequency distributions of species-specific SNPs were typically zero- or one-inflated in the parental genotypes, yielding intermediate frequencies (0.5) in LR hybrids (Fig. S4, Supplementary Material 4).

### Evidence for genetic introgression

Both transcriptome and GTseq (SNP) data provide evidence for introgression, i.e., gene flow between the L and R genomes. CDS analysis revelaed high similarity or even identity between L- and R-specific orthologs for six genes (Figs. 2 and 3). Additional evidence for gene flow was found in the SNP dataset. For example, in the CDS of *adcy9*, the L-specific nucleotides A (SNP1) and C (SNP2) were absent in RR genotypes, except in frogs from Karsibor (PL) and Lebus (DE), which had frequencies of 0.11 and 0.08, respectively. Based on 16 SNPs of 13 genes, obtained from 460 RR individuals of 11 populations and 116 LL individuals of three populations, mean introgression rates were estimated at 0.01 ± 0.036 for RR and 0.003 ± 0.0134 for LL (Table 1).

**Table 1:** Frequencies of *lessonae*-specific nucleotides (nu_L_) in *P. ridibundus* (RR) and *P. lessonae* (LL). Values > 0 in RR and < 1 in LL may indicate introgression (highlighted in bold).

SNP	nu_L_	Gene	Genotype (Population System)																	
			RR (all-R)						RR (R-E)					$\:\overline{\boldsymbol{f}}$_**RR**_	IR_**SNP**_ (RR)	LL (L-E)			$\:\overline{\boldsymbol{f}}$_**LL**_	IR_**SNP**_ (LL)
			Cit	Zel	Sur	Kap	Bul	Olt	Alb	Dol	Kos	Kar	Leb			Trn	Cet	Ger		
1	A	*adcy9*	0.00	0.00	0.00	0.00	0.00	0.00	0.00	0.00	0.00	**0.11**	**0.08**	0.016	0.016	1.00	1.00	1.00	1.000	0.000
2	C		0.00	0.00	0.00	0.00	0.00	0.00	0.00	0.00	0.00	**0.11**	**0.08**	0.016	0.016	1.00	1.00	1.00	1.000	0.000
24	C	*hat1*	**0.09**	0.00	0.00	0.00	0.00	0.00	0.00	0.00	0.00	0.00	0.00	0.008	0.008	1.00	1.00	1.00	1.000	0.000
25	C	*henmt1*	0.00	0.00	0.00	0.00	0.00	0.00	0.00	0.00	0.00	0.00	**0.02**	0.002	0.002	1.00	1.00	1.00	1.000	0.000
30	C	*hormad1*	0.00	0.00	0.00	0.00	0.00	0.00	0.00	**0.04**	0.00	0.00	**0.04**	0.007	0.007	1.00	1.00	1.00	1.000	0.000
32	A		0.00	0.00	0.00	0.00	0.00	0.00	0.00	**0.04**	0.00	0.00	**0.04**	0.007	0.007	1.00	1.00	1.00	1.000	0.000
45	T	*itpr1*	1.00	1.00	1.00	1.00	1.00	1.00	1.00	1.00	1.00	1.00	1.00	1.000	0.000	**0.06**	0.00	n.a.	0.030	0.030
61	G	*map3k7*	**0.04**	0.00	0.00	0.00	0.00	0.00	0.00	0.00	0.00	0.00	0.00	0.004	0.004	1.00	1.00	1.00	1.000	0.000
78	G	*msh2*	1.00	1.00	1.00	1.00	1.00	**0.72**	1.00	1.00	1.00	1.00	1.00	0.975	0.025	0.00	0.00	0.00	0.000	0.000
84	G	*parn*	1.00	1.00	n.a.	1.00	1.00	1.00	1.00	1.00	1.00	1.00	1.00	1.000	0.000	**0.05**	0.00	n.a.	0.025	0.025
99	C	*plk1*	0.00	0.00	0.00	0.00	**0.06**	0.00	0.00	0.00	0.00	0.00	0.00	0.005	0.005	1.00	1.00	**0.95**	0.983	0.017
114	A	*rbbp8*	**0.05**	0.00	0.00	0.00	0.00	0.00	0.00	0.00	0.00	0.00	0.00	0.005	0.005	1.00	1.00	1.00	1.000	0.000
124	C	*smc1a*	0.00	0.00	**0.21**	0.00	0.00	**0.06**	0.00	0.00	0.00	0.00	0.00	0.025	0.025	1.00	1.00	1.00	1.000	0.000
125	C		0.00	0.00	**0.21**	0.00	0.00	**0.06**	0.00	0.00	0.00	0.00	0.00	0.025	0.025	1.00	1.00	1.00	1.000	0.000
129	T	*ywhaz*	**0.95**	1.00	1.00	1.00	1.00	1.00	1.00	1.00	1.00	1.00	1.00	0.995	0.005	0.00	0.00	0.00	0.000	0.000
131	C	*zfp36*	0.00	0.00	0.00	0.00	0.00	0.00	0.00	0.00	0.00	**0.05**	**0.06**	0.010	0.010	1.00	1.00	1.00	1.000	0.000
		**IR**_**POP**_	0.014	0.00	0.028	0.00	0.004	0.025	0.00	0.005	0.00	0.016	0.02			0.007	0.00	0.004		
	IR (mean)													0.01 ± 0.036		0.003 ± 0.0134				

Introgression rates (IR) were estimated for populations (IR_POP_) and for SNPs (IR_SNP_). $\:\overline{f}$_RR_ and $\:\overline{f}$_LL_ represent the arithmetic means of SNP frequencies across all populations. IR_mean_ denotes the arithmetic mean of the introgression rates across all SNPs and populations of RR and LL, respectively. Population abbreviations are given in Table S6, Supplementary Material 1).

### Associations between SNPs and population systems

**Fig. 4:**
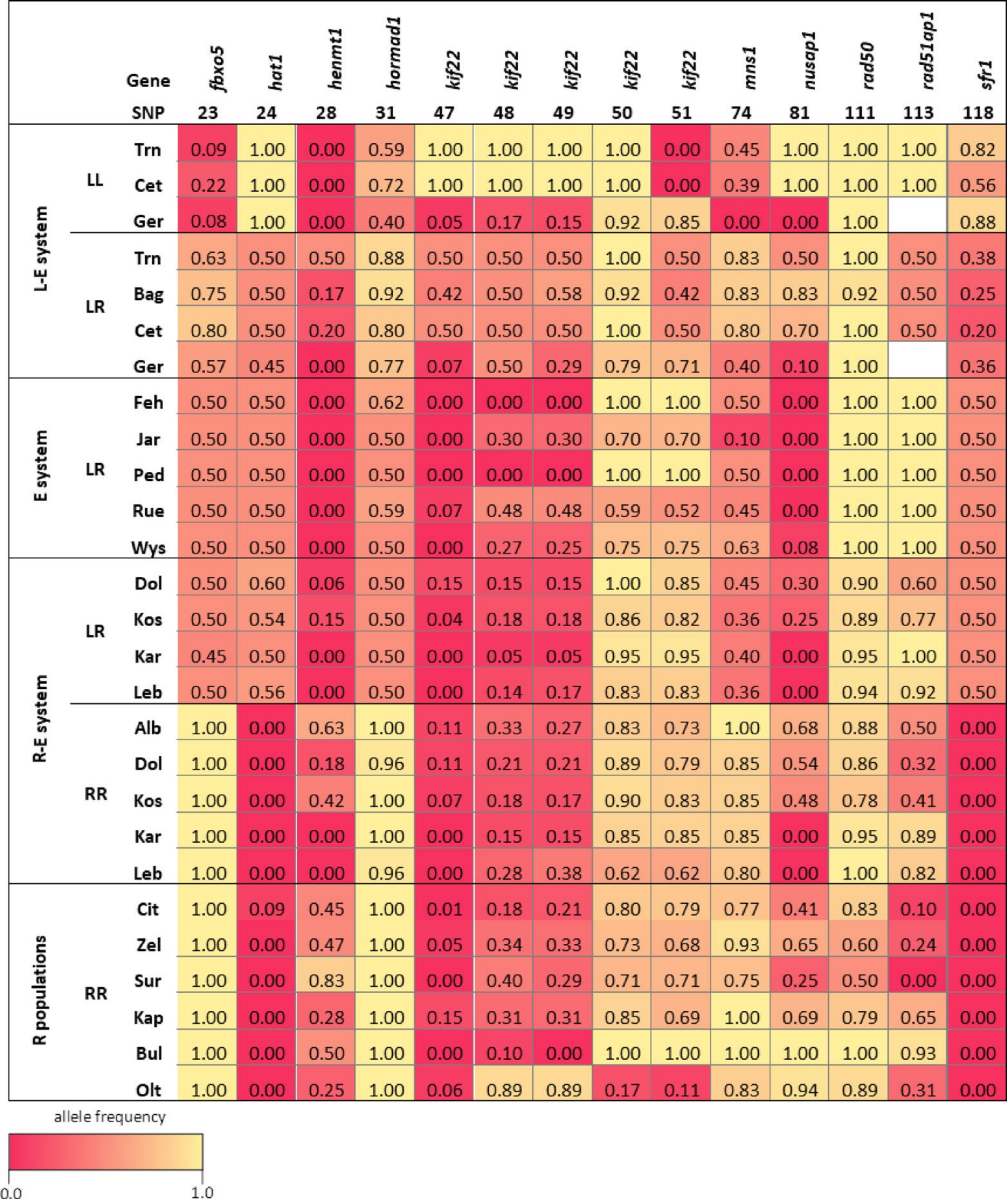
Heatmap of SNP frequencies associated with population systems. For both LL and LR, clear differences were observed between Czech/Croatian and German *lessonae*-*esculentus* (L-E) populations. Additionally, several SNPs exhibited frequency differences between LR hybrids from the diploid L-E system and conspecifics from the *ridibundus*-*esculentus* (R-E) system and all-hybrid (E) populations. In contrast, SNP frequencies in *P. ridibundus* (RR) did not show system-specific patterns (Table S7, Supplementary Material 1). Full population names are provided in Table S6, Supplementary Material 1.

To assess whether SNPs allow genotype assignment to specific population systems (POPSYS), we first conducted non-parametric Kruskal-Wallis tests. Fourteen SNPs from LR individuals exhibited significant system-specific frequency differences. Of these, eleven SNPs indicated that LR genotypes from all-hybrid populations are more closely related to LR genotypes from the R-E system than to diploid hybrids from L-E populations of the Czech Republic and Croatia. Comparisons between German and Czech/Croatian L-E populations further revealed clear differences for both LL and LR genotypes. By contrast, RR genotypes from all-R and R-E populations displayed similar SNP frequencies (Fig. 4; Table S7, Supplementary Material 1).

**Fig. 5:**
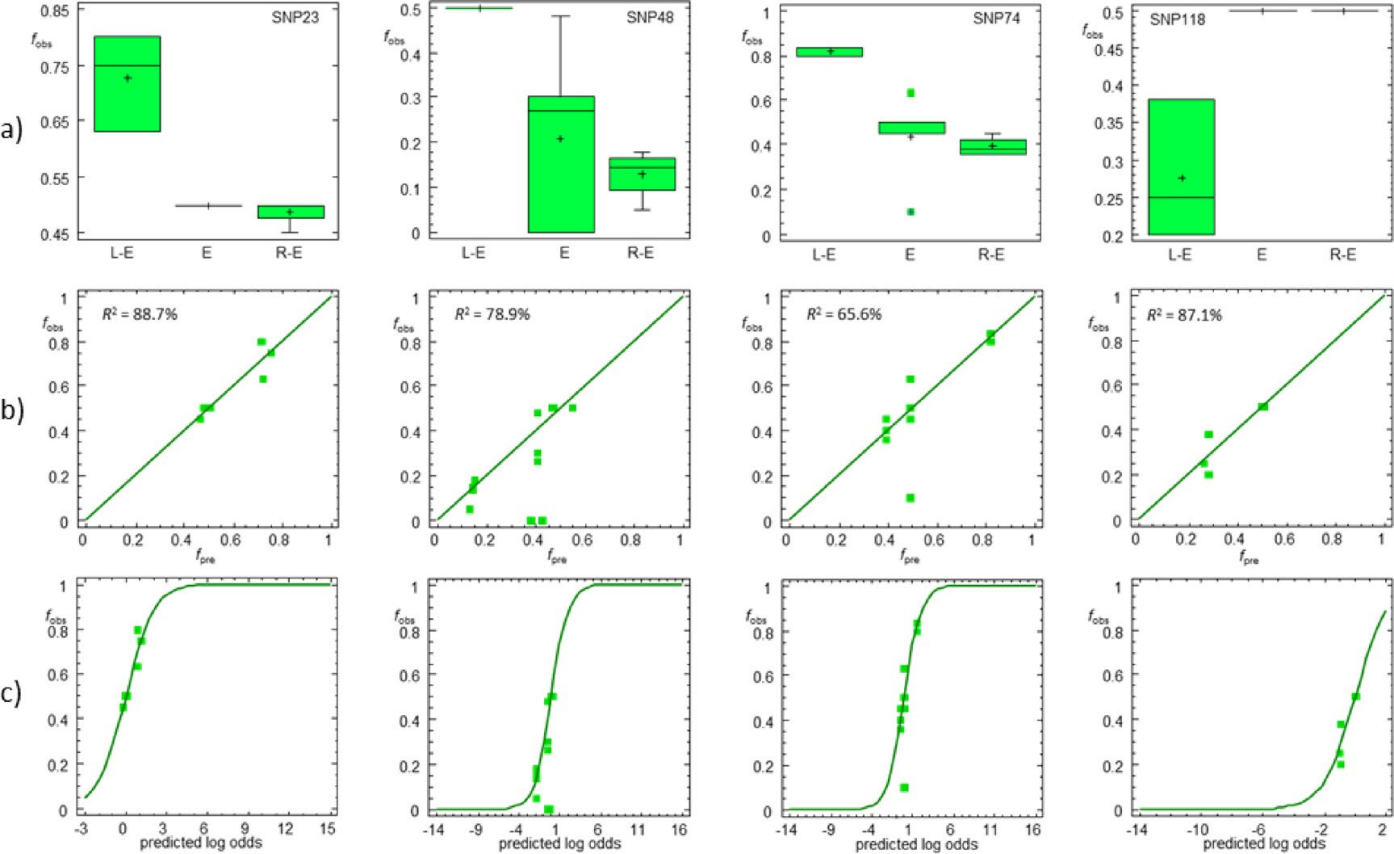
Examples of SNPs from LR individuals whose frequencies (*f*) are associated with the population system (POPSYS). (**a**) Box-whisker-plots showing that LR individuals from all-diploid L-E populations differ significantly from conspecifics of R-E and all-hybrid populations. (**b**) Observed versus predicted *f* values from logistic regression models, where the categorical variable POPSYS was the only significant predictor of *f*. (**c**) Observed SNP frequencies versus predicted log-odds. The log-odds equal the logistic transformation, which is an exponential function of the predictor variables. For additional information, see Tables S8–S9, Supplementary Material 1. L-E: *lessonae*-*esculentus* populations, R-E: *ridibundus*-*esculentus* populations, E: all-hybrid (*esculentus*) populations.

Given the biased geographic distribution of the populations and population systems studied (Fig. S3, Supplementary Material 4), logistic regressions were performed for each SNP to separate the effects of geographic variables (longitude, latitude) from the categorical predictor variable POPSYS. For SNP alleles showing significant median frequency differences (Table S7, Supplementary Material 1), the logistic models identified POPSYS, alone or in combination with one or both geographic variables, as a significant predictor of SNP frequencies, except for SNP24 and SNP89. In two cases (SNP28 and SNP49), the logistic function did not adequately fit the observed data. For seven SNPs whose frequencies conformed to a logistic model, POPSYS was the sole significant predictor, as indicated by type III sum of squares statistics. These SNPs correspond to the genes *fbxo5*, *kif22*, *mns1*, and *sfr1*. Notably, for *kif22*, all analyzed SNPs (47–51) identified POPSYS as the only significant predictor, including SNP50, whose system-specific frequencies were not significantly different (Fig. 5; Table 2; Tables S8–S9, Supplementary Material 1).

**Table 2:** Single nucleotide polymorphisms (SNPs) of gametogenic genes associated with the categorical variable population system (POPSYS) identified by logistic regression.

Gene	Exon	SNP	Logistic regression (Weighted Least Squares)									Median (f)			K-W	
			Model (ANOVA)					Type III Sum of Squares								
			F	p	R² [%]	R²_**adj**_ [%]	p(Χ²)	Variable	F	d.f.	p	L-E	E	R-E	H	p
*fbxo5*	1	23	13.74	0.002	88.7	82.2	0.956	POPSYS	14.58	2	0.003	0.750	0.500	0.500	9.13	0.010
*hat1*	9	24	3.58	0.068	67.2	48.4	0.764	POPSYS	4.46	2	0.056	0.500	0.500	0.550	7.16	0.028
*henmt1*	6	**28**	5.25	0.028	75.0	60.7	< 0.001	LAT	7.33	1	0.030	0.200	0.000	0.030	8.82	0.012
								POPSYS	9.93	2	0.009					
*hormad1*	7	31	33.62	< 0.001	95.0	92.2	0.992	LAT	9.09	1	0.020	0.880	0.500	0.500	8.44	0.015
								LON	15.17	1	0.006					
								POPSYS	45.02	2	< 0.001					
*kif22*	2	47	44.46*	< 0.001	90.8	88.8	0.098	POPSYS	44.46	2	< 0.001	0.500	0.000	0.020	7.48	0.024
	5	48	6.62	0.016	79.1	67.2	0.114	POPSYS	10.84	2	0.007	0.500	0.270	0.145	6.48	0.039
		49	6.71	0.015	79.3	67.5	0.011	POPSYS	10.20	2	0.008	0.500	0.250	0.160	6.41	0.040
	6	50	13.97*	0.002	75.6	70.2	0.054	POPSYS	13.97	2	0.002	1.000	0.750	0.905	1.86	0.394
	6	51	6.71	0.015	79.3	67.5	0.221	POPSYS	10.20	2	0.008	0.500	0.750	0.840	6.41	0.040
*mns1*	7	74	3.38	0.077	65.9	46.4	0.831	POPSYS	5.52	2	0.036	0.830	0.500	0.380	7.38	0.025
*nusap1*	8	81	48.93*	< 0.001	94.8	92.9	0.344	LAT	11.15	1	0.010	0.700	0.000	0.125	7.73	0.021
								POPSYS	20.37	2	< 0.001					
*rad50*	5	**111**	44.64	< 0.001	96.2	94.1	0.979	POPSYS	10.57	2	0.008	1.000	1.000	0.920	7.75	0.021
*rad51ap1*	5	113	6.82*	0.014	71.9	61.4	0.945	LON	6.10	1	0.039	0.500	1.000	0.845	9.47	0.009
								POPSYS	6.75	2	0.019					
*sfr1*	2	**118**	11.80	0.003	87.1	79.7	> 0.999	POPSYS	12.81	2	0.005	0.250	0.500	0.500	10.73	0.005

SNPs causing amino acid substitutions are shown in bold. Latitude (LAT) and longitude (LON) were included in the model to account for potential geographic effects on SNP frequency (*f*), with a minimum *f*-value of 10^− 10^. Kruskal-Wallis (K-W) tests detected significant system-specific differences for all SNPs except SNP50. Logistic regressions identified a significant effect of POPSYS on *f* for all SNPs except SNP24 (F = 3.58, *p* = 0.68). For SNPs 28 and 49, the logistic model did not adequately fit the observed data (*p* < 0.05, Χ² statistic). Logistic models were based on type III sum of squares; forward selection models are indicated with *. R² represents the percentage of variability in SNP frequencies explained by the regression model; R²_adj_ allows comparisons of models with different numbers of coefficients. d.f.: degrees of freedom. L-E: *lessonae*-*esculentus* populations, R-E: *ridibundus*-*esculentus* populations, E: all-hybrid (*esculentus*) populations.

Multilocus genotypes (MLG) were constructed from SNPs associated with POPSYS (Table 2). Among LL individuals (excluding German *P. lessonae* due to missing data) and RR individuals, 15 and 91 species-specific MLGs were identified, respectively. In RR individuals, 54 MLGs occurred in all-R populations and 37 in R-E populations, with seven shared between these two population types. In diploid *P. esculentus* (LR), 37 MLGs were detected, of which 13 were associated with the L-E system, 18 with the R-E system, and 8 with all-hybrid populations. Only two LR-specific MLGs were shared between R-E and all-hybrid populations (Supplementary Material 6).

**Fig. 6:**
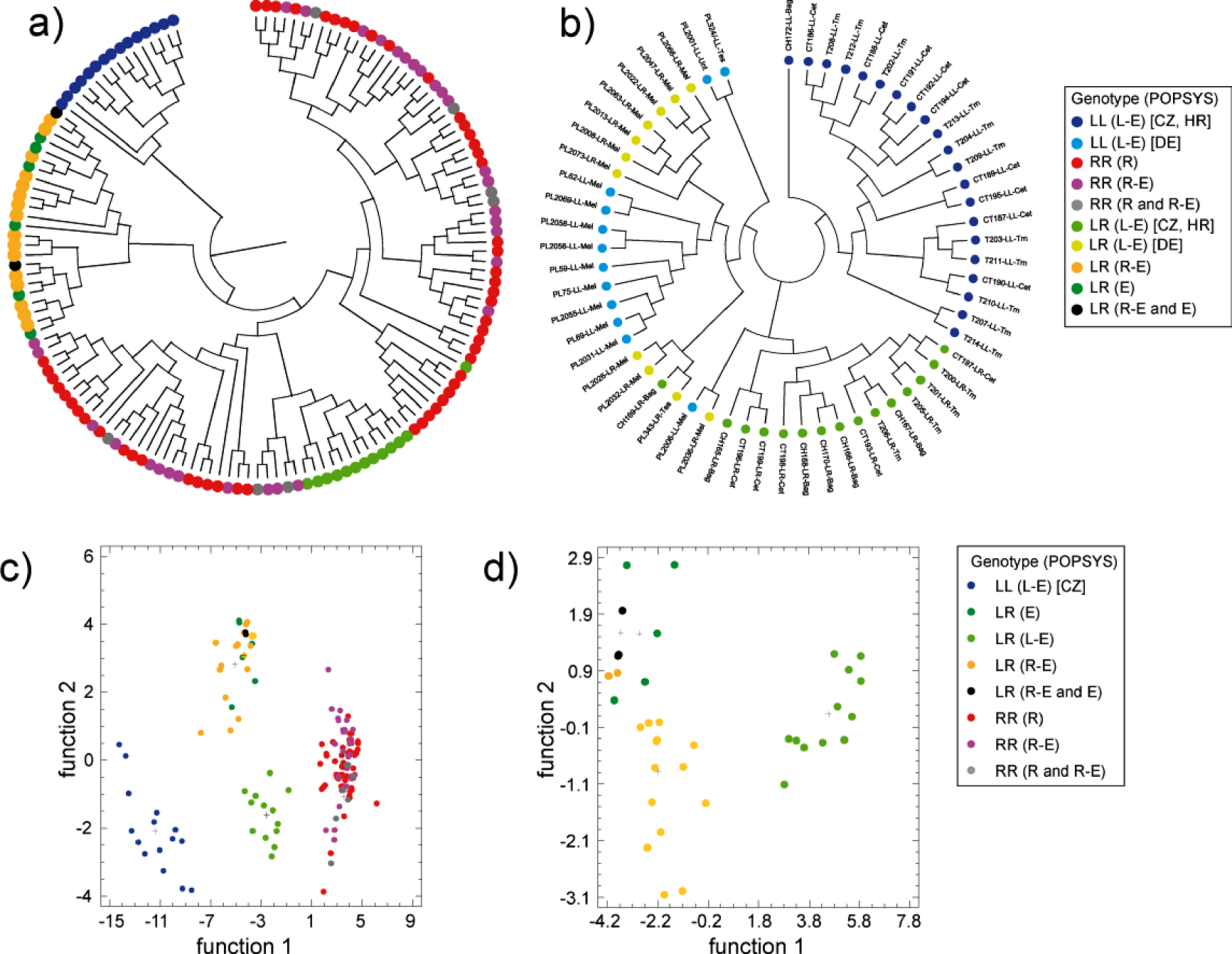
UPGMA clustering (**a**, **b**) and discriminant analysis (**c, d**) of multilocus genotypes (MLGs) based on the SNPs presented in Fig. 4. The results demonstrate a clear genetic differentiation among *P. lessonae* (LL), *P. ridibundus* (RR), and their diploid hybrid *P. esculentus* (LR). (**a**) UPGMA tree based on 14 SNPs showing a clear separation of LL, RR, and LR genotypes. LR genotypes form two distinct clusters: one comprising individuals from L-E populations and the other from R-E and all-hybrid populations. (**b**) UPGMA tree including only individuals from the L-E system, based on 13 SNPs (due to missing data). The tree structure reveals clear differences between German and Czech/Croatian individuals (both LL and LR) within the L-E system. (**c**) Discriminant analysis confirms the genetic differentiation among LL, RR, and LR genotypes, as well as between LR hybrids from diploid L-E populations and those from R-E and all-hybrid populations. In contrast, RR genotypes could not be differentiated according to the population system (all-R vs. R-E). (**d**) Discriminant analysis, including only diploid hybrids (LR), showing genetic differentiation among population systems, but higher similarity between the R-E system and all-hybrid populations compared to the L-E system. Country abbreviations: CZ – Czech Republic; HR – Croatia; DE – Germany.

Consistent with the observed SNP frequencies (Fig. 4), LL-, RR-, and LR-specific MLGs were clearly discriminated by UPGMA clustering and nucleotide-based discriminant analysis (Fig. 6). With respect to population systems, RR-specific MLGs from all-R and R-E populations formed a single cluster, as did LR MLGs from R-E and all-hybrid populations. Furthermore, MLGs identified in the German L-E populations were distinct from those in the Czech and Croatian L-E populations, in which triploid genotypes are absent.

. When all genotypes were considered (Fig. 6c), the stepwise (forward) selection algorithm identified seven SNPs (23, 24, 31, 47, 81, 113, 118) as significant explanatory variables for POPSYS. Of the 143 observed MLGs included in the model, 67.1% were correctly classified according to POPSYS. Among RR individuals, approximately half of the MLGs observed in all-R populations were assigned to the R-E system, whereas 37.8% of MLGs from the R-E system were assigned to the all-R populations. For LR genotypes, 33.3% of the MLGs from the R-E system were misclassified as belonging to all-hybrid populations, and 12.5% of MLGs from all-hybrid populations were assigned to the R-E system. In contrast, all MLGs of the L-E system were correctly classified.

A similar pattern emerged when only LR MLGs were analyzed (Fig. 6d). Based on four significant predictive SNPs (31, 47, 81, 113), classification accuracy improved to 84.6%. Again, all MLGs from L-E populations were correctly classified, whereas misclassification rates of 27.8% and 12.5% were observed for MLGs from the R-E system and the all-hybrid populations, respectively. Some of these SNPs were not randomly associated, i.e., they were in linkage disequilibrium, with distinct patterns observed in *P. lessonae* and *P. ridibundus* (Table S10, Supplementary Material 1).

In summary, analysis of the SNP data revealed two key findings: (1) LR genotypes from the all-hybrid populations were genetically more similar to LR hybrids from the R-E system than to LR genotypes from the L-E system lacking triploid hybrids; and (2) LL genotypes from L-E populations containing only diploid hybrids (LR) differed from conspecifics of German L-E populations that include both diploid and triploid hybrids.

## Discussion

### Implications for hybridogenetic inheritance

In this study, we characterized for the first time 160 gametogenetic genes of Western Palearctic water frogs (genus *Pelophylax*). Analysis of SNP data from diploid hybrids (LR) revealed ten genes associated with specific population systems (L–E, R–E, all-hybrid), making them potential candidates for involvement in premeiotic genome exclusion in the germline of hybridogenetic water frog hybrids. Whether these genes are directly involved in meiotic drive or are merely linked to other segregation distorters, such as retroelements (e.g^60–62^), remains an open question.

Our candidate genes participate in diverse molecular processes, such as spindle formation and chromosome movement during mitosis and meiosis, epigenetic modifications (e.g., histone methylation and acetylation), and cell cycle regulation. Among these genes, *kif22* and *nusap1* are particularly noteworthy. *Kif22* encodes a kinesin-like protein (KIF22), which belongs to a family of chromokinesin proteins known to promote chromosome congression and microtubule dynamics during mitotic prometaphase and metaphase in vertebrates, including *Xenopus*^63–65^. During anaphase and telophase, KIF22 contributes to the dense condensation of the chromosomes, ensuring their proper enclosure by renewing the nuclear envelope. This process is critical for the correct segregation of chromosomes into daughter cells, as demonstrated in mice^66,67^. *Kif22* is also thought to play a role in regulating spindle length, allowing the chromosomes to assemble correctly^68,69^.

*Nusap1*, which encodes a nucleolar and spindle-associated protein that enhances the association between KIF22 and microtubules^70^, is also involved in mitotic spindle organization, chromosome movement, and assembly by bundling microtubules and connecting them to chromosomes^71–73^. In addition, *nusap1* regulates *kif22*, promoting its function in generating the polar ejection force and in this way, controlling the oscillation of chromosomes during metaphase, which is crucial for their correct alignment^70^. The close functional interplay between *kif22* and *nusap1* is consistent with their strong linkage disequilibrium, as observed in both *P*. *lessonae* and *P*. *ridibundus*. The role of *kif22* and *nusap1* in clonal inheritance and their contribution to irregularities observed in hybrid gonocytes, such as unaligned metaphase chromosomes, high proportions of aneuploid cells^74,75^, or meiotic aberrations in male hybrids^76^, remains to be investigated.

Other genes associated with the population system are *hormad1*, *rad50*,* sfr1*, and *henmt1. Hormad1* plays a critical role in early recombination events during meiosis by ensuring the formation of double-strand breaks (DSBs), which are essential for homology search and chromosome synapsis^77–80^. Conversely, *rad50*, *rad51ap1*, and *sfr1* are central to DSB repair via homologous recombination^82–88^. An association between DSBs and genome exclusion was also suggested by the results of a preliminary study on germinal nuclei (stages 31–33^46^ using epigenetic markers^89^; while an increased occurrence of DSBs was indicated in *P. esculentus* larvae, no evidence for DSBs was found in larvae of the parental species (LL, RR). DSBs are caused by various internal and external factors, including transposition events^90,91^. In this context, transposable elements (TEs) may also play an important role in genome elimination, as these elements can induce DSBs. When different TE populations are combined, as in interspecific hybrids, disruptive interactions between these elements are likely to occur during meiotic recombination and DNA repair processes, leading to genomic alterations such as deletions, duplications, and large-scale rearrangements, including the emergence of new chromosome forms^90–92^.

A potential role of TEs in genome exclusion is consistent with their genome-specific deregulation during epigenetic reprogramming (demethylation) in primordial germ cells (reviewed by^93^. Transposable elements are silenced by various mechanisms to avoid uncontrolled activity and maintain genomic integrity (reviewed by^94^. One of the genes involved in TE silencing, *henmt1*, ensures the abundance and functionality of PIWI-interacting RNAs (piRNAs)^95–99^, which are crucial for protecting the germline genome against TEs (e.g.^100^). Disruption of *henmt1* impairs piRNA production^97,101^, leading to fertility issues in mammals and oocyte defects in zebrafish^97,99,102^. Furthermore, TEs can influence centromere evolution, potentially altering both structural specificity and function^103–105^. In this context, centromeric differences arising from TE-derived tandem repeats, such as the *Rr*S1 sequences of Western Palearctic water frogs^106–109^, may contribute to biased, non-Mendelian chromosome segregation^110–112^.

Regardless of the specific genetic factors and molecular processes underlying genome exclusion, non-Mendelian inheritance is invariably associated with hybridization (e.g.^9^). According to the ‘balance hypothesis’ first proposed by Moritz et al.^113^, clonal reproduction is favored when hybridization creates sufficient genomic imbalance to interfere with normal gametogenesis, yet not so severe as to impair hybrid viability. This framework has often been invoked to explain the origin of unisexual or hybridogenetic vertebrates and highlights the delicate balance between genetic divergence and developmental stability required for such lineages to persist.

Considering the phylogenetic relationships of Western Palearctic waterfrogs^114^, the *Pelophylax* system does not conform to the balance hypothesis because hybrids of *P. lessonae* and *P. ridibundus* are almost exclusively hybridogenetic, whereas genome exclusion is absent in hybrids between *P. lessonae* and *P. kurtmuelleri*—the sister taxon of *P. ridibundus*, with a degree of genetic divergence from *P. lessonae* comparable to that of *P. ridibundus*. Likewise, *P. grafi* (a hybrid of *P. ridibundus* and the distantly related *P. perezi*) reproduces hybridogenetically, whereas hybrids between Central European *P. ridibundus* and *P. shqipericus* (a species closely related to *P. lessonae*) are not hybridogenetic^50^. These findings indicate that hybridogenetic reproduction is not primarily determined by overall genomic divergence, but by complex interactions among multiple genome-specific factors^115^, rather than being caused by a single R-specific driving factor as proposed by Uzzel et al.^49^ and Hotz et al.^50^.

### Recombination and gene flow as a source of genome diversification

In addition to novel mutations, gene flow has likely contributed substantially to the diversification of the L and R genomes, as indicated by both transcriptomic and SNP data. SNP-based estimates of introgression - approximately 1% per population in RR and 0.3% in LL – are consistent with previous studies suggesting predominantly unidirectional gene flow from *P. lessonae* to *P. ridibundus*^52,116–120^. The close similarity between specific R alleles and their L orthologs further supports this interpretation. However, alternative explanations cannot be excluded. Apparent L-specific SNPs in RR individuals may also reflect introgressive hybridization between *P. ridibundus* and *P*. *kurtmuelleri*, assuming that *P. kurtmuelleri* and *P. lessonae* share identical nucleotides at a specific site. Alternatively, independent identical substitutions, particularly at synonymous or degenerate sites, could contribute to the observed patterns.

Genetic recombination during hybrid gametogenesis is a precondition for gene flow between *P. lessona*e and *P. ridibundus*. Recombined genomes can re-enter the parental gene pools either through backcrosses between hybrids and parental individuals or through homotypic hybrid crosses, provided that the mating hybrids produce the same type of gametes and the resulting non-hybrid (parental) offspring are viable^56,121,122^. However, in most cases, parental genotypes (LL, RR) arising via hybridolysis^121^ are nonviable and/or die before reaching sexual maturity^123–128^. This phenomenon is thought to be caused by homozygosity for recessive deleterious alleles that accumulate in clonally inherited genomes, a process known as Muller’s ratchet^129^. Occasional genetic recombination^38,43^ can mitigate the effects of deleterious alleles^122^. Conversely, recombined but unbalanced genomes may cause developmental defects and fertility disorders, which are frequently observed in *P. esculentus*^124,130–132^.

Genetic recombination and bidirectional introgression mediated by migration may have played an important role in the emergence of different non-Mendelian inheritance modes, and thus, in the evolution of distinct population systems. The observed differences between the L genomes of all-diploid and mixed-ploidy L-E populations, together with the high genetic similarity of LR genotypes from all-hybrid and R-E populations, suggest that the L genome, alongside the R genome, may contribute significantly to the hybridogenetic reproduction of *P. esculentus*. This hypothesis is further supported by the high similarity of L genomes in R-E populations inhabiting the Oder Valley, where many LR hybrids clonally inherit the L genome or both the R and L genomes^55,56^. Whether these L genomes are similar or even identical to clonally inherited L genomes from mixed-ploidy L-E and/or all-hybrid populations remains to be elucidated.

## Conclusions

The association of specific gametogenetic genes with distinct population systems (L-E, R-E, all-hybrid) suggests that hybridogenetic gametogenesis is controlled by a complex network of interacting genes and regulatory factors that are involved in diverse molecular processes such as germ cell differentiation, spindle formation, chromosome segregation during mitosis and meiosis, DSB repair, homologous recombination, synaptonemal complex formation, and epigenetic regulation. Whether these genes directly mediate genome exclusion or are instead associated with specific segregation distorters remains to be determined.

Both transcriptomic and SNP data provide new evidence for genetic recombination and gene flow in water frogs as sources of genetic variability. Occasional recombination events, acting in concert with stabilizing selection, may have driven substantial modifications in gene regulatory networks, contributing to the evolution of different modes of clonal inheritance (i.e., exclusion of the L and/or the R genome) in hybridogenetic hybrids^37^, as well as to the frequently observed fertility and developmental disorders^45,121,131^.

Our genomic data provide a solid foundation for future experimental investigations of germline-specific regulatory mechanisms underlying genome exclusion and clonal gamete formation in European water frogs. Artificial crosses combining genomes from individuals of different genotypes, geographic origins, and population systems will be essential for identifying causal links between specific genetic markers and hybridogenetic inheritance patterns.

## Materials and methods

### De novo sequencing and assembly of *Pelophylax* transcriptomes

Tissue samples (testes) were collected from one *P. lessonae* male (genotype LL, field number PL74) collected near Melzow, Germany (53°11’00”N, 13°54’00”E), and from three *P. ridibundus* males: two (RR1, PL27 and RR2, PL32) from fish ponds east of Mělník, Czech Republic (50°21’54.33”N, 14°26’45.89”E), and one (RR3, PL88) from the Oder river near Frankfurt, Germany (52°24’49.77”N, 14°32’32.65”E). Animals were euthanized in a buffered solution (pH 7) of 3-aminobenzoic acid ethyl ester (MS-222, Sigma, St. Gallen, Switzerland). Tissues were snap- frozen in liquid nitrogen and stored at − 80 °C. RNA and DNA were simultaneously extracted using the AllPrep DNA/RNA Mini Kit (Qiagen, Hilden, Germany). The frozen tissues were ground in liquid nitrogen with a mortar and pestle and further homogenized in RLT buffer using a TissueLyser (2 min at 20 Hz). RNA quantity and integrity were assessed with a Qubit^®^ 2.0 Fluorometer (Life Technologies, Carlsbad, USA) or a BioAnalyser 2100 (Agilent Technologies, Santa Clara, California, USA) according to the manufacturer’s instructions.

Organ-specific mRNA-Seq libraries were prepared from 2 µg of total RNA using the TruSeq RNA Sample Prep Kit v2 (Illumina, San Diego, CA, USA), following a modified protocol designed to preserve directional information about the transcripts^133^. First, mRNA was isolated from the total RNA pool and chemically fragmented, followed by double-stranded (ds) cDNA synthesis incorporating dUTP into the second strand to enable strand specificity. Then, standard Illumina sequencing library preparation steps were performed, including end polishing, A-tailing, adapter ligation, and size selection. The dUTP-labeled strand was selectively degraded using uracil-DNA-glycosylase (UDG), leaving the complementary strand to be amplified for cDNA library generation. Sequencing was performed on an Illumina HiSeq 2000 platform (2 × 50 bp paired-end reads), producing an average of 50 million read pairs, corresponding to approximately 2.5 Gb of data per tissue sample^134^.

Different pipelines were established for quality control, primarily based on FASTQ manipulation and trimming tools, including Fastx (http://hannonlab.cshl.edu/fastx_toolkit/), Trimmomatic^135^, and Sickle^136^. Kraken was used to remove sequence contaminants from Illumina reads. Assemblies were generated using SOAPdenovo-Trans (release 1.03, 07-25-2013^137^ and Trinity 2.2.0^138^. For SOAPdenovo-Trans, parameters were modified from the defaults to include -K31, -F, -R, and -t 1. Prior to transcriptome reconstruction with Trinity, technical sequences were removed with Trimmomatic v0.36^135^. The filtered reads were then concatenated and assembled with Trinity under default settings, enabling the read normalization option.

In addition to conventional assembly statistics, such as N50 (the minimum contig length required to cover 50% of the assembly), transcriptome completeness was evaluated by counting the number of ultra-conserved eukaryotic core proteins present in each assembly. Protein sequences were predicted from the transcriptome data using ESTScan (http://estscan.sourceforge.net/) and subsequently employed for annotation and downstream analysis. All transcriptome assemblies are available on our in-house BLAST server (blastserver.genomica-australis.de).

### Sequencing and de novo assembly of *Pelophylax* genomes

Genomic DNA of *P. lessonae* was isolated from the male PL74 using the Qiagen MinElute system (Qiagen). DNA concentration was quantified with a Qubit^®^ 2.0 fluorometer. Standard paired-end DNA sequencing libraries with average insert sizes of approximately 250 bp and 1 kb were prepared from 1 µg of DNA using the TruSeq DNA Sample Preparation Kit v2 (Illumina). Five lanes of 101 bp paired-end reads were sequenced on an Illumina HiSeq 2000 system, yielding approximately 170 Gb and 15 Gb of data, respectively. K-mer frequency analysis performed with KmerFreq AR v2.0 of the SOAPdenovo2 package^139^, estimated a genome size of approximately 7 Gb, corresponding to a paired-end read coverage of ~ 26x.

Three Illumina Mate Pair libraries were generated using the Nextera system (Illumina) with experimental insert sizes of 2.5, 5, and 10 kb, sequenced to genome coverages of ~ 10x, ~ 10x, and ~ 2.5x, respectively. After quality filtering, removal of PCR duplicates, adapter trimming, and error correction using SOAPfilter_v2.0 with default settings, (github.com/tanghaibao/jcvi-bin/blob/master/SOAP/SOAPfilter_v2.0) a total of 2.6 billion reads (~ 260 Gb) were retained, corresponding to an overall genome coverage of ~ 36x.

For the R genome, the same types of libraries were prepared as described above, using DNA from the male PL88. Fragment libraries with insert sizes of 260 bp and 1 kb were sequenced at 2.5x, 1.9x, and 2.3x coverage. In total, 955 million reads (~ 100 Gb) were sequenced, corresponding to ~ 14x coverage.

The L genome assembly was generated from 1.7 billion reads derived from five libraries with insert sizes of 250 bp, 1 kbp, 2 kbp, 5 kbp, and 10 kbp. The scaffolded genome spanned 4.9 Gb and contained approximately 3.45 million scaffolds. High-quality reads, retained after filtering with SOAPfilter_v2.0, were assembled de novo with SOAPdenovo2_v2.04^139^. Assemblies that incorporated all 2.6 billion reads yielded a total assembled genome size of 6.3 Gb and an N50 value of 136,366 bp (assembly LL-2, Table S11, Supplementary Material 1); with up to 40% of sequences being ambiguous. Subsequent analysis revealed that the predicted peptide sequences from this assembly were of lower quality and shorter length compared to assemblies generated using only the 1 kbp and 2.5 kbp fragment libraries (assembly LL-1, Table S11, Supplementary Material 1).

The selected L genome assembly was generated as follows: a K-mer graph was constructed through Sparse-pregraph with parameters -K 49 -g 15 -z 10 9 -d 1 -e 1 -R -r 0 -p 50. Contigs were computed with the default settings except for – R and -p50. The remapping step of SOAPdenovo was conducted using default settings, and scaffolding was performed using parameters -F -G 200 -p 60. Remaining gaps were closed via SOAP Gapcloser v1.12. Gene predictions were performed with GeneScan^140^, employing the human matrix, resulting in 380 Mb of predicted protein-coding sequence.

The R genome assembly was generated from 955 million reads using default settings and parameters -K49, -R, -F, -G 200. Due to insufficient sequence coverage, the assembly was highly fragmented, with over 60% of the sequences containing gaps (Ns). In total, 78 Mb of coding sequences were predicted (Table S11, Supplementary Material 1).

The completeness of both genome assemblies was assessed with CEGMA^141,142^, which identifies ultra-conserved core genes, and the BUSCO pipeline^143^, using the eukaryote lineage reference protein set. Both genome assemblies are accessible via our in-house BLAST server (blastserver.genomica-australis.de).

### Selection and characterization of genes known to be involved in gametogenesis

Genes known to be involved in gametogenesis were obtained from the databases UniProt (www.uniprot.org/, The UniProt Consortium 2017^144^ and KEGG (www.genome.jp/kegg^145^. Reference nucleotide or amino acid sequences of these genes were retrieved from Xenbase (http://www.xenbase.org/entry/^146^, the database for the anuran model species *Xenopus tropicalis*, except for *rec114* and *tex12*, for which *Mus musculus* sequences were obtained from UniProt. These reference sequences were blasted against the LL and RR transcriptomes using TBLASTN^147^. Hits with an E-value < 0.05 were considered homologous, and the highest quality scaffolds were selected for further analysis. Open reading frames (ORF) were identified using ORF-finder (RRID: SCR_016643), available at www.ncbi.nlm.nih.gov/orffinder. In total, 620 transcriptomic sequences representing 160 gametogenic genes were obtained. Gene names are listed in Table S1, Supplementary Material 1, and the corresponding nucleotide sequences are presented in Supplementary Material 2.

Coding sequences (CDS) of the selected genes were manually curated with MEGA (version 7.0.26)^148^ and aligned with ClustalW^149^ or MUSCLE^150^ as implemented in the software. Basic sequence features, including coding sequence length (CDSL) and GC content, were recorded to provide baseline information, facilitate comparison with related genes, and support reproducibility in downstream analyses. Intra- and interspecific genetic divergence was assessed by calculating the number of SNPs and uncorrected p-distances between individual alleles. For incomplete sequences, the percentage of missing nucleotides (Ns) was quantified with BioEdit 7.2.5^151^. Sequences exceeding 5% Ns were excluded from further analysis, which affected the genes *ago3*, *ar*, *dmrt2*, *dmrt5*, and *spata1* (Table S2, Supplementary Material 1). The intron-exon structure of each gene was determined with Splign (bio.tools/splign^57^, based on L*-*specific transcripts and scaffolds from the L and/or R genome drafts.

### Genotyping-in-thousands by sequencing (GT-Seq)

GT-Seq is a high-throughput sequencing method that enables the analysis of multiplexed PCR products targeting 50–500 SNPs (in some cases up to 2,000) across large numbers of individuals in a single Illumina HiSeq lane^152^. The method uses unlabeled oligonucleotides and PCR master mixes in two thermal cycling steps to amplify the target SNP loci. Sequencing adapters and dual barcode sequence tags are incorporated into the amplicons, primarily during the second PCR step. Amplicons from all individuals are pooled into a single sequencing library. After sequencing, reads are demultiplexed into individual files according to unique barcode combinations.

#### Sampling and taxon designation

 A total of 652 water frog samples, collected in previous studies [e.g., 42,56], were analyzed: 60 *P. lessonae*, 252 *P. ridibundus*, and 340 *P. esculentus* from various populations and population systems (Table S6, Supplementary Material 1; Fig. S3, Supplementary Material 4; Supplementary Material 5). Adult individuals were sexed based on sexual characters, such as vocal sacs and nuptial pads, and assigned to taxa (*P*. *lessonae*, *P*. *ridibundus*, *P*. *esculentus*) according to their external morphology (reviewed by^36,153^. After euthanasia with MS-222, tissue samples were collected from toe clips or skeletal muscle and stored at − 20 °C or in 80% ethanol until processing. These tissues were also used for genotyping using various markers, including allozymes^35,154^, microsatellites^42,43,55,155,156^, PCR-based length polymorphisms^157^, and nuclear (Sanger) sequences of the genes *mnd1* and/or *uqcrfs1*^41,158^, as well as read counts derived from GT-Seq data (Fig. S2, Supplementary Material 4).

#### DNA isolation, selection, and amplification of SNP loci

 Genomic DNA from 652 individuals was isolated from blood, muscle, and liver tissues using either NucleoSpin (Macherey-Nagel, Düren, Germany) or DNeasy Blood and Tissue (Qiagen) kits. For some samples, DNA was extracted using the classical phenol-chloroform method^159^. DNA concentration was measured with a Qubit™ fluorometer (Life Technologies) using the Qubit™ dsDNA BR Assay Kit or a NanoDrop spectrometer (Thermo Fisher Scientific, Waltham, USA).

SNP loci were selected from CDS alignments of LL and RR transcripts. First, consensus sequences were generated for each gene-specific alignment using Consensus Maker (www.hiv.lanl.gov/content/sequence/CONSENSUS/SimpCon.html) and then split into exon-specific sequences using Splign^57^. For exonic sequences containing SNP loci, forward and reverse primers were designed using Ion AmpliSeq Designer (Thermo Fisher Scientific, www.ampliseq.com/login/login.action), yielding PCR products of 70–120 bp (140–190 bp including Illumina-specific fusion sequences). All primers included specific 5’ and 3’ overhangs compatible with Illumina sequencing technology to allow for adapter and barcode annealing during PCR.

Primers prone to dimer formation or nonspecific amplification^160^ were removed from primer pools via size-selection purification using magnetic beads. Exon-specific primer pairs were validated by single-template PCRs followed by Sanger sequencing. Validated primers were ordered in a 96-well plate format (Supplementary Material 7), diluted to 100 µM, and combined in various multiplex pools. Primer pools containing 30–50 different primer pairs were prepared at 1 µM per primer to optimize multiplex PCR.

#### GT-Seq library preparation and sequencing

 Template DNA from each sample was amplified in two multiplexed reactions (PCR1) using subsets of target primer pairs with different annealing temperatures. The primer pairs were divided into seven pools (Supplementary Material 7) and distributed across two PCR1 mixes. Each PCR1 mix (10 µl) contained 50–100 ng of extracted DNA, 0.1 µl Taq polymerase (Qiagen), 1 µl 10x PCR buffer (Qiagen), 0.2 µl dNTP mix (10 µM), 0.5 µl primer pools 1–6 or 7–8 (100 µM), and PCR grade water (Qiagen) to reach the final volume. Reactions were performed in 96-well plates under the following thermal conditions: an initial denaturation at 95 °C for 5 min, five cycles of 95 °C for 30 s, ramp down from 95 °C to 57 °C for 30 s (ramp rate: 0.3 °C/s), and 72 °C for 1 min; followed by 10 cycles of 95 °C for 30 s, 60 °C for 30 s, and 72 °C for 1 min, and finally a hold at 15 °C.

Following PCR1, the two multiplex PCR reactions of each sample were diluted 1:20. One microliter of each diluted product was used in a second amplification step (PCR2). The 10 µl PCR2 mix contained 1 µl template DNA, 0.1 µl Taq polymerase (Qiagen), 1 µl 10x PCR buffer (Qiagen), 2 µl i5/i7 tagging primer mix (1 µM), 0.2 µl dNTP mix (10 µM), and PCR-grade water to reach the final volume of 10 µl. PCR2 cycling conditions consisted of an initial denaturation at 95 °C for 5 min; five cycles of 95 °C for 30 s, 60 °C for 30 s, and 72 °C for 1 min; ten cycles of 95 °C for 30 s, 72 °C for 30 s, and 72 °C for 1 min; followed by a final extension at 72 °C for 5 min, and a hold at 15 °C.

The products from multiplex PCRs were normalized using a SequalPrep™ Normalization Plate Kit (Applied Biosystems) according to the manufacturer’s instructions. After normalization, the multiplex reactions for each 96-well plate were transferred to a new plate, and 2 µl of each sample was pooled, resulting in seven pools containing 96 samples each. DNA concentration was quantified using a Qubit™ dsDNA BR Assay Kit, and DNA quality was assessed with a Fragment Analyzer (Agilent Technologies).

All seven pools were purified by mixing 165 µl of each pool with 250 µl of Agencourt^®^ AMPure XP magnetic beads in new low-binding PCR tubes, followed by incubation at room temperature for 15 min. The beads were separated from the remaining liquid using a magnetic rack. After removing the supernatant, the beads were washed twice with 80% ethanol and air-dried for 15 min. The semi-dried beads were resuspended in 30 µl of Illumina RS buffer, and DNA was allowed to dissolve from the beads for 5 min at room temperature. The tubes were then placed back on the magnetic rack, and the supernatant containing purified DNA was transferred to new tubes. DNA concentration and library quality were subsequently assessed using a Qubit™ dsDNA BR Assay Kit and a Fragment Analyser (Agilent Technologies), respectively.

The quality of the libraries and the evenness of target sequence coverage were verified by sequencing a small aliquot of each partial library (containing 96 samples) on an Illumina MiSeq™ platform. The final library, comprising 672 samples from 652 individuals, was sequenced on an Illumina NextSeq550 using a high-output single-end 75 bp sequencing kit (NextSeq 500/550 High Output Kit v2.5 [75 cycles], PN 20024906) with dual index reads of 2 × 8 bp. Following sequencing, bcl files were demultiplexed and converted to FASTQ format.

#### Data processing

GT-Seq output files were processed as compressed FASTQ files (fastq.gz) containing both sequence reads and corresponding quality scores. For initial visualization and quality control, the FASTQ files, along with the reference and primer sequences (both in FASTA format), were analyzed using Geneious 11.1.5. (www.geneious.com). Primers and residual homopolymer sequences were trimmed from the reads, resulting in sequences with a maximum length of 70 bp. The trimmed reads were then mapped to reference sequences (templates) containing the marker-specific sequence motifs. Individual motifs were separated by approximately 200-base-long artificial spacer sequences composed of combined adenine (A) and thymine (T) repeats (Supplementary Material 8). The resulting sequence assemblies were visually inspected and compared to transcriptome-based alignments, with particular attention to sequence homology and the presence of the selected SNPs.

Subsequently, all reads were mapped with BWA-0.7.17^161^ to both LL- and RR-specific reference sequences (Supplementary Material 8), which had been manually curated based on available water frog transcriptomes. Unlike the approach applied in Geneious, only residual homopolymer regions were trimmed from the reads, while primer sequences were retained, as their inclusion improved mapping accuracy and efficiency. Extensive testing with different mapping parameters was performed to ensure retention of all SNP alleles identified from transcriptome comparisons. The resulting assemblies were sorted and indexed using SAMtools-1.8^162^. The mapped and sorted reads were converted into pileup files summarizing base calls (A, T, C, G) and coverage for each position through a custom analysis pipeline. Merging, simplifying, and splitting of multiple pileup files was performed via a custom Python script, resulting in consolidated final pileup files summarizing all markers and individuals analyzed.

The pileup files were subsequently processed with an in-house script to convert the data to FASTA format and generate a dataset containing a consensus sequence for each individual, incorporating all SNP markers. The minimum total coverage threshold (COV_tot_) per polymorphic site (SNP) was set at 10 reads. Nucleotides were considered heterozygous when their minimum relative coverage (COV_min_) reached at least 20% of COV_tot_ at a given site. Lowering COV_min_ increased the risk of false-positive heterozygotes, whereas raising it could result in false-positive homozygotes – particularly in triploid hybrids (LLR, RRL) – as demonstrated by a trial using a COV_min_ of 30%.

The resulting FASTA files were examined in MEGA7^148^, and polymorphic sites were extracted and compiled into a new FASTA file for downstream analyses. After critical quality assessment, 131 SNPs representing 52 gametogenic genes were retained for further analysis (Table S5, Supplementary Material 1).

### Statistical analysis

#### SNP allele frequencies and genetic distance

Each extracted SNP was assigned to its specific position in the CDS. To minimize potential bias, SNPs present in fewer than five individuals per taxon (parental species or hybrids) were exclu-ded from the dataset. In addition, populations with a sample size < 4 per genotype were pooled if they belonged to the same population system (POPSYS) and the same geographical region (e.g., LL genotypes from the German L-E populations Melzower forst and Teschendorf; RR genotypes from the Bulgarian all-R populations Eleshnitsa and Brodilovo). The only exception was for diploid hybrids from Borovec, a population with unclear system assignment (suggested to be an R-E system), for which allele frequencies were calculated in two ways: (i) for all hybrids combined, and (ii) for male and female hybrids separately, as females from the neighboring L-E system population Trnávka may have migrated to the R-E system of the Oder river, which is typically free of hybrid females^52^. After this optimization, each genotype- and population-specific data set contained 131 SNPs, which served as the basis for allele frequency calculations. Alleles occurring with frequencies ≥ 0.95 in only one of the parental species were considered species-specific. Triploid hybrids (LLR, RRL) were excluded from further analyses due to their disproportionate number of homozygous loci, likely resulting from methodological errors caused by dosage effects in molecular reactions

### Selection of genes associated with population systems

Logistic regression models were computed with STATGRAPHICS centurion 18.1.08 (Statgraphics Technologies, Inc., the Plains, Virginia) to identify SNPs significantly associated with POPSYS, thereby detecting genes potentially linked to the system-specific inheritance patterns. Logistic regression also facilitated the distinction between system-inherent (genome-linked) and geographical effects.

Because the input variables were frequencies rather than binary variables, logistic regression was performed using weighted least squares, as recommended for such datasets. Initially, all predictor variables were included in the model, and only first-order (main) effects were analyzed. Stepwise selection was subsequently applied to identify a parsimonious model that retained only statistically significant variables. The procedure calculates t-statistics with corresponding *p*-values for each coefficient and evaluates the statistical significance of the entire model using ANOVA. Additionally, it calculates a standard R² statistic, tests the effects of the prediction factors via F tests based on type III sums of squares, and conducts a chi-squared test to assess whether the fitted model adequately describes the observed data by dividing the fitted logit values into classes and comparing the observed and fitted values in each interval. Logistic regressions were performed separately for the LR and RR datasets, but not for the LL dataset due to insufficient data.

### Construction and analysis of multilocus genotypes

Individual multilocus genotypes (MLGs) were constructed as FASTA files using SNPs whose nucleotide frequencies were significantly associated with POPSYS (Table 2; Tables S8–S9, Supplementary Material 1). Based on these MLGs, a distance matrix (uncorrected p-distances) was calculated using splitstree v. 4.16.2^163^. In this approach, ambiguous sites were treated as “average states”, with contributions at a site averaged across all possible resolutions of the ambiguous codes, while sites with identical ambiguous codes contributed zero. In addition, MLGs were converted into numerical sequences following Holden et al.^164^ by assigning each nucleotide an atomic number, i.e., the total number of protons (A = 70, T = 66, C = 58, G = 78). These values, which are proportional to the nucleotide mass (excluding isotopes), were used as quantitative variables (input data) in a discriminant analysis (implemented in STATGRAPHICS), with the MLGs serving as the classification factor. The objective of this procedure was to mathematically describe the observed cases in a way that separates them into groups by constructing linear combinations of the input variables. The analysis included all variables and employed a stepwise selection procedure to identify only those variables that were statistically significant discriminators between groups. For the stepwise regressions, the F-value threshold was set at 4.0, with a maximum of 50 steps allowed.

## Supplementary Information

Below is the link to the electronic supplementary material.

Supplementary Material 1

Supplementary Material 2

Supplementary Material 3

Supplementary Material 4

Supplementary Material 5

Supplementary Material 6

Supplementary Material 7

Supplementary Material 8

## Data Availability

Sequence data generated for this project are available from EMBL under the accession number PRJEB85627. All other data presented in this study are included in the article and Supplementary Material. For further inquiries, contact the corresponding author.
