# Supplementary material for: New insights into the molecular basis of gametogenesis in the hybridogenetic water frog Pelophylax esculentus
Source: Sci Rep. 2026 Feb 4;16:5012. doi: 10.1038/s41598-026-37515-w (PMC12876976; doi:10.1038/s41598-026-37515-w)
Supplement: Supplementary file 5 — Supplementary Material 5 [file 41598_2026_37515_MOESM5_ESM.docx]

**Plötner et al. 2026_Supplementary Material 1 (Tables S1–S11)**

**Table S1**. Gametogenic genes with full names and GenBank accession numbers of their *Xenopus tropicalis* or *Mus musculus* orthologs (marked with an asterisk).

| **Gene** | **Full name** | **Accession number** |
| --- | --- | --- |
| ***adarb1*** | Double-stranded RNA-specific editase 1 | XM_018097020.1 |
| ***adcy9*** | Adenylate cyclase type 9 | XM_002932430.4 |
| ***ago2*** | Argonaute 2, RISC catalytic component | XM_012964517.2 |
| ***ago3*** | Argonaute 3, RISC catalytic component | XM_002936224.4 |
| ***ago4*** | Argonaute 4, RISC catalytic component | XM_004911574.3 |
| ***akt1*** | RAC-alpha serine/threonine-protein kinase | XM_002940717.4 |
| ***anapc13*** | Anaphase promoting complex subunit 13 | NP_001017240 |
| ***ar*** | Androgen receptor | XM_002941842.4 |
| ***asz1*** | Ankyrin repeat, SAM and basic leucine zipper domain-containing protein 1 | XM_012958624.2 |
| ***atr*** | Serine/threonine-protein kinase ATR | XM_012963312.2 |
| ***aurka*** | Aurora kinase A | NM_001016570.2 |
| ***bcar1*** | Breast cancer anti-estrogen resistance protein 1 | XM_012960799.2 |
| ***bcar3*** | Breast cancer anti-estrogen resistance protein 3 | NM_001097265.1 |
| ***birc5*** | Baculoviral IAP repeat-containing protein 5 | NM_001044454.1 |
| ***brd2*** | Bromodomain testis-specific protein | NM_001134810.1 |
| ***btrc*** | F-box/WD repeat-containing protein 1A | XM_012966589.2 |
| ***bub1*** | Mitotic checkpoint serine/threonine-protein kinase BUB1 | NM_001079362.1 |
| ***bub3*** | Mitotic checkpoint protein BUB3 | BC079934.1 |
| ***calm*** | Calmodulin | NM_001008159.1 |
| ***camk2g*** | Calcium/calmodulin-dependent protein kinase type II subunit gamma | XM_012966016.2 |
| ***ccna1*** | Cyclin-A1 | NM_001016239.1 |
| ***ccnb1*** | G2/mitotic-specific cyclin-B1 | NM_203790.1 |
| ***ccnb1ip1*** | E3 ubiquitin-protein ligase CCNB1IP1 | XM_018089685.1 |
| ***ccnb2*** | G2/mitotic-specific cyclin-B2 | NM_001145376.2 |
| ***ccnb3*** | G2/mitotic-specific cyclin-B3 | XM_002939199.4 |
| ***ccne2*** | G1/S-specific cyclin-E2 | NM_001016267.2 |
| ***cdc20*** | Cell division cycle protein 20 homolog | NM_203614.1 |
| ***cdc25c*** | M-phase inducer phosphatase 3 | XM_018091902.1 |
| ***cdk1*** | Cyclin-dependent kinase 1 | NM_001145376.2 |
| ***cdk2*** | Cyclin-dependent kinase 2 | NM_001008135.1 |
| ***cdk7*** | Cyclin-dependent kinase 7 | NM_001017219.2 |
| ***cpeb4*** | Cytoplasmic polyadenylation element-binding protein 4 | XM_002936699.4 |
| ***ctcf*** | Transcriptional repressor CTCF | NM_001122796.1 |
| ***cyp26b1*** | Cytochrome P450 26B1 | NM_001079187.2 |
| ***dazl*** | Deleted in azoospermia-like | NM_203748.1 |
| ***ddx19*** | ATP-dependent RNA helicase DDX19 | CR760667.2 |
| ***ddx25*** | ATP-dependent RNA helicase DDX25 | NM_001016345.2 |
| ***ddx4*** | Probable ATP-dependent RNA helicase DDX4 | XM_018093500.1 |
| ***dmc1*** | Meiotic recombination protein DMC1/LIM15 homolog | XM_018093651.1 |
| ***dmrt1*** | Doublesex and mab-3 related transcription factor 1 | XM_018089347.1 |
| ***dmrt2*** | Doublesex and mab-3 related transcription factor 2 | NM_001100256.1 |
| ***dmrt5*** | Doublesex and mab-3 related transcription factor 5 | HQ171197.1 |
| ***dnmt1*** | DNA (cytosine-5)-methyltransferase 1 | XM_002934338.4 |
| ***dnmt3a*** | DNA (cytosine-5)-methyltransferase 3A | XM_004919615.3 |
| ***dusp1*** | Dual specificity protein phosphatase 1 | NM_001005450.2 |
| ***eif4g3*** | Eukaryotic translation initiation factor 4 gamma 3 | NM_001142072.1 |
| ***eme1*** | Crossover junction endonuclease EME1 | NM_001078831.1 |
| ***eme2*** | Probable crossover junction endonuclease EME2 | NM_001079392.1 |
| ***exd1*** | piRNA biogenesis protein EXD1 | XM_004917310.3 |
| ***fbxo43*** | F-box only protein 43 | XM_004915140.3 |
| ***fbxo5*** | F-box only protein 5 | XM_012963092.1 |
| ***fgf9*** | Fibroblast growth factor 9 | XM_002938575.4 |
| ***fkbp6*** | Inactive peptidyl-prolyl cis-trans isomerase FKBP6 | XM_018091140.1 |
| ***foxc1*** | Forkhead box protein C1 | NM_001007863.1 |
| ***grb2*** | Growth factor receptor-bound protein 2 | NM_001008129.1 |
| ***h3.3*** | Histone H3.3 | NM_001005101.1 |
| ***hat1*** | Histone acetyltransferase type B catalytic subunit | XM_012970071.2 |
| ***hdac1*** | Histone deacetylase 1 | NM_001030393.1 |
| ***hec1*** | Kinetochore protein NDC80 homolog | NM_001011162.1 |
| ***henmt1*** | Small RNA 2'-O-methyltransferase | XM_012960696.2 |
| ***hormad1*** | HORMA domain-containing protein 1 | XM_002935467.2 |
| ***igf1*** | Insulin-like growth factor 1 | XM_002936829.4 |
| ***igf1r*** | Insulin-like growth factor 1 receptor | XM_002933305.4 |
| ***ints13*** | Integrator complex subunit 13 | NM_001004984.1 |
| ***itpr1*** | Inositol 1,4,5-trisphosphate receptor type 1 | XM_012961730.2 |
| ***kif22*** | Kinesin-like protein KIF22 | XM_012954607.2 |
| ***kmt2a*** | Histone-lysine N-methyltransferase 2A | XM_018096068.1 |
| ***m1ap*** | Meiosis 1 arrest protein | NM_001130377.1 |
| ***mad2l1*** | Mitotic spindle assembly checkpoint protein MAD2A | NM_001103058.1 |
| ***mael*** | Protein maelstrom homolog | NM_001079177.1 |
| ***map2k1*** | Dual specificity mitogen-activated protein kinase kinase 1 | NM_001008057.1 |
| ***map3k7*** | Mitogen-activated protein kinase kinase kinase 7 | XM_018093745.1 |
| ***mapk1*** | Mitogen-activated protein kinase 1 | NM_001017127.2 |
| ***mapk12*** | Mitogen-activated protein kinase 12 | XM_018092105.1 |
| ***marf1*** | Meiosis regulator and mRNA stability factor 1 | NM_001126066.1 |
| ***mdc1*** | Mediator of DNA damage checkpoint protein 1 | XM_012969222.2 |
| ***mei4*** | Meiosis-specific protein MEI4 | XM_018094012.1 |
| ***meig1*** | Meiosis-expressed gene 1 protein homolog | NM_001016522.2 |
| ***meiob*** | Meiosis-specific with OB domain-containing protein | XM_012970697.2 |
| ***mlh1*** | DNA mismatch repair protein Mlh1 | XM_002933409.3 |
| ***mlh3*** | DNA mismatch repair protein Mlh3 | NM_001251842.2 |
| ***mnd1*** | Meiotic nuclear division protein 1 homolog | XM_012961773.1 |
| ***mns1*** | Meiosis-specific nuclear structural protein 1 | NM_001103012.2 |
| ***mov10l1*** | RNA helicase Mov10l1 | NM_001079156.1 |
| ***mre11*** | Double-strand break repair protein MRE11 | XM_012956402.2 |
| ***msh2*** | DNA mismatch repair protein Msh2 | XM_002935381.4 |
| ***msh4*** | MutS protein homolog 4 | XM_018093532.1 |
| ***msh5*** | MutS protein homolog 5 | XM_018096397.1 |
| ***msh6*** | DNA mismatch repair protein Msh6 | XM_002935375.3 |
| ***msx1*** | Homeobox protein MSX-1 | NM_001037252.1 |
| ***mus81*** | Crossover junction endonuclease MUS81 | NM_001011004.1 |
| ***mybl1*** | Myb-related protein A | XM_002935319.4 |
| ***nbn*** | Nibrin | XM_018095158.1 |
| ***nusap1*** | Nucleolar and spindle-associated protein 1 | XM_012968505.2 |
| ***parn*** | Poly(A)-specific ribonuclease PARN | XM_012970508.2 |
| ***pelp1*** | Proline-, glutamic acid- and leucine-rich protein 1 | XM_012953417.2 |
| ***piwil2*** | Piwi-like protein 2 | XM_018091922.1 |
| ***piwil4*** | Piwi-like protein 4 | XM_012958238.1 |
| ***pkmyt1*** | Membrane-associated tyrosine- and threonine-specific cdc2-inhibitory kinase | XM_012970107.2 |
| ***pld6*** | Mitochondrial cardiolipin hydrolase | NM_001016875.3 |
| ***plk1*** | Serine/threonine-protein kinase PLK1 | NM_213679.2 |
| ***pms1*** | PMS1 protein homolog 1 | NM_001142901.2 |
| ***pms2*** | Mismatch repair endonuclease PMS2 | XM_002931979.4 |
| ***ppp1ca*** | Serine/threonine-protein phosphatase PP1-alpha catalytic subunit | NM_001127010.1 |
| ***ppp2r1a*** | Serine/threonine-protein phosphatase 2A 65 kDa regulatory subunit A alpha isoform | NM_204074.1 |
| ***ppp2r5a*** | Serine/threonine-protein phosphatase 2A 56 kDa regulatory subunit alpha isoform | NM_001078689.1 |
| ***ppp3ca*** | Serine/threonine-protein phosphatase 2B catalytic subunit alpha isoform | XM_012955571.2 |
| ***prc1*** | Protein regulator of cytokinesis 1 | XM_012958665.2 |
| ***prdm9*** | Histone-lysine N-methyltransferase PRDM9 | NM_001113936.1 |
| ***prkaca*** | cAMP-dependent protein kinase catalytic subunit alpha | XM_018092865.1 |
| ***psmc3ip*** | Homologous-pairing protein 2 homolog | XM_012966161.1 |
| ***pttg1*** | Securin | XM_004913488.3 |
| ***rad21*** | Double-strand-break repair protein rad21 homolog | XM_012965196.2 |
| ***rad50*** | DNA repair protein RAD50 | XM_012959771.2 |
| ***rad51*** | DNA repair protein RAD51 homolog 1 | NM_001016393.3 |
| ***rad51ap1*** | RAD51-associated protein 1 | XM_002940303.4 |
| ***rad52*** | DNA repair protein RAD52 homolog | NM_001097220.1 |
| ***rad54l*** | DNA repair and recombination protein RAD54-like | NM_001001241.2 |
| ***rbbp8*** | DNA endonuclease RBBP8 | XM_012965325.2 |
| ***rec114*** | Meiotic recombination protein REC114 | NM_028598.1* |
| ***rec8*** | Meiotic recombination protein REC8 homolog | NM_001079137.1 |
| ***recq1*** | ATP-dependent DNA helicase Q1 | NM_001016316.2 |
| ***rpa1*** | Replication protein A 70 kDa DNA-binding subunit | NM_001015732.1 |
| ***rpa2*** | Replication protein A 32 kDa subunit | NM_001006794.1 |
| ***rpa3*** | Replication protein A 14 kDa subunit | NM_001016626.2 |
| ***rps6ka3*** | Ribosomal protein S6 kinase alpha-3 | XM_002936044.4 |
| ***sfr1*** | Swi5-dependent recombination DNA repair protein 1 homolog | XM_012967754.2 |
| ***sgo1*** | Shugoshin 1 | XM_012964257.2 |
| ***skp1*** | S-phase kinase-associated protein 1 | XM_012959026.2 |
| ***smc1a*** | Structural maintenance of chromosomes protein 1A | XM_002935514.3 |
| ***smc1b*** | Structural maintenance of chromosomes protein 1B | NM_001278349.2 |
| ***smc3*** | Structural maintenance of chromosomes protein 3 | XM_002939498.4 |
| ***spata1*** | Spermatogenesis-associated protein 1 | XM_002931696.4 |
| ***spata2*** | Spermatogenesis-associated protein 2 | XM_002933154.4 |
| ***spata22*** | Spermatogenesis-associated protein 22 | XM_012957517.2 |
| ***spata4*** | Spermatogenesis-associated protein 4 | XM_002933324.4 |
| ***spata5*** | Spermatogenesis-associated protein 5 | XM_004911116.3 |
| ***spdyc*** | Speedy protein C | XM_004913654.3 |
| ***spo11*** | Meiotic recombination protein SPO11 | NM_001008199.1 |
| ***stra8*** | Stimulated by retinoic acid gene 8 protein homolog | XM_018089880.1 |
| ***sumo1*** | Small ubiquitin-related modifier 1 | NM_001005111.1 |
| ***sun1*** | SUN domain-containing protein 1 | XM_012971093.2 |
| ***suv39h1*** | Histone-lysine N-methyltransferase SUV39H1 | XM_002944399 |
| ***syce2*** | Synaptonemal complex central element protein 2 | XM_018092692.1 |
| ***syce3*** | Synaptonemal complex central element protein 3 | XM_002939528.2 |
| ***sycp1*** | Synaptonemal complex protein 1 | XM_018091546.1 |
| ***sycp3*** | Synaptonemal complex protein 3 | NM_001078758.1 |
| ***tdrd1*** | Tudor domain-containing protein 1 | XM_004919374.3 |
| ***tdrd3*** | Tudor domain-containing protein 3 | NM_204054.1 |
| ***tdrd5*** | Tudor domain-containing protein 5 | NM_001079295.1 |
| ***tdrd9*** | Tudor domain-containing protein 9 | NM_001251817.1 |
| ***tex11*** | Testis-expressed protein 11 | XM_018096967.1 |
| ***tex12*** | Testis-expressed protein 12 | AF285582.1* |
| ***tex14*** | Testis-expressed protein 14 like | NM_001128646.1 |
| ***topbp1*** | DNA topoisomerase 2-binding protein 1 | XM_002937745.3 |
| ***trip13*** | Pachytene checkpoint protein 2 homolog | XM_012964448.2 |
| ***ttk*** | Dual specificity protein kinase TTK | XM_012963159.2 |
| ***wee1*** | Wee1-like protein kinase | NM_001126965.1 |
| ***ywhaz*** | 14-3-3 protein zeta/delta | NM_203842.1 |
| ***zfp36*** | mRNA decay activator protein ZFP36 | NM_001113071.1 |

**Table S2.** Basic characteristics of 160 gametogenic genes extracted from germline transcriptomes (testes) of one *P. lessonae* (LL) and three *P. ridibundus* (RR) individuals. The coding sequence length (CDSL) is given in nucleotides (nt), and the corresponding protein sequence length in amino acids (aa). For *P. ridibundus*, GC content was calculated as the arithmetic mean of individual sequence-specific values. The minimum (MIN) and maximum (MAX) of uncorrected p-distances are provided as a measure of interspecific genetic divergence. The number of exons (EXN) was determined from *P. lessonae* and/or *P. ridibundus* transcriptomic and genomic sequences. Comparisons with Xenopus tropicalis (*Xen*) were made to identify highly conserved genes. Uncertain results are marked with “?”; n.a.: not applicable.

| **Gene** | **Parameter** | | | | | | |  | **Divergence LL–RR** [%] | | | |
| --- | --- | --- | --- | --- | --- | --- | --- | --- | --- | --- | --- | --- |
|  | **CDSL (nt)** | | **CDSL (aa)** | | **EXN** | **GC [%[** | | | **p_nt_** | | **p_aa_** | |
|  | **LL** | **RR** | **LL** | **RR** |  | **LL** | **RR** | ***Xen*** | **MIN** | **MAX** | **MIN** | **MAX** |
| ***adarb1*** | 2154 | 2154 | 717 | 717 | 9 | 44.90 | 44.80 | 43.87 | 1.07 | 1.11 | 0.28 | 0.28 |
| ***adcy9*** | 3924 | 3924 | 1307 | 1307 | 10 | 48.70 | 48.80 | 50.64 | 1.15 | 1.33 | 1.00 | 1.07 |
| ***ago2*** | 2595 | 2622/2595 | 864 | 873/864 | 19 | 46.40 | 46.30 | 47.16 | 0.94 | 0.94 | 0.12 | 0.12 |
| ***ago3*** | 2583 | n.a. | 860 | n.a. | 19 | 47.90 | n.a. | 48.16 | n.a. | n.a. | n.a. | n.a. |
| ***ago4*** | 2589 | 2589 | 862 | 862 | 17 | 46.90 | 47.10 | 46.93 | 0.46 | 0.50 | 0.12 | 0.12 |
| ***akt1*** | 1443 | 1443 | 480 | 480 | 12 | 40.50 | 40.60 | 41.77 | 0.42 | 0.49 | 0.21 | 0.21 |
| ***anapc13*** | 225 | 225 | 74 | 74 | 2 | 46.20 | 46.00 | 47.11 | 0.44 | 1.33 | 0.00 | 0.00 |
| ***ar*** | 2334 | n.a. | 777 | n.a. | 8 | 52.90 | n.a | 56.91 | n.a. | n.a. | n.a. | n.a. |
| ***asz1*** | 1425 | 1425 | 474 | 474 | 13 | 40.70 | 41.00 | 40.13 | 0.91 | 0.91 | 0.63 | 0.63 |
| ***atr*** | 7947 | 7947 | 2648 | 2648 | 48 | 43.40 | 43.40 | 42.94 | 0.71 | 0.71 | 0.45 | 0.45 |
| ***aurka*** | 1245 | 1251 | 414 | 416 | 8 | 49.60 | 49.60 | 53.21 | 1.29 | 1.61 | 1.21 | 1.93 |
| ***bcar1*** | 2856 | 2871/2856 | 951 | 956/951 | 6 | 47.20 | 46.70 | 44.47 | 0.87 | 0.90 | 0.43 | 0.54 |
| ***bcar3*** | 2451 | 2448 | 816 | 815 | 11 | 45.50 | 45.40 | 45.08 | 1.06 | 1.27 | 0.86 | 0.98 |
| ***birc5*** | 525 | 525 | 174 | 174 | 4 | 55.30 | 54.90 | 49.07 | 1.71 | 1.71 | 1.15 | 1.15 |
| ***brd2*** | 2343 | 2343 | 780 | 780 | 13 | 48.00 | 48.10 | 39.45 | 0.38 | 0.51 | 0.00 | 0.00 |
| ***btrc*** | 1674 | 1674 | 557 | 557 | 12 | 45.80 | 47.80 | 46.16 | 0.48 | 0.54 | 0.00 | 0.18 |
| ***bub1*** | 3321 | 3345/3348 | 1106 | 1114/1115 | 25 | 44.60 | 44.80 | 44.32 | 1.51 | 1.57 | 1.27 | 1.36 |
| ***bub3*** | 975 | 975 | 324 | 324 | 6 | 48.10 | 48.10 | 48.62 | 0.31 | 0.51 | 0.00 | 0.00 |
| ***calm*** | 450 | 450 | 149 | 149 | 5 | 43.30 | 43.30 | 40.67 | 0.00 | 0.00 | 0.00 | 0.00 |
| ***camk2g*** | 1554 | 1554 | 517 | 517 | 19 | 45.50 | 45.70 | 46.09 | 0.77 | 0.90 | 0.19 | 0.19 |
| ***ccna1*** | 1254 | 1254 | 417 | 417 | 8 | 45.90 | 46.10 | 47.54 | 1.52 | 1.68 | 2.16 | 2.40 |
| ***ccnb1*** | 1194 | 1194 | 397 | 397 | 8? | 54.50 | 54.50 | 44.72 | 1.59 | 1.59 | 1.01 | 1.01 |
| ***ccnb1ip1*** | 804 | 804 | 267 | 267 | 3 | 44.40 | 44.50 | 44.36 | 1.12 | 1.37 | 0.00 | 0.00 |
| ***ccnb2*** | 1176 | 1176 | 391 | 391 | 9 | 45.70 | 46.20 | 45.61 | 1.70 | 1.70 | 1.02 | 1.02 |
| ***ccnb3*** | 1251 | 1248 | 416 | 415 | 10 | 47.10 | 46.40 | 45.08 | 2.08 | 2.08 | 1.45 | 1.45 |
| ***ccne2*** | 1212 | 1212 | 403 | 403 | 10? | 42.60 | 42.80 | 43.55 | 0.99 | 0.99 | 1.24 | 1.24 |
| ***cdc20*** | 1527 | 1527 | 508 | 508 | 10 | 52.80 | 52.70 | 44.23 | 1.83 | 1.83 | 0.79 | 0.79 |
| ***cdc25c*** | 1635 | 1635 | 544 | 544 | 16 | 44.50 | 44.40 | 44.66 | 1.10 | 1.10 | 1.29 | 1.29 |
| ***cdk1*** | 909 | 909 | 302 | 302 | 7 | 41.70 | 41.50 | 45.61 | 0.33 | 0.44 | 0.33 | 0.33 |
| ***cdk2*** | 894 | 894 | 297 | 297 | 7 | 48.50 | 48.20 | 46.53 | 1.01 | 1.01 | 0.34 | 0.34 |
| ***cdk7*** | 1059 | 1059 | 352 | 352 | 11 | 43.80 | 43.70 | 44.00 | 0.00 | 1.13 | 0.00 | 0.28 |
| ***cpeb4*** | 2130 | 2130 | 709 | 709 | 8 | 48.70 | 49.00 | 47.14 | 0.47 | 0.56 | 0.14 | 0.14 |
| ***ctcf*** | 2205 | 2205 | 734 | 734 | 10 | 42.60 | 42.60 | 44.31 | 0.45 | 0.54 | 0.27 | 0.27 |
| ***cyp26b1*** | 1536 | 1536 | 511 | 511 | 6 | 53.70 | 53.30 | 52.80 | 1.17 | 1.17 | 0.20 | 0.20 |
| ***dazl*** | 849 | 849 | 282 | 282 | 11? | 43.90 | 43.70 | 44.60 | 0.12 | 0.12 | 0.36 | 0.36 |
| ***ddx19*** | 1479 | 1479 | 492 | 492 | n.a. | 53.10 | 52.80 | 53.89 | 1.42 | 1.56 | 0.81 | 0.81 |
| ***ddx25*** | 1464 | 1467 | 487 | 488 | 8 | 46.80 | 45.60 | 40.69 | 2.87 | 3.21 | 2.05 | 2.46 |
| ***ddx4*** | 2226 | 2226 | 741 | 741 | n.a. | 42.40 | 42.50 | 42.08 | 1.21 | 1.21 | 0.95 | 1.08 |
| ***dmc1*** | 1026 | 1026 | 341 | 341 | 13 | 41.00 | 41.00 | 41.23 | 0.68 | 0.78 | 0.59 | 0.59 |
| ***dmrt1*** | 1005 | 1005 | 334 | 334 | 5 | 48.60 | 47.10 | 48.17 | 1.99 | 2.09 | 2.99 | 2.99 |
| ***dmrt2*** | 1566 | n.a. | 521 | n.a. | 3 | 49.60 | n.a. | 49.46 | n.a. | n.a. | n.a. | n.a. |
| ***dmrt5*** | 1317 | n.a. | 438 | n.a. | 2 | 58.10 | n.a. | 55.18 | n.a. | n.a. | n.a. | n.a. |
| ***dnmt1*** | 4470/4476 | 4476 | 1489/1491 | 1491 | 32 | 45.90 | 46.10 | 46.55 | 0.87 | 0.87 | 0.67 | 0.67 |
| ***dnmt3a*** | 2682 | 2682 | 893 | 893 | 21 | 52.80 | 52.90 | 52.91 | 1.16 | 1.16 | 0.45 | 0.45 |
| ***dusp1*** | 1110 | 1110 | 369 | 369 | 4 | 49.70 | 49.60 | 48.56 | 0.81 | 0.99 | 0.00 | 0.00 |
| ***eif4g3*** | 5160 | 5160 | 1719 | 1719 | n.a. | 57.00 | 56.90 | 57.48 | 1.32 | 1.45 | 0.99 | 1.05 |
| ***eme1*** | 777 | 777 | 258 | 258 | 6 | 49.80 | 49.30 | 55.53 | 1.80 | 1.80 | 3.49 | 3.49 |
| ***eme2*** | 1560 | 1560 | 519 | 519 | 8 | 47.90 | 47.90 | 49.54 | 1.73 | 2.12 | 2.51 | 2.89 |
| ***exd1*** | 1155 | 1155 | 384 | 384 | 10 | 42.30 | 42.30 | 44.16 | 0.00 | 0.00 | 0.00 | 0.00 |
| ***fbxo43*** | 2157 | 2160 | 718 | 719 | 5 | 42.20 | 41.70 | 43.06 | 1.72 | 1.76 | 2.24 | 2.37 |
| ***fbxo5*** | 1212 | 1170 | 403 | 389 | 4 | 41.60 | 41.50 | 40.56 | 1.45 | 1.97 | 1.55 | 2.32 |
| ***fgf9*** | 630 | 630 | 209 | 209 | 3 | 46.60 | 46.60 | 48.80 | 0.16 | 0.48 | 0.00 | 0.00 |
| ***fkbp6*** | 990 | 990 | 329 | 329 | 8 | 45.00 | 45.40 | 45.58 | 1.16 | 1.57 | 1.06 | 1.37 |
| ***foxc1*** | 1482 | 1488 | 493 | 495 | 1? | 54.90 | 54.60 | 59.61 | 0.88 | 1.01 | 0.41 | 0.61 |
| ***grb2*** | 690 | 690 | 229 | 229 | 5 | 46.60 | 47.20 | 49.86 | 1.45 | 1.59 | 0.00 | 0.00 |
| ***h3.3*** | 411 | 411 | 136 | 136 | 3 | 56.00 | 56.20 | 55.23 | 0.24 | 0.24 | 0.00 | 0.00 |
| ***hat1*** | 1227 | 1227 | 408 | 408 | 11 | 42.80 | 42.40 | 42.35 | 0.73 | 0.73 | 0.74 | 0.74 |
| ***hdac1*** | 1443 | 1443 | 480 | 480 | 14 | 42.80 | 42.90 | 42.97 | 0.62 | 0.69 | 0.00 | 0.00 |
| ***hec1*** | 1917 | 1917 | 638 | 638 | 16 | 51.80 | 51.70 | 53.15 | 1.15 | 1.57 | 0.63 | 1.41 |
| ***henmt1*** | 1125 | 1125 | 374 | 374 | 6 | 39.70 | 39.50 | 42.61 | 0.80 | 1.16 | 1.60 | 1.87 |
| ***hormad1*** | 1161 | 1161 | 386 | 386 | 12 | 44.40 | 44.60 | 43.90 | 0.86 | 1.03 | 1.04 | 1.04 |
| ***igf1*** | 462 | 462 | 153 | 153 | 4 | 45.40 | 44.70 | 45.45 | 1.08 | 1.08 | 0.65 | 0.65 |
| ***igf1r*** | 3330 | 3327 | 1109 | 1108 | 18 | 43.50 | 44.60 | 47.84 | 0.64 | 0.64 | 0.83 | 0.83 |
| ***ints13*** | 2157 | 2157 | 718 | 718 | 17 | 48.60 | 48.50 | 56.17 | 0.37 | 1.39 | 0.14 | 0.98 |
| ***itpr1*** | 8049 | 8043 | 2682 | 2680 | 47? | 52.50 | 52.70 | 46.49 | 1.50 | 1.53 | 0.37 | 0.37 |
| ***kif22*** | 1929 | 1929 | 642 | 642 | 13 | 49.70 | 49.60 | 57.85 | 0.52 | 0.83 | 0.31 | 0.47 |
| ***kmt2a*** | 7983 | n.a. | 2660 | n.a. | 31 | 45.50 | 45.60 | 46.00 | 0.89 | 0.89 | 0.96 | 0.96 |
| ***m1ap*** | 1461 | 1464 | 486 | 487 | 9 | 56.20 | 56.30 | 54.93 | 2.26 | 2.26 | 2.06 | 2.06 |
| ***mad2l1*** | 612 | 612 | 203 | 203 | 5 | 44.50 | 44.30 | 40.85 | 0.65 | 0.65 | 0.49 | 0.49 |
| ***mael*** | 1266 | 1263 | 421 | 420 | 13 | 46.00 | 46.40 | 45.91 | 2.69 | 2.69 | 3.10 | 3.10 |
| ***map2k1*** | 1227 | 1227 | 408 | 408 | 11 | 51.90 | 51.70 | 48.40 | 0.73 | 0.90 | 0.00 | 0.00 |
| ***map3k7*** | 1827 | 1827 | 608 | 608 | 17 | 44.00 | 44.10 | 42.82 | 0.38 | 0.44 | 0.33 | 0.33 |
| ***mapk1*** | 1089 | 1089 | 362 | 362 | 8 | 43.80 | 43.90 | 43.74 | 0.37 | 0.46 | 0.00 | 0.00 |
| ***mapk12*** | 1089 | 1089 | 362 | 362 | 12 | 44.60 | 44.50 | 42.14 | 0.83 | 0.92 | 0.28 | 0.28 |
| ***marf1*** | 5064 | 5064 | 1687 | 1687 | 25 | 43.50 | 43.70 | 43.32 | 1.26 | 1.32 | 1.36 | 1.54 |
| ***mdc1*** | 6771 | 6708 | 2256 | 2235 | 16 | 45.80 | 45.70 | 41.53 | 2.91 | 2.95 | 5.33 | 5.42 |
| ***mei4*** | 1212 | n.a. | 403 | n.a. | 5 | 36.80 | 38.20 | 36.39 | 1.38 | 1.38 | 2.07 | 2.07 |
| ***meig1*** | 276 | 276 | 91 | 91 | 2 | 38.70 | 38.40 | 35.96 | 0.36 | 0.36 | 0.00 | 0.00 |
| ***meiob*** | 1419 | 1419 | 472 | 472 | 13 | 43.70 | 43.70 | 42.50 | 1.34 | 1.34 | 1.06 | 1.06 |
| ***mlh1*** | 2265 | 2265 | 754 | 754 | 19 | 42.40 | 42.20 | 41.56 | 1.19 | 1.46 | 0.93 | 1.19 |
| ***mlh3*** | 3681 | 3708 | 1226 | 1235 | 12 | 49.00 | 48.60 | 39.34 | 2.43 | 2.43 | 4.15 | 4.32 |
| ***mnd1*** | 618 | 618 | 205 | 205 | 8 | 43.40 | 42.90 | 41.19 | 0.16 | 1.78 | 0.00 | 2.44 |
| ***mns1*** | 1500 | 1500 | 499 | 499 | 9 | 43.60 | 43.30 | 42.02 | 1.07 | 1.13 | 0.80 | 1.00 |
| ***mov10l1*** | 3690 | 3687 | 1229 | 1228 | 26 | 41.30 | 41.20 | 42.09 | 1.00 | 1.00 | 1.06 | 1.06 |
| ***mre11*** | 2136 | 2136 | 711 | 711 | 19 | 45.20 | 44.80 | 46.95 | 1.26 | 1.40 | 0.42 | 0.84 |
| ***msh2*** | 2793 | 2793 | 930 | 930 | 16 | 47.20 | 47.10 | 44.98 | 1.33 | 1.33 | 0.43 | 0.43 |
| ***msh4*** | 2289 | 2289 | 762 | 762 | 18 | 41.50 | 41.40 | 37.46 | 1.32 | 1.59 | 0.66 | 0.80 |
| ***msh5*** | 2424 | 2424 | 807 | 807 | 25 | 43.80 | 43.70 | 42.68 | 0.87 | 0.95 | 0.74 | 0.74 |
| ***msh6*** | 4017 | 4017 | 1338 | 1338 | 10 | 46.20 | 46.20 | 45.36 | 1.22 | 1.22 | 0.90 | 0.97 |
| ***msx1*** | 843 | 843 | 280 | 280 | 2 | 50.80 | 50.70 | 57.00 | 0.71 | 0.83 | 1.07 | 1.07 |
| ***mus81*** | 1818 | 1818 | 605 | 605 | 16 | 44.00 | 43.90 | 46.33 | 0.99 | 0.99 | 1.33 | 1.82 |
| ***mybl1*** | 804 | 804 | 267 | 267 | 3 | 44.60 | 44.50 | 43.05 | 0.00 | 0.25 | 0.00 | 0.00 |
| ***nbn*** | 2301 | 2292/2289 | 766 | 763/762 | 16 | 41.50 | 41.80 | 42.53 | 1.53 | 1.66 | 2.23 | 2.49 |
| ***nusap1*** | 1263 | 1263 | 420 | 420 | 11 | 41.60 | 42.10 | 46.20 | 0.00 | 0.08 | 0.00 | 0.24 |
| ***parn*** | 1905 | 1905 | 634 | 634 | 24 | 40.10 | 40.20 | 40.18 | 1.68 | 1.79 | 1.42 | 1.74 |
| ***pelp1*** | 3270 | 3270 | 1089 | 1089 | 16 | 52.20 | 52.40 | 57.80 | 1.13 | 1.26 | 0.83 | 1.01 |
| ***piwil2*** | 2853 | 2853 | 950 | 950 | 24 | 46.10 | 46.10 | 46.42 | 1.54 | 1.68 | 1.68 | 1.90 |
| ***piwil4*** | 2502 | 2502 | 833 | 833 | 20 | 45.90 | 45.80 | 50.78 | 0.64 | 0.76 | 0.60 | 0.72 |
| ***pkmyt1*** | 1644 | 1644 | 547 | 547 | 7 | 56.20 | 56.10 | 50.91 | 1.22 | 2.07 | 0.91 | 1.83 |
| ***pld6*** | 492 | 492 | 163 | 163 | 2 | 55.60 | 55.40 | 56.71 | 1.02 | 1.42 | 0.00 | 0.61 |
| ***plk1*** | 1779 | 1779 | 592 | 592 | 10 | 51.30 | 50.90 | 50.08 | 1.57 | 1.57 | 0.68 | 0.68 |
| ***pms1*** | 2664 | 2664 | 887 | 887 | 12 | 38.60 | 38.90 | 37.31 | 1.80 | 1.84 | 3.04 | 3.27 |
| ***pms2*** | 2517 | 2517/2520 | 838 | 838/839 | 13 | 40.00 | 39.80 | 44.64 | 0.95 | 1.07 | 1.43 | 1.67 |
| ***ppp1ca*** | 984 | 984 | 327 | 327 | 7 | 48.90 | 48.80 | 57.19 | 0.00 | 1.52 | 0.00 | 0.00 |
| ***ppp2r1a*** | 1770 | 1770 | 589 | 589 | 15 | 52.20 | 51.90 | 49.44 | 1.64 | 1.75 | 0.17 | 0.17 |
| ***ppp2r5a*** | 1422 | 1422 | 473 | 473 | 13 | 43.80 | 43.90 | 43.33 | 0.84 | 0.91 | 0.21 | 0.21 |
| ***ppp3ca*** | 1533 | 1533 | 510 | 510 | 13 | 43.30 | 43.40 | 41.08 | 0.72 | 0.78 | 0.20 | 0.20 |
| ***prc1*** | 1827 | 1827 | 608 | 608 | 14 | 42.50 | 42.90 | 41.98 | 0.88 | 0.88 | 0.16 | 0.16 |
| ***prdm9*** | 1854 | 1854 | 617 | 617 | 1? | 53.20 | 53.00 | 43.95 | 1.29 | 1.67 | 1.30 | 2.11 |
| ***prkaca*** | 1056 | 1056 | 351 | 351 | 10 | 44.10 | 43.80 | 46.78 | 0.47 | 0.57 | 0.00 | 0.00 |
| ***psmc3ip*** | 663 | 645 | 220 | 214 | 2 | 43.50 | 42.90 | 41.67 | 2.33 | 2.33 | 1.87 | 2.34 |
| ***pttg1*** | 564 | 564 | 187 | 187 | 5 | 44.30 | 44.30 | 39.49 | 1.42 | 1.60 | 2.67 | 3.21 |
| ***rad21*** | 1890 | 1887 | 629 | 628 | 13 | 47.90 | 48.00 | 48.15 | 0.80 | 0.90 | 0.32 | 0.64 |
| ***rad50*** | 3939 | 3939 | 1312 | 1312 | 25 | 43.70 | 44.40 | 41.53 | 0.86 | 0.89 | 0.61 | 0.61 |
| ***rad51*** | 1008 | 1008 | 335 | 335 | 9 | 45.10 | 45.10 | 49.26 | 0.00 | 0.00 | 0.00 | 0.00 |
| ***rad51ap1*** | 1098 | 1101 | 365 | 366 | 9 | 44.30 | 44.80 | 44.93 | 1.91 | 2.00 | 1.64 | 1.92 |
| ***rad52*** | 1302 | 1290 | 433 | 429 | 11 | 50.00 | 50.00 | 54.80 | 0.16 | 1.01 | 0.47 | 1.17 |
| ***rad54l*** | 2229 | 2229 | 742 | 742 | 18 | 55.20 | 55.50 | 44.24 | 2.02 | 2.15 | 0.81 | 0.81 |
| ***rbbp8*** | 2607 | 2610 | 868 | 869 | 17 | 43.70 | 43.70 | 43.91 | 1.14 | 1.14 | 1.38 | 1.38 |
| ***rec114*** | 651 | n.a. | 216 | n.a. | 6 | 44.10 | 44.00 | n.a. | 0.77 | 0.77 | 0.93 | 0.93 |
| ***rec8*** | 2277 | 2277 | 758 | 758 | 19 | 46.30 | 46.80 | 48.03 | 1.45 | 1.45 | 1.98 | 1.98 |
| ***recq1*** | 1974 | 1974 | 657 | 657 | 15 | 47.40 | 47.40 | 57.84 | 0.91 | 1.11 | 0.46 | 0.76 |
| ***rpa1*** | 1839 | 1839 | 612 | 612 | 17 | 44.70 | 44.90 | 43.44 | 1.09 | 1.14 | 0.65 | 0.65 |
| ***rpa2*** | 831 | 831 | 276 | 276 | 9 | 44.30 | 44.80 | 45.41 | 0.60 | 0.84 | 0.00 | 0.00 |
| ***rpa3*** | 366 | 366 | 121 | 121 | 4 | 48.40 | 47.90 | 44.26 | 0.27 | 3.01 | 0.00 | 2.48 |
| ***rps6ka3*** | 2214 | 2214 | 737 | 737 | 22 | 43.70 | 43.90 | 43.34 | 1.08 | 1.13 | 0.27 | 0.27 |
| ***sfr1*** | 729 | 714 | 242 | 237 | 3 | 44.60 | 45.20 | 40.29 | 1.96 | 1.96 | 2.95 | 2.95 |
| ***sgo1*** | 2016 | 2019 | 671 | 672 | 9 | 41.60 | 41.40 | 40.87 | 1.75 | 1.75 | 2.64 | 2.64 |
| ***skp1*** | 492 | 492 | 163 | 163 | 5 | 39.90 | 40.10 | 40.24 | 0.41 | 0.41 | 0.00 | 0.00 |
| ***smc1a*** | 3687 | 3687 | 1228 | 1228 | 25 | 49.20 | 48.90 | 46.23 | 1.22 | 1.25 | 0.00 | 0.08 |
| ***smc1b*** | 3687 | 3687 | 1228 | 1228 | 25 | 40.80 | 40.60 | 46.51 | 0.49 | 0.54 | 0.24 | 0.41 |
| ***smc3*** | 3654 | 3654 | 1217 | 1217 | 8 | 44.70 | 45.10 | 46.66 | 0.85 | 0.85 | 0.08 | 0.08 |
| ***spata1*** | 1497 | n.a. | 498 | n.a. | 12 | 46.70 | n.a. | 40.58 | n.a. | n.a. | n.a. | n.a. |
| ***spata2*** | 1575 | 1575 | 524 | 524 | 2 | 49.70 | 50.00 | 45.96 | 1.53 | 1.59 | 0.95 | 0.95 |
| ***spata22*** | 957 | 957 | 318 | 318 | 7 | 39.20 | 38.50 | 38.76 | 1.46 | 1.57 | 2.83 | 3.15 |
| ***spata4*** | 816 | 816 | 271 | 271 | 6 | 43.20 | 42.80 | 44.73 | 0.98 | 1.10 | 0.00 | 0.00 |
| ***spata5*** | 2565 | 2565 | 854 | 854 | 17 | 45.40 | 45.60 | 45.28 | 1.48 | 1.48 | 1.41 | 1.41 |
| ***spdyc*** | 876 | 876 | 291 | 291 | 6 | 49.10 | 48.80 | 47.10 | 0.57 | 0.69 | 0.34 | 0.34 |
| ***spo11*** | 1206 | 1206 | 401 | 401 | 13 | 49.20 | 48.70 | 45.81 | 1.24 | 1.74 | 1.25 | 1.50 |
| ***stra8*** | 807 | 807 | 268 | 268 | 7 | 48.80 | 49.10 | 52.78 | 1.12 | 1.12 | 1.87 | 1.87 |
| ***sumo1*** | 309 | 309 | 102 | 102 | 5 | 42.40 | 42.70 | 42.07 | 0.65 | 0.65 | 0.98 | 0.98 |
| ***sun1*** | 2733 | 2727 | 910 | 908 | n.a. | 44.30 | 44.00 | 44.10 | 1.03 | 1.06 | 1.10 | 1.21 |
| ***suv39h1*** | 1275 | 1275 | 424 | 424 | 6 | 46.80 | 46.90 | 60.38 | 0.63 | 0.63 | 0.94 | 0.94 |
| ***syce2*** | 507 | 507 | 168 | 168 | 3 | 46.80 | 47.10 | 46.27 | 1.58 | 1.78 | 2.38 | 2.98 |
| ***syce3*** | 267 | 267 | 88 | 88 | 2 | 44.20 | 44.50 | 43.88 | 0.00 | 0.75 | 0.00 | 0.00 |
| ***sycp1*** | 2994 | 2994 | 997 | 997 | 30 | 34.90 | 34.80 | 34.81 | 0.77 | 1.00 | 1.20 | 2.01 |
| ***sycp3*** | 717 | 717 | 238 | 238 | 8 | 39.00 | 38.50 | 41.38 | 0.84 | 0.84 | 1.26 | 1.26 |
| ***tdrd1*** | 4014 | 4017/4005 | 1337 | 1338/1334 | 22 | 44.90 | 45.10 | 50.81 | 0.58 | 1.19 | 1.71 | 2.26 |
| ***tdrd3*** | 2232 | 2232 | 743 | 743 | 13 | 42.7 | 42.50 | 41.96 | 1.21 | 1.34 | 1.48 | 1.62 |
| ***tdrd5*** | 3249 | 3249 | 1082 | 1082 | 16 | 43.80 | 43.70 | 41.91 | 0.62 | 0.62 | 1.20 | 1.20 |
| ***tdrd9*** | 4038 | 4038 | 1345 | 1345 | 36 | 43.00 | 43.10 | 38.66 | 0.84 | 0.84 | 0.97 | 0.97 |
| ***tex11*** | 2763 | 2763 | 920 | 920 | 29 | 50.00 | 49.50 | 43.19 | 2.03 | 2.03 | 2.94 | 2.94 |
| ***tex12*** | 354 | 354 | 117 | 117 | 5 | 38.70 | 39.60 | n.a. | 1.41 | 1.41 | 2.56 | 2.56 |
| ***tex14*** | 3369 | 3381 | 1122 | 1126 | 26 | 40.10 | 40.00 | 37.66 | 1.10 | 1.10 | 1.87 | 1.87 |
| ***topbp1*** | 4548 | 4548 | 1515 | 1515 | 27 | 44.10 | 44.00 | 43.34 | 0.99 | 0.99 | 0.92 | 0.99 |
| ***trip13*** | 1299 | 1299 | 432 | 432 | 13 | 38.40 | 38.50 | 38.17 | 1.31 | 1.31 | 0.93 | 0.93 |
| ***ttk*** | 2628 | 2622 | 875 | 873 | 18 | 40.50 | 40.60 | 40.61 | 1.76 | 1.76 | 2.41 | 2.41 |
| ***wee1*** | 1731 | 1731 | 576 | 576 | 11 | 56.40 | 56.00 | 56.64 | 1.10 | 1.73 | 0.52 | 0.69 |
| ***ywhaz*** | 741 | 741 | 246 | 246 | 5 | 46.90 | 47.10 | 45.93 | 0.41 | 0.41 | 0.00 | 0.00 |
| ***zfp36*** | 1134 | 1131 | 377 | 376 | 2 | 58.40 | 57.60 | 50.11 | 1.42 | 1.59 | 0.27 | 0.80 |

**Table S3**. Best-fit models and statistical parameters of simple regressions performed with the number of exons (EXN), coding sequence length (CDSL), and GC content (GC) of gametogenic genes obtained from the transcriptomes of *P. lessonae* (LL) and *P. ridibundus* (RR). The Durbin-Watson statistic (DWS) was used to assess serial correlation in the residuals; a value close to 2 indicates that the residuals vary randomly. Significant probability values (*p*) are highlighted in bold. N: sample size.

| **Species** | **N** | **Y** | **X** | **Model** | **R² [%]** | **ANOVA** | | **DWS (*p*)** |
| --- | --- | --- | --- | --- | --- | --- | --- | --- |
|  |  |  |  |  |  | **F** | ***p*** |  |
| LL | 156 | EXN | CDSL | Y = –7.32 + 0.475 ∙ √(X) | 64.7 | 282.55 | <0.001 | 1.82 (0.134) |
|  | 160 | GC | CDSL | Y = 1/(0.0218 + 0.193 ÷ X) | 0.4 | 0.64 | 0.422 | 1.72 **(0.040)** |
|  | 156 | GC | EXN | Y = √(2.0 ∙ 10^3^ + 889 ÷ X) | 9.9 | 16.94 | <0.001 | 1.87 (0.213) |
| RR | 149 | EXN | CDSL | Y = √(61.7 + 3.0 ∙ 10^-5^ ∙ X²) | 68.2 | 314,86 | <0.001 | 2.02 (0.538) |
|  | 153 | GC | CDSL | Y = 1/(2.2∙ 10^-2^+ 0.187 ÷ X) | 0.06 | 0.62 | 0.432 | 1.77 (0.074) |
|  | 152 | GC | EXN | Y = √(2.02 ∙ 10^3^ + 750 ÷ X) | 7.4 | 11.86 | <0.001 | 1.88 (0.226) |

**Table S4**. Best-fit models (simple linear rgressions) for the relationships between the dependent parameter genetic distance (p_nt_), based on nucleotide sequences of gametogenic genes, and the independent parameters GC content, coding sequence length (CDSL), and number of exons (EXN). Regression analyses were conducted for both interspecific comparisons (*P. lessonae* [LL] vs. *P. ridibundus* [RR]) and intraspecific comparisons among RR individuals (RR1–RR3). The best-fitting model for each relationship was selected based on the highest R² value. The Durbin-Watson statistic (DWS) was used to assess serial correlation in the residuals, with values close to 2 indicating random variation. Significant probability values (*p*) are highlighted in bold. N: sample size.

| **Comparison** | **X** | **N** | **Best fit model** | **R^2^ [%]** | **ANOVA** | | **DWS (*p*)** |
| --- | --- | --- | --- | --- | --- | --- | --- |
|  |  |  |  |  | **F** | ***p*** |  |
| LL-RR | GC_mean_ | 155 | p_nt_ = 4.55 ∙ 10^-3^ + 3.3 ∙ 10^-6^ ∙ X² | 5.70 | 9.25 | **0.003** | 2.03 (0.568) |
|  | CDSL_mean_ | 152 | p_nt_ = (0.038 + 8.79 ∙ 10^-3^ ∙ ln(X))^2^ | 4.02 | 6.29 | **0.013** | 2.04 (0.597) |
|  | EXN | 151 | p_nt_ = √(1.66 ∙ 10^-4^ – 2.35 ∙ 10^-8^ ∙ X²) | 0.25 | 0.37 | 0.543 | 2.13 (0.780) |
| RR (1-3) | GC | 148 | p_nt_ = -4.06 ∙ 10^-3^ + 1.36 ∙ 10^-4^∙ X | 3.94 | 5.98 | **0.016** | 2.19 (0.880) |
|  | CDSL | 148 | p_nt_ = √(5.80 ∙ 10^-6^ + 9.78 ∙ 10^-3^ ÷ X) | 3.15 | 4.75 | **0.031** | 2.16 (0.841) |
|  | EXN | 144 | p_nt_ = 1.7 ∙ 10^-3^ + 3.45 ∙ 10^-3^ ÷ X | 2.87 | 4.19 | **0.042** | 2.22 (0.910) |

**Table S5.** Single nucleotide polymorphisms (SNPs) and corresponding amino acid substitutions (aa) detected by GT-Seq in 52 gametogenic genes of *P. lessonae* (LL) and *P. ridibundus* (RR). SNP alleles considered to be species-specific are shown in **bold**; putatively introgressed alleles are marked with asterisks (*). Nucleotide (nt) T of SNP59 was observed only in *P. esculentus* (LR). Country abbreviations: CZ, Czech Republic; HR, Croatia; DE, Germany.

| **SNP** | **Gene** | **Exon** | **Substitution** | | **SNP alleles** | | |
| --- | --- | --- | --- | --- | --- | --- | --- |
|  |  |  | **nt** | **aa** | **LL [CZ, HR]** | **LL [DE]** | **RR** |
| 1 | *adcy9* | 6 | A/G | - | **A** | **A** | **G**, A* |
| 2 | *adcy9* | 6 | C/T | - | **C** | **C** | **T**, C* |
| 3 | *brd2* | 2 | C/T | - | T | T | C, T |
| 4 | *brd2* | 4 | A/G | - | A | A | A, G |
| 5 | *brd2* | 5 | A/T | - | A | A | A, T |
| 6 | *btrc* | 6 | A/G | - | **A** | **A** | **G** |
| 7 | *bub3* | 1 | A/G | - | **G** | **G** | **A** |
| 8 | *bub3* | 3 | C/T | - | C | C | C, T |
| 9 | *camk2g* | 19 | C/T | - | C | C | C, T |
| 10 | *camk2g* | 19 | C/T | - | **T** | **T** | **C** |
| 11 | *camk2g* | 19 | A/C | - | **A** | **A** | **C** |
| 12 | *cdc20* | 1 | C/T | - | C | C | C, T |
| 13 | *cdc25c1* | 10 | A/G | - | **A** | **A** | **G** |
| 14 | *cdk2* | 4 | A/T | - | **A** | **A** | **T** |
| 15 | *cdk2* | 6 | G/T | - | **G** | **G** | **T** |
| 16 | *cpeb4* | 1 | A/G | - | A | A | A, G |
| 17 | *cpeb4* | 7 | C/T | - | C | C | C, T |
| 18 | *ctcf* | 1 | C/T | - | **T** | **T** | **C** |
| 19 | *ctcf* | 1 | A/G | - | G | G | A, G |
| 20 | *fbxo43* | 2 | A/G | **G/R** | **G** | **G** | **A** |
| 21 | *fbxo43* | 5 | A/C | - | **A** | **A** | **C** |
| 22 | *fbxo43* | 5 | C/T | - | C | C | C, T |
| 23 | *fbxo5* | 1 | G/T | - | G,T | G,T | G |
| 24 | *hat1* | 9 | C/T | - | **C** | **C** | **T**, C* |
| 25 | *henmt1* | 3 | C/T | - | **C** | **C** | **T,** C* |
| 26 | *henmt1* | 4 | C/T | - | T | T | C, T |
| 27 | *henmt1* | 4 | A/C | - | C | C | A, C |
| 28 | *henmt1* | 6 | A/G | A/T | G | G | A, G |
| 29 | *hop2* | 1 | A/C | - | **C** | **C** | **A** |
| 30 | *hormad1* | 7 | C/T | - | **C** | **C** | **T**, C* |
| 31 | *hormad1* | 7 | C/T | - | C, T | C, T | C, T |
| 32 | *hormad1* | 7 | A/G | - | **A** | **A** | **G**, A* |
| 33 | *igf1r* | 2 | A/G | - | **A** | **A** | **G** |
| 34 | *igf1r* | 6 | A/G | - | A | A | A, G |
| 35 | *igf1r* | 6 | C/T | - | **T** | **T** | **C** |
| 36 | *igf1r* | 18 | G/T | - | **G** | **G** | **T** |
| 37 | *itpr1* | 5 | C/G | - | C | C | C, G |
| 38 | *itpr1* | 5 | A/C | - | C | C | A, C |
| 39 | *itpr1* | 12 | A/G | - | **G** | **G** | **A** |
| 40 | *itpr1* | 12 | C/T | - | C, T | C, T | T |
| 41 | *itpr1* | 13 | C/T | - | **T** | **T** | **C** |
| 42 | *itpr1* | 21 | A/G | - | G | G | A, G |
| 43 | *itpr1* | 21 | C/T | - | T | T | C, T |
| 44 | *itpr1* | 21 | A/G | - | **A** | **A** | **G** |
| 45 | *itpr1* | 29 | C/T | - | **T**, C* | - | **C** |
| 46 | *itpr1* | 34 | A/C | - | **A** | **A** | **C** |
| 47 | *kif22* | 2 | A/G | - | A | A, G | A, G |
| 48 | *kif22* | 5 | A/C/G | - | A, C | C, G | C, G |
| 49 | *kif22* | 6 | A/G | - | A | A, G | A, G |
| 50 | *kif22* | 6 | C/T | - | C | C, T | C, T |
| 51 | *kif22* | 6 | A/G | - | A | A, G | A, G |
| 52 | *kmt2a* | 7 | A/G | - | A, G | G | A |
| 53 | *kmt2a* | 10 | C/T | - | **T** | **T** | **C** |
| 54 | *kmt2a* | 10 | A/G | - | **G** | **G** | **A** |
| 55 | *kmt2a* | 31 | A/G | - | **G** | **G** | **A** |
| 56 | *m1ap* | 1 | C/T | - | **T** | **T** | **C** |
| 57 | *m1ap* | 1 | A/G | - | A | A | A, G |
| 58 | *map2k1* | 2 | A/G | - | **A** | **A** | **G** |
| 59 | *map2k1* | 3 | C/T [LR] | - | C | C | C |
| 60 | *map2k1* | 3 | A/C | - | C | C | A, C |
| 61 | *map3k7* | 17 | G/T | - | **G** | **G** | **T**, G* |
| 62 | *mapk12* | 11 | C/T | - | **T** | **-** | **C** |
| 63 | *marf1* | 2 | G/T | - | **G** | **G** | **T** |
| 64 | *marf1* | 3 | A/C | - | A | A | A, C |
| 65 | *marf1* | 4 | C/T | - | **C** | **C** | **T** |
| 66 | *marf1* | 4 | C/G | - | C | C | C, G |
| 67 | *mlh1* | 12 | C/G | **A/G** | **G** | **G** | **C** |
| 68 | *mlh1* | 16 | G/T | - | **G** | **G** | **T** |
| 69 | *mlh1* | 3 | G/T | F/V | G | G | G, T |
| 70 | *mns1* | 1 | A/G | **I/V** | **G** | **G** | **A** |
| 71 | *mns1* | 1 | A/G | - | **G** | **G** | **A** |
| 72 | *mns1* | 7 | A/G | - | **G** | **G** | **A** |
| 73 | *mns1* | 7 | A/C | - | A | A | A, C |
| 74 | *mns1* | 7 | C/T | - | C, T | T | C, T |
| 75 | *mr11a* | 14 | A/C | - | C | C | A, C |
| 76 | *mre11* | 14 | C/G | A/G | C | C | C, G |
| 77 | *mr11a* | 14 | C/T | - | C | C | C, T |
| 78 | *msh2* | 3 | A/G | - | **G** | **G** | **A**, G* |
| 79 | *msh2* | 14 | G/T | - | **G** | **G** | **T** |
| 80 | *nbn* | 10 | A/G | N/S | G | G | A, G |
| 81 | *nusap1* | 8 | C/T | - | C | T | C, T |
| 82 | *nusap1* | 8 | A/G | - | G | G | A, G |
| 83 | *parn* | 7 | C/T | - | C, T | - | C |
| 84 | *parn* | 7 | A/G | - | **G**, A* | **-** | **A** |
| 85 | *pelp1* | 4 | C/T | - | **T** | **T** | **C** |
| 86 | *pelp1* | 4 | A/G | - | G | G | A, G |
| 87 | *piwil2* | 12 | A/C | - | **C** | **C** | **A** |
| 88 | *piwil2* | 12 | A/G | - | **A** | **A** | **G** |
| 89 | *piwil2* | 12 | C/T | - | C | C | C, T |
| 90 | *piwil2* | 12 | C/G | - | **C** | **C** | **G** |
| 91 | *piwil2* | 15 | C/G | **R/T** | **G** | **G** | **C** |
| 92 | *piwil2* | 15 | A/G | - | **G** | **G** | **A** |
| 93 | *piwil2* | 15 | C/T | **N/S** | **T** | **T** | **C** |
| 94 | *pkmyt1* | 5 | C/T | C/R | C | C, T | C, T |
| 95 | *pkmyt1* | 5 | A/C | P/T | C | C | A, C |
| 96 | *plk1* | 1 | A/G | - | G | G | A, G |
| 97 | *plk1* | 1 | A/G | - | G | G | A, G |
| 98 | *plk1* | 5 | G/T | - | **G** | **G** | **T** |
| 99 | *plk1* | 5 | C/T | - | **C** | **C**, T* | **T**, C* |
| 100 | *ppp1ca* | 3 | C/T | - | C, T | C, T | C, T |
| 101 | *ppp1ca* | 3 | A/G | - | A | A | A, G |
| 102 | *ppp1ca* | 3 | A/T | - | A, T | A, T | A, T |
| 103 | *ppp1ca* | 3 | A/G | - | A, G | A, G | A, G |
| 104 | *ppp1ca* | 3 | C/T | - | C, T | C, T | C, T |
| 105 | *ppp2r1a* | 4 | C/T | - | C, T | C | C |
| 106 | *ppp2r1a* | 4 | C/T | - | C, T | C | C, T |
| 107 | *ppp2r1a* | 4 | C/T | - | C, T | C | C, T |
| 108 | *ppp3ca* | 2 | G/T | - | **T** | **-** | **G** |
| 109 | *ppp3ca* | 2 | A/T | - | A | **-** | A, T |
| 110 | *prkaca* | 9 | A/T | - | **A** | **-** | **T** |
| 111 | *rad50* | 5 | G/T | A/S | G | G | G, T |
| 112 | *rad50* | 5 | A/G | - | **G** | **G** | **A** |
| 113 | *rad51ap1* | 5 | A/G | - | A | A | A, G |
| 114 | *rbbp8* | 6 | A/G | - | **A** | **A** | **G**, A* |
| 115 | *rps6k3* | 17 | A/C | - | **C** | **C** | **A** |
| 116 | *rps6k3* | 17 | A/G | - | G | G | A, G |
| 117 | *rps6k3* | 17 | A/C | - | **A** | **A** | **C** |
| 118 | *sfr1* | 2 | A/G | Q/R | A, G | A, G | G |
| 119 | *sfr1* | 2 | A/G | N/S | **G** | **G** | **A** |
| 120 | *sfr1* | 2 | C/T | - | T | T | C, T |
| 121 | *sfr1* | 2 | A/G | **C/Y** | **A** | **A** | **G** |
| 122 | *sgol1* | 2 | A/G | - | A, G | A | A, G |
| 123 | *smc1a* | 4 | A/G | - | G | G | A, G |
| 124 | *smc1a* | 4 | C/T | - | **C** | **C** | **T**, C* |
| 125 | *smc1a* | 4 | C/T | - | **C** | **C** | **T**, C* |
| 126 | *topbp1* | 5 | A/G | - | **G** | **G** | **A** |
| 127 | *trip13* | 2 | A/G | - | **G** | **G** | **A** |
| 128 | *trip13* | 2 | A/G | - | **A** | **A** | **G** |
| 129 | *ywhaz* | 1 | C/T | - | **T** | **T** | **C**, T* |
| 130 | *zfp36* | 2 | A/G | - | G | G | A, G |
| 131 | *zfp36* | 2 | C/T | - | **C** | **C** | **T**, C* |

**Table S6.** Sampling localities and genotypes (males, females, juveniles or subadults*) of the individuals studied. The localities of each population system are listed from north to south.

| **Locality** | **Abbr.** | **Coordinates** | | **POPSYS** | **Genotype** | | | | **Plate** |
| --- | --- | --- | --- | --- | --- | --- | --- | --- | --- |
|  |  | **North** | **East** |  | **LL** | **RR** | **ESC** | **?** |  |
|  |  |  |  |  |  |  |  |  |  |
| Cítov [CZ] | Cit | 50°21‘25.1“N | 14°24‘08.2“E | R |  | 29,24 |  |  | 1 |
| Želechovice [CZ] | Zel | 49°44‘57.4“N | 17°08‘06.2“E | R |  | 10,10 |  |  | 1 |
| Šúr (Svätý Jur) [SK] | Sur | 48°13‘44.1“N | 17°12‘15.4“E | R |  | 7,0,1* |  |  | 5 |
| Kapuvar [HU] | Kap | 47°40‘03.6“N | 17°08‘01.7“E | R |  | 11,2 |  |  | 2 |
| Oltenița, Danube [RO] | Olt | 44°04‘38.7“N | 26°37‘24.1“E | R |  | 4,1,4* |  |  | 3 |
| Veleka, Brodilovo [BU] | Bul | 42°05‘22.6“N | 27°51‘19.2“E | R |  | 4,1 |  |  | 3 |
| Elesnitza [BU] | Bul | 41°51‘41.0“N | 23°37‘49.6“E | R |  | 3,0 |  |  | 3 |
|  |  |  |  |  |  |  |  |  |  |
| Karsibor [PL] | Kar | 53°51‘07.7“N | 14°20‘05.6“E | R-E |  | 0,9,1* | 8,1,1* |  | 2 |
| Friedrichshagen [DE] |  | 52°26‘20.7“N | 13°37‘59.9“E | R-E (?) |  | 6,3 |  |  | 3 |
| Lebus [DE] | Leb | 52°25‘02.3“N | 14°32‘31.1“E | R-E |  | 49,2 | 13,0 |  | 2 |
| Dolní Benešov [CZ] | Dol | 49°55‘05.0“N | 18°05‘50.7“E | R-E |  | 1,13 | 13,0 |  | 1,2 |
| Košatka [CZ] | Kos | 49°44‘03.6“N | 18°09‘28.0“E | R-E |  | 8,16 | 17,0 |  | 1,4 |
| Albrechtičky [CZ] | Alb | 49°42‘21.7“N | 18°05‘36.8“E | R-E |  | 12,3 | 1,0 |  | 4 |
| Borovec [CZ] | Bor | 49°37‘58.6“N | 18°06‘09.0“E | R-E (?) |  | 6,0 | 7,8 | 1* | 2 |
|  |  |  |  |  |  |  |  |  |  |
| Melzower Forst [DE] | Ger | 53°10‘52.2“N | 13°56‘15.2“E | L-E | 11,9,1* |  | 13,12,39* |  | 5,6 |
| Untermühle [DE] | Ger | 53°04‘51.9“N | 12°54‘00.3“E | L-E | 1,0 |  | 1,0 |  | 5 |
| Teschendorf [DE] | Ger | 52°50‘37.2“N | 13°08‘52.5“E | L-E | 6,2 |  | 10,1 |  | 3 |
| Český Těšín [CZ] | Cet | 49°44‘39.3“N | 18°36‘29.0“E | L-E | 7,2 |  | 0,5 |  | 2,3 |
| Trnávka [CZ] | Trn | 49°40‘55.7“N | 18°11‘00.9“E | L-E | 6,5 |  | 0,4 |  | 3 |
| Bara-Grubišno [HR] | Bag | 45°42‘30.9“N | 17°09‘02.3“E | L-E | 0,2 |  | 3,3 |  | 2 |
|  |  |  |  |  |  |  |  |  |  |
| Pedersker, Bornholm [DK] | Ped | 55°02‘07.2“N | 14°59‘45.0“E | E |  |  | 34,11 |  | 6 |
| Fehmarn [DE] | Feh | 54°31‘10.1“N | 11°03‘14.1“E | E |  |  | 11,10,2* |  | 7 |
| Rügen [DE] | Rue | 54°25‘01.6“N | 13°23‘45.9“E | E | 1* |  | 45,31,4* |  | 6,7 |
| Usedom [DE] | Use | 53°57‘12.7“N | 14°05‘29.1“E | E |  |  | 12,1 |  | 6 |
| Jarzebowo, Wolin [PL] | Jar | 53°54‘32.6“N | 14°38‘45.9“E | E |  |  | 4,0,1* |  | 2 |
| Wysoka Kamienska [PL] | Wys | 53°49‘53.2“N | 14°51‘51.8“E | E |  |  | 10,11,1* |  | 2,6 |

Population system: R: all-*P. ridibundus* population, R-E: *P. ridibundus*-*P. esculentus* population,

L-E: *P. lessonae*-*P. esculentus* population, E: all-hybrid (*P. esculentus*) population, ?: classification uncertain.

Genotype: LL: *P. lessonae*, RR: *P. ridibundus*, ESC: *P. esculentus*, *juveniles and subadults of unspecified sex.

Plate: corresponging numbers of the well-plate used for GT-seq sequencing (see Supplementary Material 7)

Country abbreviations: BU, Bulgaria; CZ, Czech Republic; DE, Germany; DK, Denmark; HR, Croatia; HU, Hungary;

PL, Poland; RO, Romania; SK, Slovakia.

**Table S7.** Medians (x͂) of SNP frequencies for *Pelophylax ridibundus* (RR) and diploid *Pelophylax esculentus* (LR). Mann-Whitney-(Wilcoxon) (MWW), Kruskal-Wallis and Mood’s median tests were used to test for x͂ differences between population systems. Significant differences are highlighted in **bold**. R: all-*ridibundus* population, R-E: *ridibundus*-*esculentus* population, L-E: *lessonae*-*esculentus* population, E: all-hybrid (*esculentus*) population. n.a.: not applicable due to insufficient data.

| **SNP** | **RR** | | | |  | **LR** | | | | | | |
| --- | --- | --- | --- | --- | --- | --- | --- | --- | --- | --- | --- | --- |
|  | **x͂** | | **MWW** | |  | **x͂** | | | **Kruskal-Wallis** | | **Mood’s Median** | |
|  | R | R-E | W | *p* |  | L-E | E | R-E | H | *p* | Χ² | *p* |
| **1** | 0.000 | 0.000 | 9.0 | 0.136 |  | 0.500 | 0.500 | 0.550 | 1.783 | 0.410 | 2.100 | 0.350 |
| **2** | 0.000 | 0.000 | 9.0 | 0.136 |  | 0.500 | 0.500 | 0.550 | 1.783 | 0.410 | 2.100 | 0.350 |
| **3** | 0.130 | 0.170 | 5.0 | 0.141 |  | 0.400 | 0.130 | 0.090 | 1.056 | 0.590 | 1.533 | 0.465 |
| **4** | 0.790 | 0.700 | 13.5 | 0.459 |  | 0.630 | 0.625 | 0.865 | 1.384 | 0.501 | 2.261 | 0.323 |
| **5** | 0.925 | 0.800 | 25.0 | 0.082 |  | 0.630 | 0.890 | 0.875 | 0.891 | 0.640 | 0.533 | 0.766 |
| **6** | 0.000 | 0.000 | – | – |  | 0.500 | 0.500 | 0.500 | – | – | – | – |
| **7** | 1.000 | 1.000 | – | – |  | 0.500 | 0.500 | 0.500 | – | – | – | – |
| **8** | 0.170 | 0.060 | 18.0 | 0.287 |  | 0.500 | 0.500 | 0.520 | 1.590 | 0.452 | 2.177 | 0.337 |
| **9** | 0.830 | 0.500 | 13.0 | 1.000 |  | 0.900 | 1.000 | 0.855 | 1.600 | 0.449 | 3.133 | 0.209 |
| **10** | 1.000 | 1.000 | – | – |  | 0.500 | 0.500 | 0.500 | – | – | – | – |
| **11** | 0.000 | 0.000 | – | – |  | 0.500 | 0.500 | 0.500 | – | – | – | – |
| **12** | 1.000 | 1.000 | 12.5 | 0.465 |  | 1.000 | 1.000 | 1.000 | – | – | – | – |
| **13** | 0.000 | 0.000 | – | – |  | 0.500 | 0.500 | 0.500 | 1.400 | 0.497 | 1.527 | 0.466 |
| **14** | 0.000 | 0.000 | – | – |  | 0.500 | 0.500 | 0.500 | – | – | – | – |
| **15** | 0.000 | 0.000 | – | – |  | 0.500 | 0.500 | 0.500 | – | – | – | – |
| **16** | 0.250 | 0.080 | 16.5 | 0.458 |  | 0.500 | 0.500 | 0.585 | 2.684 | 0.261 | 2.400 | 0.301 |
| **17** | 0.130 | 0.000 | 16.0 | 0.921 |  | 0.500 | 0.620 | 0.585 | 3.257 | 0.196 | 4.800 | 0.091 |
| **18** | 1.000 | 1.000 | – | – |  | 0.500 | 0.500 | 0.500 | 1.750 | 0.417 | n.a. | n.a |
| **19** | 0.500 | 0.670 | 8.5 | 0.458 |  | 0.380 | 0.025 | 0.270 | 0.586 | 0.746 | 1.253 | 0.535 |
| **20** | 1.000 | 1.000 | – | – |  | 0.500 | 0.500 | 0.500 | – | – | – | – |
| **21** | 0.000 | 0.000 | – | – |  | 0.500 | 0.500 | 0.500 | – | – | – | – |
| **22** | 1.000 | 1.000 | 12.5 | 0.465 |  | 1.000 | 1.000 | 1.000 | – | – | – | – |
| **23** | 1.000 | 1.000 | – | – |  | 0.750 | 0.500 | 0.500 | **9.128** | **0.010** | **12.000** | **0.002** |
| **24** | 0.000 | 0.000 | 17.5 | 0.465 |  | 0.500 | 0.500 | 0.550 | **7.157** | **0.028** | **8.000** | **0.018** |
| **25** | 0.000 | 0.000 | 12.0 | 0.361 |  | 0.500 | 0.500 | 0.500 | – | – | – | – |
| **26** | 0.780 | 0.440 | 22.5 | 0.200 |  | 0.400 | 0.250 | 0.165 | 1.765 | 0.414 | 1.234 | 0.540 |
| **27** | 0.550 | 0.440 | 19.0 | 0.523 |  | 0.400 | 0.250 | 0.165 | 1.765 | 0.414 | 1.234 | 0.540 |
| **28** | 0.460 | 0.180 | 23.0 | 0.170 |  | 0.200 | 0.000 | 0.030 | **8.824** | **0.012** | **7.886** | **0.019** |
| **29** | 1.000 | 1.000 | – | – |  | 0.500 | 0.500 | 0.500 | – | – | – | – |
| **30** | 0.000 | 0.000 | 9.0 | 0.134 |  | 0.500 | 0.500 | 0.500 | – | – | – | – |
| **31** | 1.000 | 1.000 | 9.0 | 0.134 |  | 0.880 | 0.500 | 0.500 | **8.437** | **0.015** | **7.063** | **0.029** |
| **32** | 0.000 | 0.000 | 9.0 | 0.134 |  | 0.500 | 0.500 | 0.500 | – | – | – | – |
| **33** | 0.000 | 0.000 | – | – |  | 0.500 | 0.500 | 0.500 | – | – | – | – |
| **34** | 0.945 | 0.830 | 20.0 | 0.409 |  | 1.000 | 1.000 | 0.900 | 5.189 | 0.075 | n.a. | n.a. |
| **35** | 1.000 | 1.000 | – | – |  | 0.500 | 0.500 | 0.500 | – | – | – | – |
| **36** | 0.000 | 0.000 | – | – |  | 0.500 | 0.500 | 0.500 | – | – | – | – |
| **37** | 0.950 | 0.890 | 18.0 | 0.647 |  | 1.000 | 1.000 | 0.940 | 1.830 | 0.401 | n.a. | n.a. |
| **38** | 0.950 | 0.890 | 18.0 | 0.647 |  | 0.500 | 0.500 | 0.440 | 1.830 | 0.401 | n.a. | n.a. |
| **39** | 1.000 | 1.000 | – | – |  | 0.500 | 0.500 | 0.500 | – | – | – | – |
| **40** | 0.000 | 0.000 | – | – |  | 0.000 | 0.150 | 0.000 | 3.790 | 0.150 | 3.600 | 0.165 |
| **41** | 1.000 | 1.000 | – | – |  | 0.500 | 0.500 | 0.500 | – | – | – | – |
| **42** | 0.030 | 0.070 | 16.0 | 0.925 |  | 0.000 | 0.000 | 0.025 | 2.611 | 0.271 | 3.111 | 0.211 |
| **43** | 0.140 | 0.210 | 8.5 | 0.271 |  | 0.000 | 0.000 | 0.120 | 2.000 | 0.367 | 6.133 | 0.047 |
| **44** | 0.000 | 0.000 | – | – |  | 0.500 | 0.500 | 0.500 | 3.000 | 0.223 | n.a. | n.a. |
| **45** | 1.000 | 1.000 | – | – |  | 0.500 | 0.500 | 0.500 | 3.000 | 0.223 | 3.273 | 0.195 |
| **46** | 0.000 | 0.000 | – | – |  | 0.500 | 0.500 | 0.500 | – | – | – | – |
| **47** | 0.030 | 0.070 | 13.0 | 0.779 |  | 0.500 | 0.000 | 0.020 | **7.484** | **0.024** | 4.800 | 0.091 |
| **48** | 0.325 | 0.210 | 20.5 | 0.360 |  | 0.500 | 0.270 | 0.145 | **6.483** | **0.039** | **7.200** | **0.027** |
| **49** | 0.300 | 0.210 | 19.5 | 0.464 |  | 0.500 | 0.250 | 0.160 | **6.414** | **0.040** | **7.200** | **0.027** |
| **50** | 0.765 | 0.850 | 10.5 | 0.464 |  | 1.000 | 0.750 | 0.905 | 1.864 | 0.394 | 0.533 | 0.766 |
| **51** | 0.700 | 0.790 | 10.5 | 0.464 |  | 0.500 | 0.750 | 0.840 | **6.414** | **0.040** | **7.200** | **0.027** |
| **52** | 1.000 | 1.000 | – | – |  | 0.750 | 0.500 | 0.500 | **7.493** | **0.024** | **7.063** | **0.029** |
| **53** | 1.000 | 1.000 | – | – |  | 0.500 | 0.500 | 0.500 | – | – | – | – |
| **54** | 1.000 | 1.000 | – | – |  | 0.500 | 0.500 | 0.500 | – | – | – | – |
| **55** | 1.000 | 1.000 | – | – |  | 0.500 | 0.500 | 0.500 | – | – | – | – |
| **56** | 1.000 | 1.000 | – | – |  | 0.500 | 0.500 | 0.500 | – | – | – | – |
| **57** | 0.000 | 0.000 | 15.0 | 0.916 |  | 0.500 | 0.500 | 0.525 | 4.716 | 0.095 | 4.800 | 0.091 |
| **58** | 0.000 | 0.000 | – | – |  | 0.500 | 0.500 | 0.500 | – | – | – | – |
| **59** | 1.000 | 1.000 | – | – |  | 1.000 | 1.000 | 1.000 | 3.000 | 0.223 | n.a. | n.a. |
| **60** | 0.580 | 0.530 | 22.0 | 0.231 |  | 0.000 | 0.100 | 0.220 | 3.713 | 0.156 | 3.977 | 0.137 |
| **61** | 0.000 | 0.000 | 17.5 | 0.465 |  | 0.500 | 0.500 | 0.500 | – | – | – | – |
| **62** | 1.000 | 1.000 | – | – |  | 0.500 | 0.500 | 0.500 | – | – | – | – |
| **63** | 0.000 | 0.000 | – | – |  | 0.500 | 0.500 | 0.500 | – | – | – |  |
| **64** | 0.450 | 0.140 | 21.0 | 0.315 |  | 1.000 | 0.670 | 0.740 | 4.629 | 0.099 | 4.800 | 0.091 |
| **65** | 0.000 | 0.000 | – | – |  | 0.500 | 0.500 | 0.500 | – | – | – | – |
| **66** | 0.140 | 0.120 | 16.5 | 0.855 |  | 0.500 | 0.500 | 0.500 | – | – | – | – |
| **67** | 1.000 | 1.000 | – | – |  | 0.500 | 0.500 | 0.500 | 1.431 | 0.489 | n.a. | n.a. |
| **68** | 0.000 | 0.000 | – | – |  | 0.500 | 0.500 | 0.500 | – | – | – | – |
| **69** | 0.230 | 0.310 | 17.0 | 0.784 |  | 0.500 | 0.760 | 0.575 | 1.871 | 0.393 | 0.533 | 0.766 |
| **70** | 1.000 | 1.000 | – | – |  | 0.500 | 0.500 | 0.500 | 1.750 | 0.417 | 1.925 | 0.382 |
| **71** | 1.000 | 1.000 | – | – |  | 0.500 | 0.500 | 0.500 | 1.750 | 0.417 | 1.925 | 0.382 |
| **72** | 1.000 | 1.000 | – | – |  | 0.500 | 0.500 | 0.500 | – | – | – | – |
| **73** | 0.290 | 0.190 | 16.5 | 0.855 |  | 0.500 | 0.900 | 0.770 | 2.543 | 0.280 | 0.533 | 0.766 |
| **74** | 0.880 | 0.850 | 14.0 | 0.926 |  | 0.830 | 0.500 | 0.380 | **7.381** | **0.025** | **7.200** | **0.027** |
| **75** | 0.765 | 0.910 | 7.5 | 0.200 |  | 0.500 | 0.390 | 0.415 | 4.069 | 0.131 | 4.200 | 0.123 |
| **76** | 0.570 | 0.270 | 24.0 | 0.121 |  | 0.500 | 0.610 | 0.665 | 2.311 | 0.315 | 1.533 | 0.465 |
| **77** | 1.000 | 1.000 | 12.5 | 0.465 |  | 1.000 | 1.000 | 1.000 | – | – | – | – |
| **78** | 1.000 | 1.000 | 12.5 | 0.465 |  | 0.500 | 0.500 | 0.500 | – | – | – | – |
| **79** | 0.000 | 0.000 | – | – |  | 0.500 | 0.500 | 0.500 | – | – | – | – |
| **80** | 0.725 | 0.730 | 15.5 | 1.000 |  | 0.100 | 0.460 | 0.370 | 1.758 | 0.415 | 3.133 | 0.209 |
| **81** | 0.670 | 0.480 | 23.0 | 0.170 |  | 0.700 | 0.000 | 0.125 | **7.731** | **0.021** | 4.800 | 0.091 |
| **82** | 0.240 | 0.290 | 18.0 | 0.640 |  | 0.000 | 0.000 | 0.050 | 4.364 | 0.113 | 4.800 | 0.091 |
| **83** | 1.000 | 1.000 | – | – |  | 0.880 | 0.750 | 0.500 | 4.476 | 0.107 | **7.200** | **0.027** |
| **84** | 1.000 | 1.000 | – | – |  | 0.500 | 0.500 | 0.500 | 1.400 | 0.497 | n.a. | n.a. |
| **85** | 1.000 | 1.000 | – | – |  | 0.500 | 0.500 | 0.500 | – |  | – |  |
| **86** | 0.465 | 0.820 | 5.0 | 0.083 |  | 0.300 | 0.420 | 0.315 | 1.805 | 0.406 | 0.533 | 0.766 |
| **87** | 1.000 | 1.000 | – | – |  | 0.500 | 0.500 | 0.500 | – | – | – | – |
| **88** | 0.000 | 0.000 | – | – |  | 0.500 | 0.500 | 0.500 | – | – | – | – |
| **89** | 0.905 | 1.000 | 13.5 | 0.852 |  | 1.000 | 0.630 | 0.825 | **7.082** | **0.029** | 4.800 | 0.091 |
| **90** | 0.000 | 0.000 | – | – |  | 0.500 | 0.500 | 0.500 | – | – | – | – |
| **91** | 1.000 | 1.000 | – | – |  | 0.500 | 0.500 | 0.500 | – | – | – | – |
| **92** | 1.000 | 1.000 | – | – |  | 0.500 | 0.500 | 0.500 | – | – | – | – |
| **93** | 1.000 | 1.000 | – | – |  | 0.500 | 0.500 | 0.500 | – | – | – | – |
| **94** | 0.615 | 0.710 | 16.0 | 0.927 |  | 0.670 | 0.500 | 0.725 | 4.751 | 0.093 | 3.133 | 0.209 |
| **95** | 0.200 | 0.310 | 16.5 | 0.855 |  | 0.000 | 0.000 | 0.025 | 2.783 | 0.249 | 3.111 | 0.211 |
| **96** | 0.020 | 0.000 | 22.5 | 0.104 |  | 0.000 | 0.000 | 0.000 | 3.000 | 0.223 | 3.273 | 0.195 |
| **97** | 1.000 | 0.960 | 18.0 | 0.619 |  | 0.500 | 0.500 | 0.500 | 1.431 | 0.489 | n.a. | n.a. |
| **98** | 0.000 | 0.000 | – | – |  | 0.500 | 0.500 | 0.500 | – | – | – | – |
| **99** | 0.000 | 0.000 | 17.5 | 0.465 |  | 0.500 | 0.500 | 0.500 | – | – | – | – |
| **100** | 1.000 | 1.000 | 10.0 | 0.223 |  | 0.500 | 0.500 | 0.500 | 3.000 | 0.223 | 3.273 | 0.195 |
| **101** | 1.000 | 1.000 | 16.0 | 0.916 |  | 1.000 | 0.800 | 1.000 | 5.137 | 0.077 | 3.133 | 0.209 |
| **102** | 0.030 | 0.000 | 21.0 | 0.245 |  | 0.500 | 0.500 | 0.500 | – | – | – | – |
| **103** | 1.000 | 1.000 | 10.0 | 0.223 |  | 0.500 | 0.500 | 0.500 | – | – | – | – |
| **104** | 0.630 | 0.550 | 19.0 | 0.523 |  | 0.750 | 0.900 | 0.735 | 2.939 | 0.230 | 3.133 | 0.209 |
| **105** | 1.000 | 1.000 | – | – |  | 1.000 | 1.000 | 1.000 | 1.829 | 0.401 | n.a. | n.a. |
| **106** | 0.310 | 0.140 | 23.0 | 0.170 |  | 0.500 | 0.500 | 0.535 | 4.716 | 0.095 | 4.800 | 0.091 |
| **107** | 0.000 | 0.000 | 17.5 | 0.465 |  | 0.500 | 0.500 | 0.500 | 3.000 | 0.223 | n.a. | n.a. |
| **108** | 1.000 | 1.000 | – | – |  | 0.500 | 0.500 | 0.500 | 1.804 | 0.406 | n.a. | n.a. |
| **109** | 0.670 | 0.560 | 14.0 | 0.834 |  | 0.800 | 0.630 | 0.810 | 3.048 | 0.218 | 3.133 | 0.209 |
| **110** | 0.000 | 0.000 | – | – |  | 0.500 | 0.500 | 0.500 | – | – | – | – |
| **111** | 0.810 | 0.880 | 9.5 | 0.360 |  | 1.000 | 1.000 | 0.920 | **7.748** | **0.021** | n.a. | n.a. |
| **112** | 1.000 | 1.000 | – | – |  | 0.500 | 0.500 | 0.500 | – | – | – | – |
| **113** | 0.275 | 0.500 | 8.0 | 0.235 |  | 0.500 | 1.000 | 0.845 | **9.469** | **0.009** | **9.000** | **0.011** |
| **114** | 0.000 | 0.000 | 17.5 | 0.465 |  | 0.500 | 0.500 | 0.500 | – | – | – | – |
| **115** | 1.000 | 1.000 | – | – |  | 0.500 | 0.500 | 0.500 | – | – | – | – |
| **116** | 0.500 | 0.850 | 3.0 | 0.060 |  | 0.500 | 0.450 | 0.410 | 0.586 | 0.746 | 1.253 | 0.535 |
| **117** | 0.000 | 0.000 | – | – |  | 0.500 | 0.500 | 0.500 | – | – | – | – |
| **118** | 0.000 | 0.000 | – | – |  | 0.250 | 0.500 | 0.500 | **10.730** | **0.005** | n.a. | n.a. |
| **119** | 1.000 | 1.000 | – | – |  | 0.500 | 0.500 | 0.500 | – | – | – |  |
| **120** | 0.570 | 0.820 | 7.0 | 0.170 |  | 0.400 | 0.500 | 0.395 | 2.270 | 0.321 | 3.600 | 0.165 |
| **121** | 0.000 | 0.000 | – | – |  | 0.500 | 0.500 | 0.500 | – | – | – | – |
| **122** | 1.000 | 1.000 | 18.0 | 0.361 |  | 0.580 | 0.500 | 0.500 | 5.776 | 0.056 | **7.200** | **0.027** |
| **123** | 0.895 | 1.000 | 7.5 | 0.103 |  | 0.500 | 0.500 | 0.500 | – | – | – | – |
| **124** | 0.000 | 0.000 | 20.0 | 0.223 |  | 0.500 | 0.500 | 0.500 | – | – | – | – |
| **125** | 0.000 | 0.000 | 20.0 | 0.223 |  | 0.500 | 0.500 | 0.500 | – | – | – | – |
| **126** | 1.000 | 1.000 | – | – |  | 0.500 | 0.500 | 0.500 | – | – | – | – |
| **127** | 1.000 | 1.000 | – | – |  | 0.500 | 0.500 | 0.500 | – | – | – | – |
| **128** | 0.000 | 0.000 | – | – |  | 0.500 | 0.500 | 0.500 | – | – | – | – |
| **129** | 1.000 | 1.000 | 12.5 | 0.465 |  | 0.500 | 0.500 | 0.500 | 1.335 | 0.513 | n.a. | n.a. |
| **130** | 1.000 | 1.000 | 21.0 | 0.136 |  | 0.500 | 0.500 | 0.500 | 2.000 | 0.368 | n.a. | n.a. |
| **131** | 0.000 | 0.000 | 9.0 | 0.136 |  | 0.500 | 0.500 | 0.500 | 2.000 | 0.368 | 2.182 | 0.336 |

**Table S8.** Results of logistic regression using frequencies of single nucleotide polymorphisms (SNPs) of diploid *P. esculentus* (genotype LR) as dependent variables. Predictor variables included population system (POPSYS), longitude (LON), and latitude (LAT). Only SNPs significantly affected by one or more predictors, as indicated by type III sum of squares statistics, are shown. Model (M) variants: a – all independent variables included; f – forward selection; b – backward selection. R² represents the proportion of variability in SNP frequencies explained by the regression model; R²_adj_ allows comparisons between models with different numbers of coefficients. In some cases, the logistic function did not fit the observed data (Χ² < 0.05), or the goodness-of-fit test could not be performed due to insufficient data (n.a.). System-specific median differences in SNP frequencies were evaluated using Kruskal-Wallis (K-W) tests; significant differences are highlighted in **bold**. L-E: *lessonae*-*esculentus* populations; E: all-hybrid (*esculentus*) populations; R-E: *ridibundus*-*esculentus* populations.

| **Gene** | **Exon** | **SNP** | **Logistic regression (Weighted Least Squares)** | | | | | | | | | | **Median** | | | **K-W** | |
| --- | --- | --- | --- | --- | --- | --- | --- | --- | --- | --- | --- | --- | --- | --- | --- | --- | --- |
|  |  |  | **Model (Analysis of Variance)** | | | | | | **Type III Sum of Squares** | | | | **POPSYS** | | |  |  |
|  |  |  | **M** | **F** | ***p*** | **R²** | **R²_adj_** | **Χ²** | **Variable** | **F** | **d.f.** | ***p*** | **L-E** | **E** | **R-E** | **H** | ***p*** |
| *brd2* | 2 | 3 | f,b | 7.49 | 0.012 | 62.5 | 54.1 | n.a. | POPSYS | 7.49 | 1 | 0.012 | 0.500 | 0.500 | 0.550 | 1.783 | 0.410 |
|  | 4 | 4 | f,b | 33.32 | <0.001 | 93.5 | 90.6 | 0.129 | LON | 12.61 | 1 | 0.009 | 0.630 | 0.625 | 0.865 | 1.38 | 0.500 |
|  |  |  |  |  |  |  |  |  | POPSYS | 40.39 | 2 | <0.001 |  |  |  |  |  |
|  | 5 | 5 | f,b | 5.50 | 0.028 | 55.0 | 45.0 | n.a. | POPSYS | 5.50 | 2 | 0.028 | 0.630 | 0.890 | 0.875 | 0.89 | 0.640 |
| *camk2g* | 19 | 9 | b | 11.18 | 0.003 | 80.7 | 73.5 | 0.373 | LAT | 25.19 | 1 | 0.001 | 0.900 | 1.000 | 0.855 | 1.60 | 0.449 |
|  |  |  |  |  |  |  |  |  | POPSYS | 16.75 | 2 | 0.001 |  |  |  |  |  |
| *fbxo5* | 1 | 23 | a | 13.74 | 0.002 | 88.7 | 82.2 | 0.956 | POPSYS | 14.58 | 2 | 0.003 | 0.750 | 0.500 | 0.500 | **9.13** | **0.010** |
| *hat1* | 9 | 24 | f,b | 5.24 | 0.031 | 53.8 | 43.6 | n.a. | POPSYS | 5.24 | 2 | 0.031 | 0.500 | 0.500 | 0.550 | **7.16** | **0.028** |
| *henmt* | 6 | 28 | a | 4.58 | 0.039 | 72.4 | 56.6 | 0.0 | LAT | 5.82 | 1 | 0.047 | 0.200 | 0.000 | 0.030 | **8.82** | **0.012** |
|  |  |  |  |  |  |  |  |  | POPSYS | 8.49 | 2 | 0.013 |  |  |  |  |  |
| *hormad1* | 7 | 31 | a,b | 33.62 | <0.001 | 95.0 | 92.2 | 0.992 | LAT | 9.09 | 1 | 0.020 | 0.880 | 0.500 | 0.500 | **8.44** | **0.015** |
|  |  |  |  |  |  |  |  |  | LON | 15.17 | 1 | 0.006 |  |  |  |  |  |
|  |  |  |  |  |  |  |  |  | POPSYS | 45.02 | 2 | <0.001 |  |  |  |  |  |
| *igf1r* | 6 | 34 | f,b | 41.89 | <0.001 | 94.0 | 91.8 | 0.840 | LON | 103.67 | 1 | <0.001 | 1.000 | 1.000 | 0.900 | 5.19 | 0.075 |
|  |  |  |  |  |  |  |  |  | POPSYS | 20.77 | 2 | <0.001 |  |  |  |  |  |
| *itpr1* | 5 | 37 | a,f,b | 66.62 | <0.001 | 97.4 | 96.0 | <0.001 | LAT | 84.63 | 1 | <0.001 | 1.000 | 1.000 | 0.940 | 1.83 | 0.401 |
|  |  |  |  |  |  |  |  |  | LON | 250.01 | 1 | <0.001 |  |  |  |  |  |
|  |  |  |  |  |  |  |  |  | POPSYS | 16.53 | 2 | 0.002 |  |  |  |  |  |
|  | 21 | 42 | a,b | 123.45 | <0.001 | 98.6 | 97.8 | 0.951 | LAT | 8.14 | 1 | 0.025 | 0.000 | 0.000 | 0.025 | 2.61 | 0.271 |
|  |  |  |  |  |  |  |  |  | LON | 10.90 | 1 | 0.013 |  |  |  |  |  |
|  |  |  |  |  |  |  |  |  | POPSYS | 17.90 | 2 | 0.002 |  |  |  |  |  |
|  |  | 44 | f,b | 9.14 | 0.007 | 67.0 | 59.7 | 0.953 | LAT | 17.31 | 1 | 0.002 | 0.500 | 0.500 | 0.500 | 3.00 | 0.223 |
|  |  |  |  |  |  |  |  |  | LON | 8.48 | 1 | 0.017 |  |  |  |  |  |
|  | 29 | 45 | f,b | 15.50 | 0.001 | 77.5 | 72.5 | 0.797 | LAT | 29.82 | 1 | <0.001 | 0.500 | 0.500 | 0.500 | 3.00 | 0.223 |
|  |  |  |  |  |  |  |  |  | LON | 15.50 | 1 | 0.003 |  |  |  |  |  |
| *kif22* | 2 | 47 | f,b | 42.31 | <0.001 | 90.4 | 88.2 | 0.099 | POPSYS | 42.31 | 2 | <0.001 | 0.500 | 0.000 | 0.020 | **7.48** | **0.024** |
|  | 5 | 48 | a | 6.55 | 0.016 | 78.9 | 66.9 | 0.114 | POPSYS | 10.73 | 2 | 0.007 | 0.500 | 0.270 | 0.145 | **6.48** | **0.039** |
|  |  | 49 | a | 6.64 | 0.016 | 79.1 | 67.2 | 0.011 | POPSYS | 10.10 | 2 | 0.009 | 0.500 | 0.250 | 0.160 | **6.41** | **0.040** |
|  | 6 | 50 | a,b | 221.83 | <0.001 | 99.2 | 98.8 | 0.016 | LAT | 198.84 | 1 | <0.001 | 1.000 | 0.750 | 0.905 | 1.86 | 0.394 |
|  |  |  |  |  |  |  |  |  | LON | 214.11 | 1 | <0.001 |  |  |  |  |  |
|  |  |  |  |  |  |  |  |  | POPSYS | 198.45 | 2 | <0.001 |  |  |  |  |  |
|  |  |  | f | 13.60 | 0.002 | 75.1 | 69.6 | 0.054 | POPSYS | 13.60 | 2 | 0.002 |  |  |  |  |  |
|  |  | 51 | a | 6.64 | 0.016 | 79.2 | 67.2 | 0.222 | POPSYS | 10.10 | 2 | 0.009 | 0.500 | 0.750 | 0.840 | **6.41** | **0.040** |
| *kmt2a* | 7 | 52 | a | 1.50 | 0.298 | 46.2 | 15.5 | 0.547 | – | – | – | – | 0.750 | 0.500 | 0.500 | **7.49** | **0.024** |
| *m1ap* | 1 | 57 | b | 6.68 | 0.014 | 71.5 | 60.8 | 0.990 | LAT | 10.38 | 1 | 0.012 | 0.500 | 0.500 | 0.525 | 4.72 | 0.095 |
|  |  |  |  |  |  |  |  |  | POPSYS | 5.58 | 2 | 0.030 |  |  |  |  |  |
| *map2k1* | 3 | 59 | f,b | 103.43 | <0.001 | 95.8 | 94.9 | 1.000 | LAT | 131.47 | 1 | <0.001 | 1.000 | 1.000 | 1.000 | 3.00 | 0.223 |
|  |  |  |  |  |  |  |  |  | LON | 10.28 | 1 | 0.011 |  |  |  |  |  |
| *mns1* | 7 | 74 | a | 3.38 | 0.077 | 65.9 | 46.4 | 0.830 | POPSYS | 5.52 | 2 | 0.036 | 0.830 | 0.500 | 0.380 | **7.38** | **0.025** |
|  |  |  | f,b | 8.57 | 0.008 | 65.6 | 57.9 | n.a. | POPSYS | 8.57 | 2 | 0.008 |  |  |  |  |  |
| *nibrin* | 10 | 80 | f,b | 8.88 | 0.014 | 47.0 | 41.7 | 0.592 | LON | 8.88 | 1 | 0.014 | 0.100 | 0.460 | 0.370 | 1.76 | 0.415 |
| *nusap1* | 8 | 81 | f,b | 48.93 | <0.001 | 94.8 | 92.9 | 0.344 | LAT | 11.15 | 1 | 0.010 | 0.700 | 0.000 | 0.125 | **7.73** | **0.021** |
|  |  |  |  |  |  |  |  |  | POPSYS | 20.37 | 2 | <0.001 |  |  |  |  |  |
|  |  | 82 | f,b | 44.36 | <0.001 | 94.3 | 92.2 | n.a | LAT | 115.34 | 1 | <0.001 | 0.000 | 0.000 | 0.050 | 4.36 | 0.113 |
|  |  |  |  |  |  |  |  |  | POPSYS | 11.67 | 2 | 0.004 |  |  |  |  |  |
| *piwil2* | 12 | 89 | f,b | 11.13 | 0.008 | 52.7 | 47.9 | 0.042 | LON | 11.13 | 1 | 0.008 | 1.000 | 0.630 | 0.825 | **7.08** | **0.029** |
|  |  |  |  |  |  |  |  |  |  |  |  |  |  |  |  |  |  |
| *pkmyt1* | 5 | 94 | f,b | 6.85 | 0.026 | 40.7 | 34.7 | n.a. | LAT | 6.85 | 1 | 0.026 | 0.670 | 0.500 | 0.725 | 4.75 | 0.093 |
|  |  | 95 | b | 20.10 | <0.001 | 88.3 | 83.9 | n.a. | LAT | 46.38 | 1 | <0.001 | 0.000 | 0.000 | 0.025 | 2.78 | 0.249 |
|  |  |  |  |  |  |  |  |  | POPSYS | 26.10 | 2 | <0.001 |  |  |  |  |  |
| *plk1* | 1 | 96 | f,b | 108.72 | <0.001 | 96.0 | 95.1 | 0.958 | LAT | 138.87 | 1 | <0.001 | 0.000 | 0.000 | 0.000 | 3.00 | 0.223 |
|  |  |  |  |  |  |  |  |  | LON | 11.12 | 1 | 0.009 |  |  |  |  |  |
|  |  |  |  |  |  |  |  |  |  |  |  |  |  |  |  |  |  |
| *ppp1ca* | 3 | 100 | f,b | 10.65 | 0.004 | 70.3 | 63.7 | 0.760 | LAT | 20.21 | 1 | 0.002 | 0.500 | 0.500 | 0.500 | 3.00 | 0.223 |
|  |  |  |  |  |  |  |  |  | LON | 9.91 | 1 | 0.012 |  |  |  |  |  |
|  |  | 101 | a,b | 50.13 | <0.001 | 96.6 | 94.7 | n.a. | LAT | 65.94 | 1 | <0.001 | 1.000 | 0.800 | 1.000 | 5.14 | 0.077 |
|  |  |  |  |  |  |  |  |  | LON | 5.69 | 1 | 0.048 |  |  |  |  |  |
|  |  |  |  |  |  |  |  |  | POPSYS | 29.88 | 2 | <0.001 |  |  |  |  |  |
|  |  |  | f | 18.12 | 0.002 | 64.4 | 60.9 | 0.007 | LON | 18.12 | 1 | 0.002 |  |  |  |  |  |
|  |  | 104 | b | 4.39 | 0.042 | 62.2 | 48.1 | n.a. | LON | 12.56 | 1 | 0.008 | 0.750 | 0.900 | 0.735 | 2.94 | 0.230 |
|  |  |  |  |  |  |  |  |  | POPSYS | 5.07 | 2 | 0.038 |  |  |  |  |  |
| *ppp2r1a* | 4 | 105 | b | 10.27 | 0.004 | 79.4 | 71.7 | n.a. | LON | 29.98 | 1 | <0.001 | 1.000 | 1.000 | 1.000 | 1.83 | 0.401 |
|  |  |  |  |  |  |  |  |  | POPSYS | 15.02 | 2 | 0.002 |  |  |  |  |  |
|  |  | 106 | b | 6.94 | 0.013 | 72.2 | 61.8 | n.a. | LAT | 9.23 | 1 | 0.016 | 0.500 | 0.535 | 0.500 | 4.72 | 0.095 |
|  |  |  |  |  |  |  |  |  | POPSYS | 8.93 | 2 | 0.009 |  |  |  |  |  |
| *ppp3ca* | 2 | 109 | b | 28.08 | <0.001 | 73.7 | 71.1 | 0.363 | LON | 28.08 | 1 | <0.001 | 0.800 | 0.630 | 0.810 | 3.04 | 0.218 |
| *rad50* | 5 | 111 | a | 44.64 | <0.001 | 96.2 | 94.1 | 0.979 | POPSYS | 10.57 | 2 | 0.008 | 1.000 | 1.000 | 0.920 | **7.75** | **0.021** |
|  |  |  | f | 41.42 | <0.001 | 94.0 | 91.7 | 0.914 | LON | 109.96 | 1 | <0.001 |  |  |  |  |  |
|  |  |  |  |  |  |  |  |  | POPSYS | 6.51 | 2 | 0.021 |  |  |  |  |  |
|  |  |  | b | 63.32 | <0.001 | 96.0 | 94.4 | 0.987 | LAT | 168.56 | 1 | <0.001 |  |  |  |  |  |
|  |  |  |  |  |  |  |  |  | POPSYS | 65.77 | 2 | <0.001 |  |  |  |  |  |
| *rad51ap1* | 5 | 113 | a | 5.90 | 0.021 | 77.1 | 64.0 | 0.996 | LON | 6.97 | 1 | 0.033 | 0.500 | 1.000 | 0.845 | **9.47** | **0.009** |
|  |  |  | f,b | 6.82 | 0.014 | 71.9 | 61.4 | 0.945 | LON | 6.10 | 1 | 0.039 |  |  |  |  |  |
|  |  |  |  |  |  |  |  |  | POPSYS | 6.75 | 2 | 0.019 |  |  |  |  |  |
| *sfr1* | 2 | 118 | a | 11.80 | 0.003 | 87.1 | 79.7 | 1.000 | POPSYS | 12.81 | 2 | 0.005 | 0.250 | 0.500 | 0.500 | **10.73** | **0.005** |
|  |  | 120 | f,b | 6.95 | 0.025 | 41.0 | 35.1 | 0.894 | LAT | 6.95 | 1 | 0.025 | 0.400 | 0.500 | 0.395 | 2.27 | 0.321 |
| *sgol1* | 2 | 122 | f,b | 5.17 | 0.032 | 53.5 | 43.1 | n.a. | POPSYS | 5.17 | 2 | 0.032 | 0.580 | 0.500 | 0.500 | 5.78 | 0.056 |

**Table S9.** Results of logistic regression using the frequencies of single nucleotide polymorphisms (SNPs) of *P. ridibundus* (genotype RR) as dependent variables. The predictor variables included population system (POPSYS), longitude (LON), and latitude (LAT). Only SNPs significantly affected by one or more predictors, as indicated by type III sums of squares statistics, are shown. Model (M) variants: a – all independent variables included, f – forward selection, b – backward selection. R² represents the proportion of variability in SNP frequencies explained by the regression model; R²_adj_ allows comparisons between models with different numbers of coefficients. In some cases, the logistic function did not fit the observed data (Χ² < 0.05), or the goodness-of-fit test could not be performed due to insufficient data (n.a.). System-specific median differences in SNP frequencies were evaluated using Mann-Whitney-Wilcoxon (MWW) tests; significant differences are highlighted in **bold**. R: all-*ridibundus* populations; R-E: *ridibundus*‑*esculentus* populations.

| **Gene** | **Exon** | **SNP** | **Logistic regression (Weighted Least Squares)** | | | | | | | | | | **Median** | | **MWW** | |
| --- | --- | --- | --- | --- | --- | --- | --- | --- | --- | --- | --- | --- | --- | --- | --- | --- |
|  |  |  | **Model (Analysis of Variance)** | | | | | | **Type III Sum of Squares** | | | | **POPSYS** | |  |  |
|  |  |  | **M** | **F** | **p** | **R²** | **R²_adj_** | **p (Χ²)** | **Variable** | **F** | **d.f.** | **p** | **R** | **R-E** | **W** | **p** |
| *adcy9* | 6 | 1 | f | 20.57 | 0.001 | 69.6 | 66.2 | 0.406 | LON | 20.57 | 1 | 0.001 | 0.0 | 0.0 | 9.0 | 0.136 |
|  |  |  | b | 14.66 | 0.002 | 78.6 | 73.2 | 0.530 | LAT | 12.71 | 1 | 0.007 |  |  |  |  |
|  |  |  |  |  |  |  |  |  | POPSYS | 13.01 | 1 | 0.007 |  |  |  |  |
|  |  | 2 | f | 20.57 | 0.001 | 69.6 | 66.2 | 0.406 | LON | 20.57 | 1 | 0.001 | 0.0 | 0.0 | 9.0 | 0.136 |
|  |  |  | b | 14.66 | 0.002 | 78.6 | 73.2 | 0.530 | LAT | 12.71 | 1 | 0.007 |  |  |  |  |
|  |  |  |  |  |  |  |  |  | POPSYS | 13.01 | 1 | 0.007 |  |  |  |  |
| *brd2* | 2 | 3 | f | 9.21 | 0.016 | 53.5 | 47.7 | 0.598 | LON | 9.21 | 1 | 0.016 | 0.13 | 0.17 | 5.0 | 0.141 |
|  | 5 | 5 | f | 9.18 | 0.014 | 50.5 | 45.0 | 0.142 | LON | 9.18 | 1 | 0.014 | 0.92 | 0.80 | 25.0 | 0.082 |
| *camk2g* | 19 | 9 | a | 72.3 | <0.001 | 97.3 | 96.0 | <0.001 | LAT | 75.55 | 1 | <0.001 | 0.83 | 0.50 | 13.0 | 1.0 |
|  |  |  |  |  |  |  |  |  | LON | 51.08 | 1 | <0.001 |  |  |  |  |
|  |  |  |  |  |  |  |  |  | POPSYS | 150.19 | 1 | <0.001 |  |  |  |  |
| *cdc20* | 1 | 12 | f | 97.11 | <0.001 | 91.5 | 90.6 | 0.949 | LAT | 97.11 | 1 | <0.001 | 1.0 | 1.0 | 12.5 | 0.465 |
| *fbxo43* | 5 | 22 | a | 92.40 | <0.001 | 97.5 | 96.5 | 0.938 | LAT | 18.83 | 1 | 0.003 | 1.0 | 1.0 | 12.5 | 0.465 |
|  |  |  |  |  |  |  |  |  | LON | 57.36 | 1 | <0.001 |  |  |  |  |
|  |  |  |  |  |  |  |  |  | POPSYS | 9.97 | 1 | 0.016 |  |  |  |  |
| *hat1* | 9 | 24 | f | 18.14 | 0.001 | 81.9 | 77.4 | 0.987 | LON | 13.14 | 1 | 0.007 | 0.0 | 0.0 | 17.5 | 0.465 |
|  |  |  |  |  |  |  |  |  | POPSYS | 13.32 | 1 | 0.006 |  |  |  |  |
| *henmt1* | 3 | 25 | f | 13.18 | 0.006 | 59.4 | 54.9 | n.a. | POPSYS | 13.18 | 1 | 0.006 | 0.0 | 0.0 | 12.0 | 0.361 |
|  | 4 | 27 | b | 4.10 | 0.059 | 50.6 | 38.3 | 0.698 | LAT | 5.33 | 1 | <0.050 | 0.55 | 0.44 | 19.0 | 0.523 |
|  |  |  |  |  |  |  |  |  | POPSYS | 7.11 | 1 | 0.028 |  |  |  |  |
| *hormad1* | 7 | 30 | a | 4.69 | 0.042 | 66.8 | 52.5 | 0.351 | POPSYS | 6.18 | 1 | 0.042 | 0.0 | 0.0 | 9.0 | 0.134 |
|  |  | 31 | a | 4.69 | 0.042 | 66.8 | 52.5 | 0.432 | POPSYS | 6.18 | 1 | 0.042 | 1.0 | 1.0 | 21.0 | 0.134 |
|  |  | 32 | a | 4.69 | 0.042 | 66.8 | 52.5 | 0.351 | POPSYS | 6.18 | 1 | 0.042 | 0.0 | 0.0 | 9.0 | 0.134 |
| *igf1r* | 6 | 34 | b | 10.18 | 0.006 | 71.8 | 64.7 | 0.003 | LAT | 10.47 | 1 | 0.012 | 0.94 | 0.83 | 20.0 | 0.409 |
|  |  |  |  |  |  |  |  |  | POPSYS | 19.84 | 1 | 0.002 |  |  |  |  |
|  | 21 | 42 | f | 9.87 | 0.007 | 71.2 | 63.9 | 0.008 | LON | 17.12 | 1 | 0.003 | 0.03 | 0.07 | 16.0 | 0.925 |
|  |  |  |  |  |  |  |  |  | POPSYS | 5.98 | 1 | 0.040 |  |  |  |  |
|  |  | 43 | a | 5.98 | 0.024 | 71.9 | 59.9 | 0.515 | POPSYS | 9.96 | 1 | 0.016 | 0.14 | 0.21 | 8.5 | 0.271 |
|  |  |  | b | 8.62 | 0.010 | 68.3 | 60.4 | 0.841 | LAT | 12.39 | 1 | 0.008 |  |  |  |  |
|  |  |  |  |  |  |  |  |  | POPSYS | 14.45 | 1 | 0.005 |  |  |  |  |
| *m1ap* | 1 | 57 | a | 60.36 | <0.001 | 96.3 | 94.7 | <0.001 | LAT | 34.95 | 1 | <0.001 | 0.0 | 0.0 | 15.0 | 0.916 |
|  |  |  |  |  |  |  |  |  | LON | 29.40 | 1 | 0.001 |  |  |  |  |
|  |  |  |  |  |  |  |  |  | POPSYS | 12.29 | 1 | 0.010 |  |  |  |  |
| *map3k7* | 17 | 61 | f | 18.25 | 0.001 | 82.0 | 77.5 | 0.992 | LON | 13.25 | 1 | 0.007 | 0.0 | 0.0 | 17.5 | 0.465 |
|  |  |  |  |  |  |  |  |  | POPSYS | 13.36 | 1 | 0.006 |  |  |  |  |
| *mr11a* | 14 | 77 | f,b | 90.44 | <0.001 | 90.9 | 89.9 | 0.972 | LAT | 90.44 | 1 | <0.001 | 1.0 | 1.0 | 12.5 | 0.465 |
| *msh2* | 3 | 78 | a,b | 94.07 | <0.001 | 97.6 | 96.5 | 0.998 | LAT | 19.08 | 1 | 0.003 | 0.95 | 1.0 | 12.5 | 0.465 |
|  |  |  |  |  |  |  |  |  | LON | 58.36 | 1 | <0.001 |  |  |  |  |
|  |  |  |  |  |  |  |  |  | POPSYS | 9.98 | 1 | 0.016 |  |  |  |  |
|  |  |  | f | 90.36 | <0.001 | 90.0 | 89.9 | 0.756 | LON | 90.36 | 1 | <0.001 |  |  |  |  |
| *nusap1* | 8 | 81 | f | 9.05 | 0.015 | 50.2 | 44.6 | n.a. | LON | 9.05 | 1 | 0.015 | 0.67 | 0.48 | 23.0 | 0.170 |
|  |  | 82 | a | 14.05 | 0.002 | 85.8 | 79.6 | <0.001 | LAT | 11.12 | 1 | 0.012 | 0.24 | 0.29 | 18.0 | 0.640 |
|  |  |  |  |  |  |  |  |  | LON | 23.79 | 1 | 0.002 |  |  |  |  |
|  |  |  |  |  |  |  |  |  | POPSYS | 19.72 | 1 | 0.003 |  |  |  |  |
|  |  |  | f | 7.54 | 0.023 | 45.6 | 39.5 | n.a. | LON | 7.54 | 1 | 0.023 |  |  |  |  |
| *pelp1* | 4 | 86 | f | 8.34 | 0.018 | 48.1 | 42.3 | 0.073 | LAT | 8.34 | 1 | 0.018 | 0.44 | 0.82 | 5.0 | 0.083 |
|  |  |  | b | 7.60 | 0.022 | 45.8 | 39.8 | 0.154 | LON | 7.60 | 1 | 0.022 |  |  |  |  |
| *piwil2* | 12 | 89 | a | 8.16 | 0.011 | 77.8 | 68.2 | <0.001 | POPSYS | 8.01 | 1 | 0.025 | 0.90 | 1.00 | 13.5 | 0.952 |
|  |  |  | f,b | 31.15 | <0.001 | 77.6 | 75.1 | n.a. | POPSYS | 31.15 | 1 | <0.001 |  |  |  |  |
| *plk1* | 1 | 96 | a | 195.15 | <0.001 | 98.8 | 98.3 | 0.473 | LAT | 73.06 | 1 | <0.001 | 0.02 | 0.0 | 22.5 | 0.104 |
|  |  |  |  |  |  |  |  |  | LON | 404.42 | 1 | <0.001 |  |  |  |  |
|  |  |  |  |  |  |  |  |  | POPSYS | 12.66 | 1 | 0.009 |  |  |  |  |
|  |  | 97 | a | 34.14 | <0.001 | 93.6 | 90.9 | <0.001 | LAT | 43.09 | 1 | <0.001 | 1.0 | 0.96 | 18.0 | 0.619 |
|  |  |  |  |  |  |  |  |  | LON | 70.22 | 1 | <0.001 |  |  |  |  |
|  |  |  |  |  |  |  |  |  | POPSYS | 22.29 | 1 | 0.002 |  |  |  |  |
|  | 5 | 99 | f,b | 93.19 | <0.001 | 91.2 | 90.2 | 0.937 | LAT | 93.19 | 1 | <0.001 | 0.0 | 0.0 | 17.5 | 0.465 |
|  |  | 100 | b | 103.26 | <0.001 | 96.3 | 95.3 | 0.950 | LAT | 22.45 | 1 | 0.002 | 0.97 | 1.0 | 10.0 | 0.223 |
|  |  |  |  |  |  |  |  |  | LON | 29.95 | 1 | <0.001 |  |  |  |  |
| *ppp1ca* | 3 | 101 | a,f,b | 70.79 | <0.001 | 96.8 | 95.4 | <0.001 | LAT | 33.99 | 1 | <0.001 | 0.97 | 0.98 | 16.0 | 0.916 |
|  |  |  |  |  |  |  |  |  | LON | 44.11 | 1 | <0.001 |  |  |  |  |
|  |  |  |  |  |  |  |  |  | POPSYS | 13.45 | 1 | 0.008 |  |  |  |  |
|  |  | 102 | a,f,b | 56.96 | <0.001 | 96.1 | 94.4 | <0.001 | LAT | 24.77 | 1 | 0.002 | 0.03 | 0.0 | 21.0 | 0.245 |
|  |  |  |  |  |  |  |  |  | LON | 20.91 | 1 | 0.003 |  |  |  |  |
|  |  |  |  |  |  |  |  |  | POPSYS | 54.38 | 1 | <0.001 |  |  |  |  |
|  |  | 103 | f,b | 103.26 | <0.001 | 96.3 | 95.3 | 0.950 | LAT | 22.45 | 1 | 0.002 | 1.0 | 1.0 | 10.0 | 0.223 |
|  |  |  |  |  |  |  |  |  | LON | 29.95 | 1 | <0.001 |  |  |  |  |
| *ppp2r1a* | 4 | 106 | f,b | 28.03 | <0.001 | 75.7 | 73.0 | 0.086 | LAT | 28.03 | 1 | <0.001 | 0.31 | 0.14 | 23.0 | 0.170 |
|  |  | 107 | f,b | 18.19 | 0.001 | 82.0 | 77.5 | 0.984 | LON | 13.24 | 1 | 0.007 | 0.0 | 0.0 | 17.5 | 0.465 |
|  |  |  |  |  |  |  |  |  | POPSYS | 13.28 | 1 | 0.006 |  |  |  |  |
| *ppp3ca* | 2 | 109 | a,b | 4.14 | 0.066 | 67.4 | 51.1 | 0.132 | LAT | 10.14 | 1 | 0.019 | 0.67 | 0.56 | 14.0 | 0.834 |
|  |  |  |  |  |  |  |  |  | LON | 8.96 | 1 | 0.024 |  |  |  |  |
|  |  |  |  |  |  |  |  |  | POPSYS | 12.12 | 1 | 0.013 |  |  |  |  |
| *rbbp8* | 6 | 114 | f,b | 18.24 | 0.001 | 82.0 | 77.5 | 0.991 | LON | 13.24 | 1 | 0.007 | 0.0 | 0.0 | 17.5 | 0.465 |
|  |  |  |  |  |  |  |  |  | POPSYS | 13.35 | 1 | 0.006 |  |  |  |  |
| *rps6k3* | 17 | 116 | f,b | 11.46 | 0.010 | 58.9 | 53.8 | n.a. | POPSYS | 11.46 | 1 | 0.010 | 0.50 | 0.85 | 3.0 | 0.060 |
| *sgol1* | 2 | 122 | f,b | 13.07 | 0.006 | 59.2 | 54.7 | n.a. | POPSYS | 13.07 | 1 | 0.006 | 1.0 | 1.0 | 18.0 | 0.361 |
| *smc1a* | 4 | 123 | a,b | 13.11 | 0.003 | 84.9 | 78.4 | <0.001 | LAT | 16.98 | 1 | 0.004 | 0.90 | 1.0 | 7.5 | 0.103 |
|  |  |  |  |  |  |  |  |  | LON | 26.81 | 1 | 0.001 |  |  |  |  |
|  |  |  |  |  |  |  |  |  | POPSYS | 11.51 | 1 | 0.012 |  |  |  |  |
|  |  | 124 | f,b | 68.41 | <0.001 | 94.5 | 93.1 | <0.001 | LON | 127.50 | 1 | <0.001 | 0.0 | 0.0 | 20.0 | 0.223 |
|  |  |  |  |  |  |  |  |  | POPSYS | 10.29 | 1 | 0.012 |  |  |  |  |
|  |  | 125 | f,b | 68.41 | <0.001 | 94.5 | 93.1 | <0.001 | LON | 127.50 | 1 | <0.001 | 0.0 | 0.0 | 20.0 | 0.223 |
|  |  |  |  |  |  |  |  |  | POPSYS | 10.29 | 1 | 0.012 |  |  |  |  |
| *ywhaz* | 1 | 129 | f,b | 18.98 | 0.001 | 82.6 | 78.2 | 0.673 | LON | 13.69 | 1 | 0.006 | 1.0 | 1.0 | 12.5 | 0.465 |
|  |  |  |  |  |  |  |  |  | POPSYS | 13.99 | 1 | 0.006 |  |  |  |  |
| *zfp36* | 2 | 130 | f,b | 11.05 | 0.009 | 55.1 | 50.1 | n.a. | POPSYS | 11.05 | 1 | 0.009 | 1.0 | 1.0 | 21.0 | 0.136 |
|  |  | 131 | f,b | 11.05 | 0.009 | 55.1 | 50.1 | n.a. | POPSYS | 11.05 | 1 | 0.009 | 0.0 | 0.0 | 9.0 | 0.136 |

**Table S10.** Linkage disequilibrium (*p*-values) of single nucleotide polymorphisms (SNPs) selected for multilocus genotype construction. Upper right matrix: *p*-values for the *P. lessonae* data set. Lower left matrix: *p*-values for the *P. ridibundus* data set. Blue and red shaded values indicate significantly linked loci for *P. lessonae* and *P. ridibundus*, respectively.


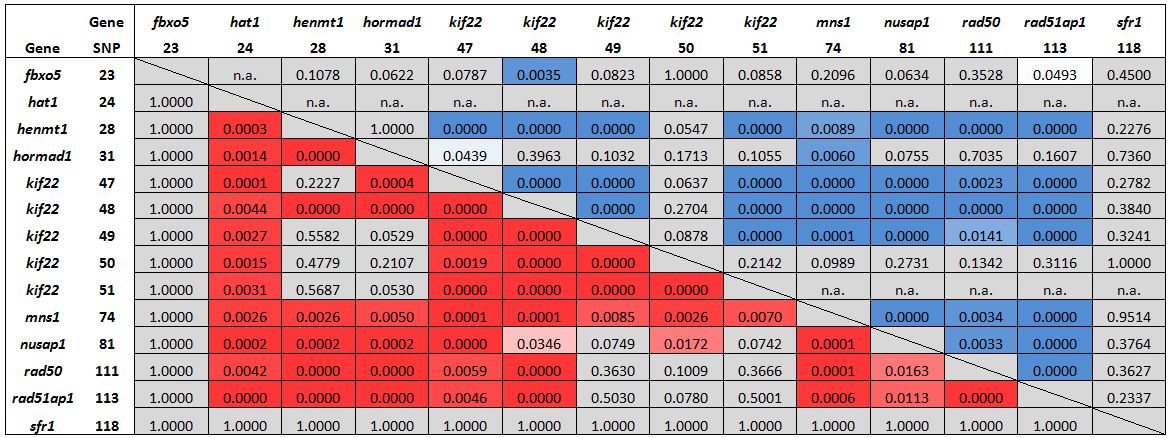


**Table S11.** Parameters of *P. lessonae* (LL) and *P. ridibundus* (RR) genome assemblies.

| **Parameter** | **Assembly** |  |  |
| --- | --- | --- | --- |
|  | **LL-1** | **LL-2** | **RR** |
| Size (including Ns) [bp] | 4894985653 | 6333496276 | 7162657451 |
| Size (without Ns) [bp] | 4041398048 | 4247649372 | 2811228707 |
| Gap Content [%] | 17.44 | 32.93 | 60.75 |
| Singletons [N] | 2844675 | 2778275 | 10787472 |
| Scaffolds [N] | 3453250 | 3155940 | 12064811 |
| Mean Size [bp] | 1417 | 2006 | 593 |
| Longest Sequence [bp] | 501104 | 1433414 | 73247 |
| Average Length of Breaks (N) in Scaffolds [bp] | 247 | 660 | 360 |
| N50 | 27865 | 136366 | 10022 |
| N90 | 1405 | 8363 | 154 |
| Scaffolds >1Kbp [N/%] | 273239/7.91 | 120999/3.83 | 593941/4.91 |
| Predicted coding sequence [Mbp] | 380 |  | 78 |
