# Supplementary material for: New insights into the molecular basis of gametogenesis in the hybridogenetic water frog Pelophylax esculentus
Source: Sci Rep. 2026 Feb 4;16:5012. doi: 10.1038/s41598-026-37515-w (PMC12876976; doi:10.1038/s41598-026-37515-w)
Supplement: Supplementary file 6 — Supplementary Material 6 [file 41598_2026_37515_MOESM6_ESM.docx]

**Plötner et al. 2026_Supplementary Material 4 (Figures S1–S4)**


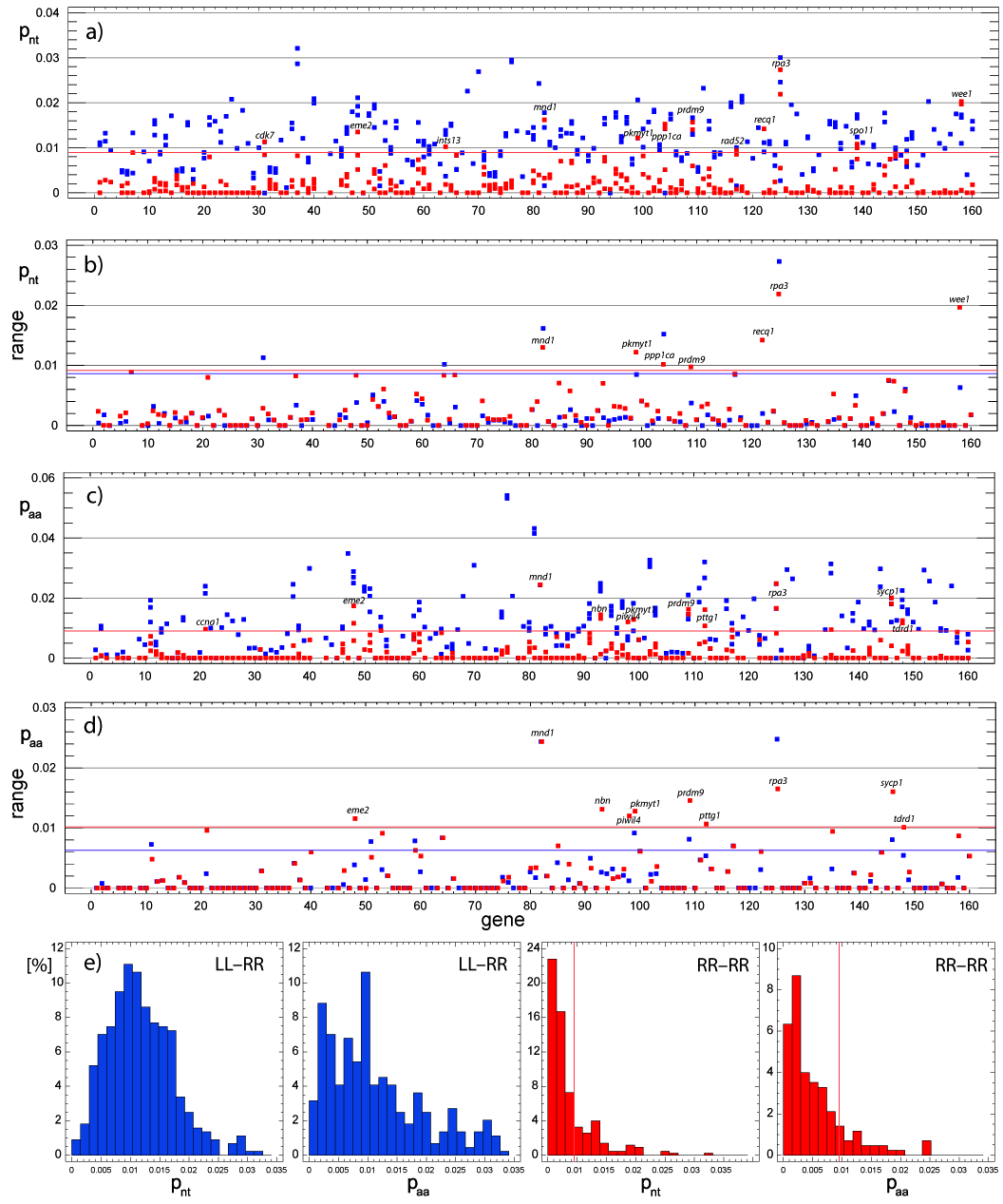


**Fig. S3** Scatterplots of pairwise uncorrected distances (a, c), based on nucleotide sequences (p_nt_) and corresponding amino acid sequences (p_aa_), their ranges of variation (b, d), and frequency distributions (e), calculated for 160 gametogenic genes. Blue: interspecific comparisons between *P. lessonae* (LL) and *P. ridibundus* (RR); red: intraspecific comparisons among *P. ridibundus* individuals (RR1/RR2, RR1/RR3, RR2/RR3). Values outside the 95% percentile (indicated by blue and red lines) correspond to genes exhibiting unusually low interspecific or unusually high intraspecific distance values. Gene names are listed in Table S1, Supplementary Material 1.


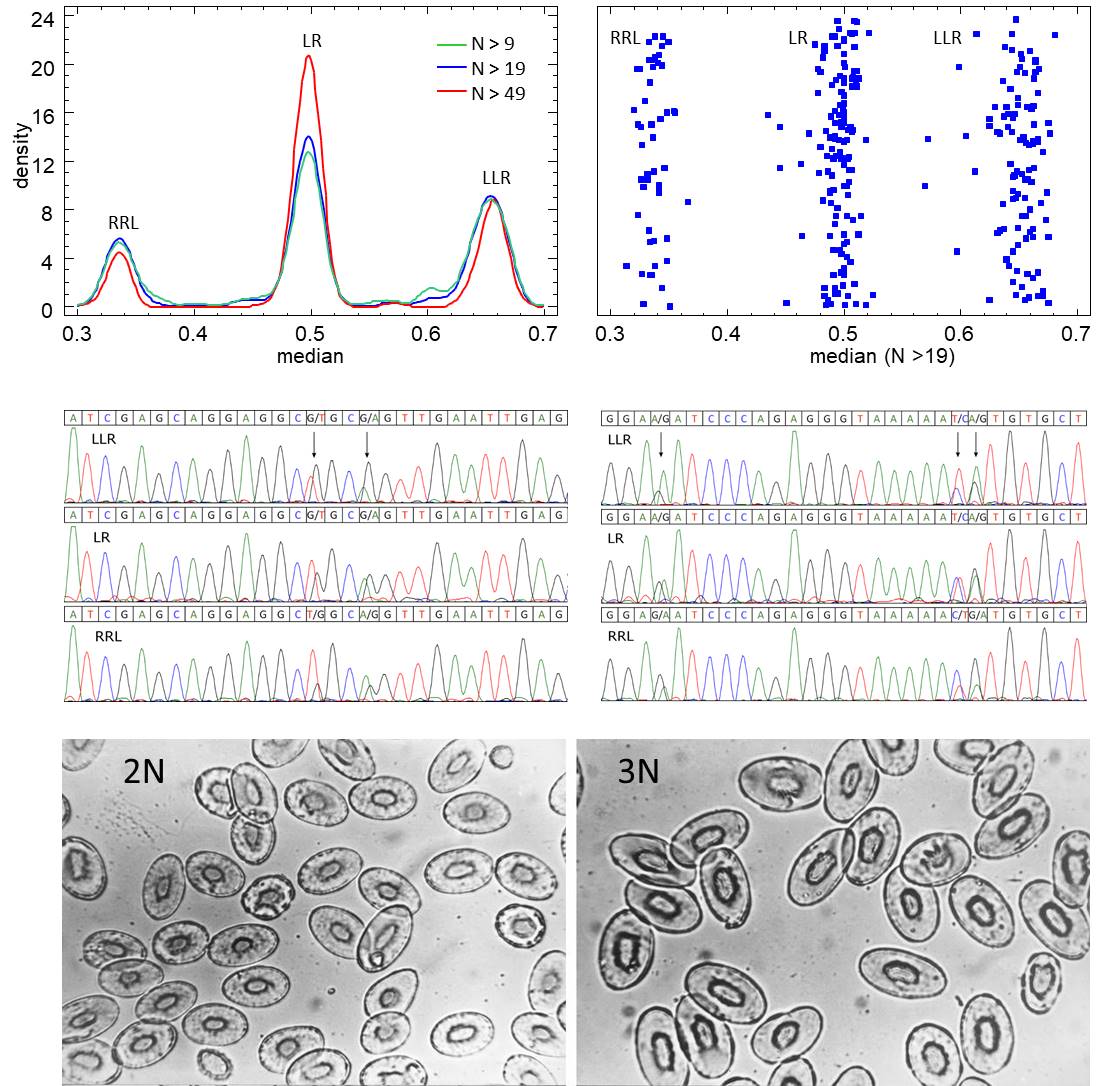


**Fig. S2** Methods used to determine the ploidy and genotype of *Pelophylax esculentus*. **Top**: Read-based genotyping. The number of L-specific reads per SNP was divided by the total coverage (L + R). Because of gene dosage effects, triploid hybrids with two L genomes and one R genome (LLR) are expected to have a higher proportion of L-specific reads (≈ 0.67), whereas diploid hybrids (LR) and triploid RRL hybrids are expected to have values of ≈0.5 and ≈0.33, respectively. The density plot (left) and scatter plot (right) are largely consistent with these expectations, even when individuals with fewer SNPs (9 < N < 20) were included. **Center:** Sanger sequence-based genotyping, as described by Tecker et al. (2017) and Krage et al. (2022), also exploits gene dosage effects. Electropherograms of *uqcrfs1* sequences show that LLR genotypes display significantly higher L-specific peaks, RRL genotypes show higher R-specific peaks at the heterozygous (species-specific) positions, and LR hybrids exhibit nearly equal L- and R-specific peak heights. **Bottom**: Ploidy determination by measuring erythrocytes (e.g., Uzzell and Berger 1975; Günther 1977). Blood smears of diploid (left) and triploid (right) hybrids (640x magnification, from Günther 1977). Erythrocytes of triploid hybrids (LLR, RRL) are, on average, one-third larger than those of diploids (LR), as caused by an additional set of chromosomes.


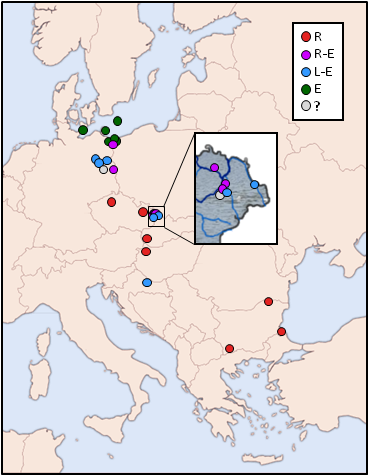


**Fig S3** Map of the sampling area.

R: all-*ridibundus* populations

R-E: *ridibundus*-*esculentus* populations

L-E: *lessonae*-*esculentus* populations

E: all-hybrid (*esculentus*) populations

?: population system unclear


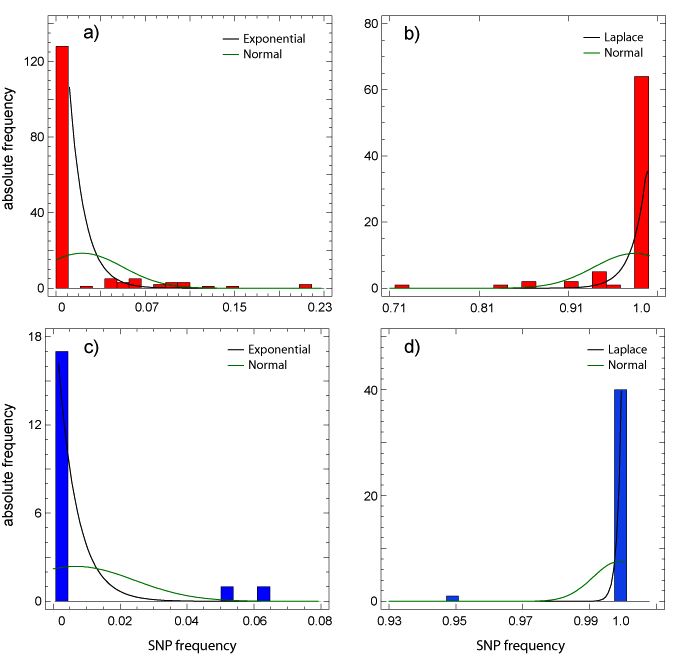


**Fig. S4** Zero-inflated (a, c) and one‑inflated (b, d) distributions of the frequencies of putative species-specific SNPs for *P. ridibundus* (red) and *P. lessonae* (blue). Frequencies deviating from 0 or 1 may indicate introgression of *lessonae*-specific alleles into the *ridibundus* gene pool, or vice versa.

**References**

Günther, R. Die Erythrozytengröße als Kriterium zur Unterscheidung diploider und triploider Teichfrösche, Rana "esculenta" L. (Anura). Biol Zentralblatt **96**, 457–466 (1977)*.*

Krage, S., Schreiber, R., John, S., Plötner, M. & Plötner, J. Morphologisch-morphometrische und genetische Untersuchungen an Wasserfröschen (*Pelophylax* spp.) im Müritz-Nationalpark (Mecklenburg-Vorpommern) unter besonderer Berücksichtigung des Kleinen Wasserfroschs (*Pelophylax lessonae*). *Z Feldherpetol* **29**, 184–208 (2022).

Tecker, A., Göcking, C., Menke, N., Schreiber, R., & Plötner, J. Neue Daten zur Morphologie, Genetik und Verbreitung der Wasserfrösche (*Pelophylax* spp.) im Münsterland (NRW) unter besonderer Berücksichtigung des Kleinen Wasserfroschs (*Pelophylax lessonae*). *Z Feldherpetol* **24**, 19–44 (2017).

Uzzell, T. & Berger, L. Electrophoretic phenotypes of *Rana ridibunda*, *Rana lessonae* and their hybridogenetic associate *Rana esculenta*. *Proc Acad Nat Sci Philadelphia* **127**, 13–24 (1975).
